# Oxygenation and Oxidation of Lignin Model Dimers by Fungal *Ortho*-Methoxyphenolases

**DOI:** 10.1021/jacs.5c22901

**Published:** 2026-02-05

**Authors:** Caio de Oliveira Gorgulho Silva, Nakul Abhay Bapat, Claire L. Bourmaud, Cecilie Nørskov Jensen, Jean Behaghel de Bueren, Jeremy Luterbacher, Anne S. Meyer, Gijs van Erven, Willem J. H. van Berkel, Mirjam A. Kabel, Jane W Agger

**Affiliations:** † Department of Biotechnology and Biomedicine, Technical University of Denmark, Søltofts Plads, Building 221, Kgs. Lyngby 2800, Denmark; ‡ Laboratory of Sustainable and Catalytic Processing, Institute of Chemical Sciences and Engineering, École Polytechnique Fédérale de Lausanne (EPFL), CH-1015 Lausanne, Switzerland; § Laboratory of Food Chemistry, 4508 Wageningen University & Research, Bornse Weilanden 9, 6708 WG Wageningen, The Netherlands; ∥ Wageningen Food & Biobased Research, Wageningen University & Research, Bornse Weilanden 9, 6708 WG Wageningen, The Netherlands

## Abstract

Lignin is the largest renewable resource for aromatics, and the quest to understand enzymatic lignin modification has never been more important. A recently recognized group of single-domain type-3 copper enzymes, named *ortho*-methoxyphenolases (*o*-MPs, EC 1.14.18.13) and previously referred to as short polyphenol oxidases (PPOs), found in filamentous fungi can sequentially *o*-hydroxylate and oxidize guaiacyl-type phenols into methoxy-*o*-quinones. A subset of these enzymes also targets syringyl-type phenols and, via an unprecedented oxidative *o*-demethoxylation mechanism, funnels these into the same methoxy-*o*-quinones generated from guaiacyl-type compounds. Here, we demonstrate that fungal *o*-methoxyphenolases also cleave bonds in lignin model dimers representing the abundant β-O-4′-linked substructures of lignin, having guaiacyl and, in some cases, syringyl terminal phenolic groups. Based on advanced liquid chromatography-mass spectrometry (LC-MS), nuclear magnetic resonance (NMR) analysis, and isotope labeling, we propose a mechanism in which the enzymatic formation of methoxy-*o*-quinone moieties in the model dimers triggers intramolecular rearrangements that lead to different types of bond cleavage, where C1–Cα cleavage predominates. Additionally, β-ether breakage and formation of Cα-ketone groups occur. We investigate the influence of pH and reductants on reaction pathways and identify strategies to steer the reaction toward either depolymerization or oxyfunctionalization of the dimers without interunit bond cleavage. The enzymes also target Cα-oxidized model dimers, albeit at lower rates. The findings of this study demonstrate the potential of using fungal *o*-methoxyphenolases for catalyzing selective *ortho*-hydroxylation and two-electron oxidation of lignin components and provide a new foundation for developing enzyme-based lignin valorization strategies.

## Graphical abstract

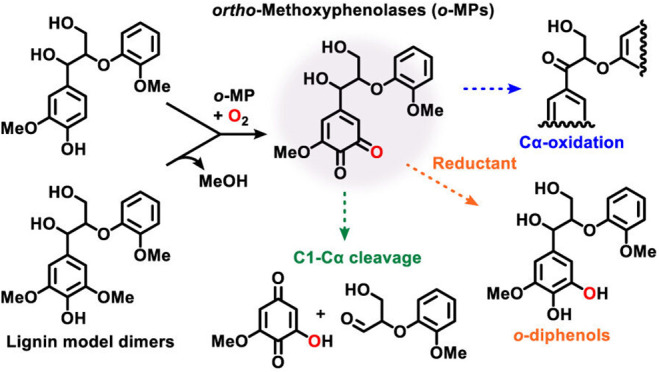

## 1 Introduction

Lignin is an immense and renewable source of aromatic carbon for high-value materials and chemicals. Yet, its potential for application remains largely untapped due to the scarcity of efficient technologies to upgrade lignin, either in its polymeric form or as the complex heterogeneous mixture of monomers and oligomers generated through depolymerization. A major challenge in biotechnology lies in developing enzymatic methods that can enable sustainable routes for lignin extraction, depolymerization, or targeted functionalization, complementing or potentially replacing existing chemical processes for lignin utilization.^1–3^

Single-domain, fungal polyphenol oxidases known as short PPOs are phylogenetically distinct from the canonical two-domain long PPOs (tyrosinases) found in fungi and other organisms. The divergence likely stems from an ancient gene duplication event that has led to two major PPO types spread across the tree of life.^4^ Fungal short PPOs can *ortho*-hydroxylate and oxidize lignin-derived guaiacyl-type compounds into their corresponding methoxy-*ortho*-quinones. In some cases, short PPOs can even attack syringyl-type compounds and, via an oxidative *ortho*-demethoxylation step, funnel these into the same methoxy-*ortho*-quinone product as when guaiacyl is the substrate.^5^ The ability of fungal short PPOs to target methoxylated lignin-derived phenols stands out among other coupled binuclear copper (CBC) enzymes, e.g., tyrosinases (EC 1.14.18.1), catechol oxidases (EC 1.10.3.1), *o*-aminophenol oxidases (EC 1.10.3.4), and *o*-aminophenol N-oxygenases and has led to the proposition of a new activity termed “*ortho*-methoxyphenolase” (*o*-MP, EC 1.14.18.13). This discovery opens new avenues for biocatalyzed oxyfuncionalization and valorization of lignin derivatives by *o*-MPs, while there are still fundamental discoveries being made within the field of CBC enzymes.^6^

In previous work, we have demonstrated the catalytic mechanism of *o*-MPs on monomeric lignin model compounds.^5^ To fully understand their biocatalytic potential in lignin valorization, it is essential to assess their activity on more structurally complex lignin analogues and eventually lignin polymers. Lignin model dimers are extremely useful to investigate enzyme reactions toward specific motifs of the lignin structure.^7^ Yet challenging analytics to elucidate the product profiles and the underlying reaction mechanisms often hinder the study of lignin-active enzymes beyond simple monomeric substrates.^8,9^

In this study, we examine in detail the activity of several new fungal *o*-MPs and a canonical fungal long PPO (tyrosinase) on a set of lignin phenolic model dimers representing the abundant β-O-4′ linked substructures of lignin. We explore variations in the phenolic terminal group (guaiacyl versus syringyl) and oxidation at the Cα position and use analogous 4-methylumbelliferyl-labeled model dimers to support our findings. We combine advanced liquid chromatography-mass spectrometry (LC-MS) and nuclear magnetic resonance (NMR) analyses and experiments with H_2_^18^O to shed light on the product profile and reaction pathways initiated by enzyme catalysis. We find that *o*-MPs can oxygenate and subsequently oxidize lignin model dimers, triggering a cascade of nonenzymatic reactions that result in bond cleavage (C1–Cα and β-ether), Cα-oxidation, or the accumulation of methoxycatechol moieties and quinones. The ultimate reaction trajectory depends on process parameters like pH and reducing agents, along with the exact variant of the enzyme, indeed providing handles to steer the reaction.

## 2 Experiments

Detailed information on the chemicals, substrate synthesis, heterologous expression of *o*-MPs, enzyme assays, and chemical analysis of catalytic products using LC-MS and NMR are provided in the Supporting Information, Materials and Methods.

## 3 Results

### 3.1 *o*-Methoxyphenolase Activity toward a Guaiacyl-Type Lignin Model Dimer

We started by testing a palette of fungal *o*-MPs toward the model dimer guaiacylglycerol-β-guaiacyl ether (GBG), which represents terminal guaiacyl phenolic sites in β-O-4′-linked substructures of lignin. The six *o*-MPs in this study originate from the saprophytic species *Myceliophthora thermophila*, also known as *Thermothelomyces thermophilus* (*Mt*PPO7, *Mt*PPO-809, and *Tt*PPO), *Chaetomium globosum* (*Cg*PPO-473 and *Cg*PPO-266), and *Parascedosporium putredinis* NO1 (*Pp*PPO-c2091), and are all predicted to be extracellular in their native host. These enzymes exhibit activity toward monomeric *o*-methoxylated phenols,^5,8,10^ and for comparative purposes, we include the canonical tyrosinase from*Agaricus bisporus* (*Ab*Tyr).^11^ The sequence identity within the characterized *o*-MPs vary moderately (between 56 and 86%), whereas the identity of *o*-MPs with *Ab*Tyr (*Ab*PPO3 isoform) is very low (11–17%) (Figure S1). Our previous work shows that the *o*-MPs included here all belong to a special and distant fungal clade within the CBC superfamily that has markedly different activities compared to the canonical fungal tyrosinases.^4^ The set of enzymes in this study represents sequence diversity that allows for comparison of catalytic variations within this clade.

All *o*-MPs transformed GBG, while *Ab*Tyr did not. Although they all catalyze the same reactions on GBG, they differ significantly in their catalytic rates and in how the product profiles are influenced by exogenous reductants (Figures 1 and S2–S5, Data set 1). Investigating the conversion rate of *Mt*PPO7, *Mt*PPO-809 and *Cg*PPO-473 exemplify how these enzymes differ in their activity toward GBG, where *Mt*PPO-809 showed the highest apparent turnover of approximately 1.6 s^–1^ (Figure S2). We propose a hypothetical reaction path on GBG based on LC-MS and NMR analyses of the reaction products, where *o*-MPs initiate the reaction by converting GBG into methoxy-*ortho*-diphenol-glycerol-β-guaiacyl ether (MDBG) via the *ortho*-hydroxylation of ring A (monophenolase activity). Subsequently, the enzymes catalyze the two-electron oxidation of MDBG to form methoxy-*ortho*-quinone-glycerol-β-guaiacyl ether (MQBG) (diphenolase activity, see Figure 1C). Beyond this stage, a series of intramolecular rearrangements leads to a combination of bond cleavage, Cα-oxidation, and oxidative couplings, as discussed below. Based on the available evidence, it is most likely that spontaneous chemistry drives these rearrangements, as nothing points toward enzyme catalysis beyond the two-electron oxidation.

**Figure 1:**
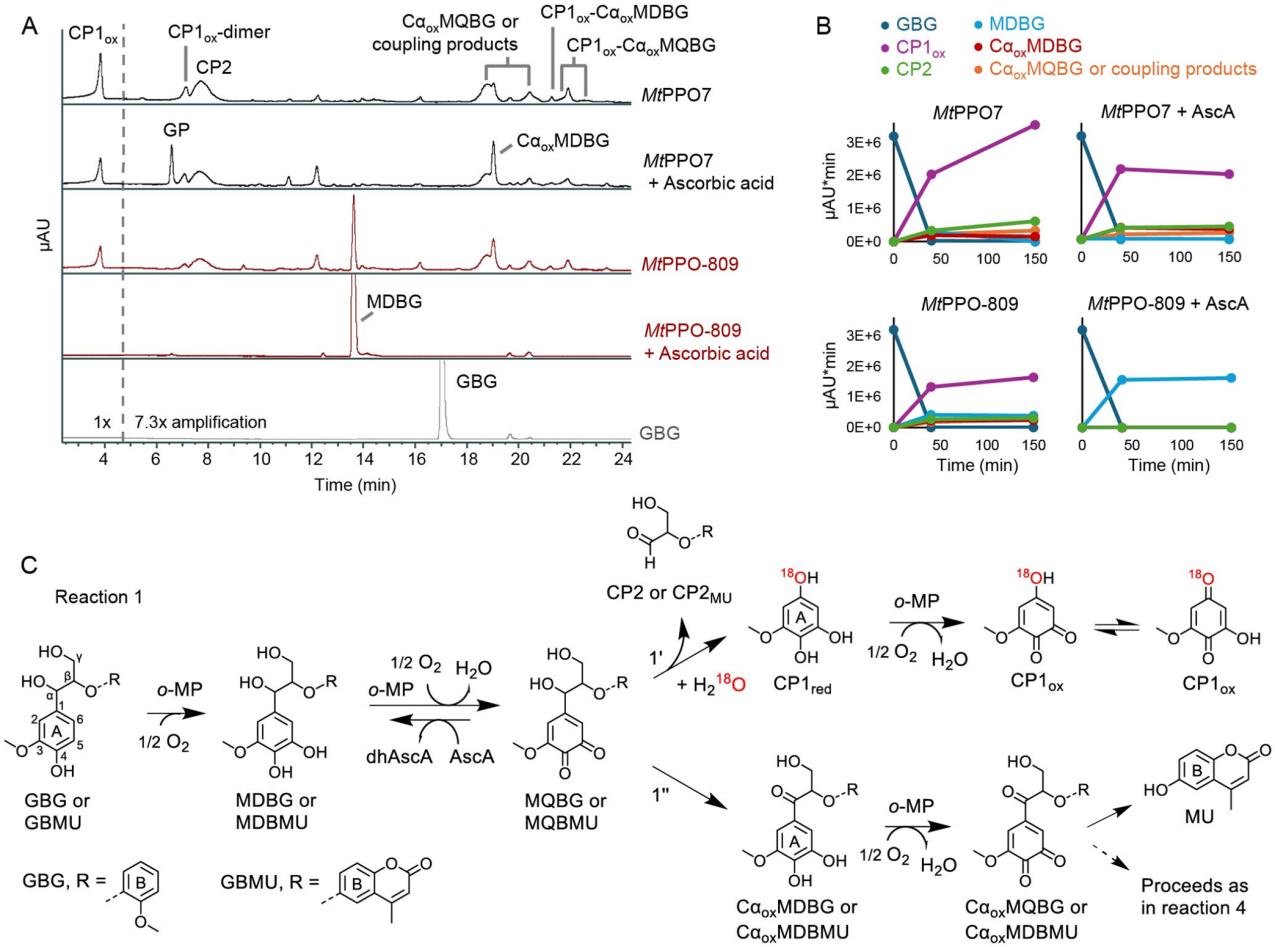
Reaction monitoring and proposed reaction scheme for *o*-methoxyphenolases (*o*-MPs) on GBG. Panels A and B highlight two enzymes from this study, as they represent different specificities toward guaiacyl- and syringyl-type substrates and different monophenolase-to-diphenolase ratios. Similar results for the remaining enzymes (*Cg*PPO-266, *Cg*PPO-473, *Tt*PPO, *Pp*PPO-c2092, and *Ab*Tyr) are found in Figures S4 and S5. (A) LC-PDA-MS chromatograms of the products of *Mt*PPO7 and *Mt*PPO-809 activities on GBG (0.2 mM). Reactions were performed in the absence or presence of ascorbic acid (1 mM) for 150 min in 20 mM sodium acetate, pH 6.0. The traces correspond to absorbance at 280 nm. Note that all chromatograms are zoomed 7.3-fold on the *Y*-axis after the retention time of 4.7 min. The data was acquired using System 1 described in Materials and Methods. (B) Time-course plots of GBG conversion by *Mt*PPO7 or *Mt*PPO-809 in the absence or presence of ascorbic acid. The relative quantification of the substrate and the main products is based on LC peak areas at 280 nm. (C) Proposed reaction pathways initiated by *o*-MP activity on GBG and guaiacylglycerol-β-4-methylumbelliferyl ether (GBMU). Pathway 1′ refers to the C1–Cα cleavage, and 1″ refers to the Cα oxidation pathway. Other abbreviations: MDBG, methoxy-*o*-diphenol-glycerol-β-guaiacyl ether; MQBG, methoxy*-o*-quinone-glycerol-β-guaiacyl ether; CP1_red_, cleavage product 1 in reduced form; CP1_ox_, cleavage product 1 in oxidized form; CP2, cleavage product 2 containing a guaiacyl moiety; Cα_ox_GBG, Cα-oxidized-guaiacylglycerol-β-guaiacyl ether; Cα_ox_MDBG, Cα-oxidized-methoxy-*o*-diphenol-glycerol-β-guaiacyl ether; Cα_ox_MQBG, Cα-oxidized-methoxy-*o*-quinone-glycerol-β-guaiacyl ether; MDBMU, methoxy-*o*-diphenol-glycerol-β-4-methylumbelliferyl ether; MQBMU, methoxy-*o*-quinone-glycerol-β-4-methylumbelliferyl ether; Cα_ox_MDBMU, Cα-oxidized-methoxy-*o*-diphenol-glycerol-β-4-methylumbelliferyl ether; Cα_ox_MQBMU, Cα-oxidized-methoxy-*o*-quinone-glycerol-β-4-methylumbelliferyl ether; CP2_MU_, cleavage product 2 containing 4-methylumbelliferyl moiety; MU, 4-methylumbelliferone; GP, putative grafting product; AscA, ascorbic acid; and dhAscA, dehydroascorbic acid. A more detailed trajectory is proposed in Figure S6.

We propose that MQBG undergoes nonenzymatic dienone-phenol rearrangement into a carbocation intermediate, which can either (i) be attacked by a water molecule at the C1 position, leading to aryl-alkyl (C1–Cα) cleavage (Figure 1C, reaction 1′) or (ii) undergo nonenzymatic deprotonation at the Cα position, leading to Cα-oxidized-MDBG via a *para*-quinone methide intermediate^12^ (Figure 1C, reaction 1″; Figure S6). The hydrolytic C1–Cα bond cleavage path will result in a Cα-aldehyde product containing the B-ring (cleavage product 2, CP2), and 2D-NMR analysis of the pool of products from *Mt*PPO7′s activity on GBG corroborates the formation of this CP2 product via detection of an aldehyde group at the Cα position, bonded to a CH group (the β carbon), which in turn is bonded to a CH_2_ group (γ position) (Figures 1C and S7). In addition to CP2, data also show evidence for a similar C1–Cα cleavage product, CP2^alk^, in which the Cα-aldehyde is adjacent to an alkene group (Figure S8).

Regarding the A-ring fragment, we propose a cleavage product (CP1), which exists predominantly in the oxidized form, CP1_ox_ (2-hydroxy-6-methoxy-1,4-benzoquinone), because the hydroquinone version (CP1_red_) is rapidly converted by the *o*-MPs’ diphenolase activity or spontaneous auto-oxidation. CP1_ox_ may be enzymatically produced as an *ortho*-quinone but likely tautomerizes into a *para*-quinone (Figure 1C). To verify the exact nature of the CP1_ox_ product, we synthesized the product via a Dakin reaction starting from 3,4-dihydroxy-5-methoxybenzaldehyde into 6-methoxybenzene-1,2,4-triol (Figure S9), whereafter spontaneous auto-oxidation into 2-hydroxy-6-methoxy-1,4-benzoquinone allowed the NMR assignment of the final product as CP1_ox_ (in *para*-quinone configuration) (Figure S10). To further verify the nature of CP1_ox_ product, we generated it from three independent enzymatic reactions: incubation of *Mt*PPO7 with 2-methoxy-1,4-benzoquinone (in the presence of ascorbic acid), with 2-methoxy-1,4-hydroquinone, and with 6-methoxybenzene-1,2,4-triol and verified retention times, UV, and MS spectra (Figures S11–S13). Exact quantification of the CP1_ox_ cleavage product proved difficult due to the transient and unstable nature of the quinone, yet based on calibration (LC-UV–vis signal, Figure S14) via the synthetic standard, we estimate the formation of CP1_ox_ to reach 67 μM during the 2.5 h reactions when *Mt*PPO7 reacts on GBG at pH 6.0 without the presence of ascorbic acid (according to the reaction in Figure S15). The reaction starts from 0.2 mM GBG; hence, the yield of CP1_ox_ corresponds to approximately 33%. It is worth noting that CP1_ox_ coupling products are also detected; hence, quantification of free CP1_ox_ alone likely underestimates total C1–Cα bond cleavage.

To obtain further evidence for the hydrolytic cleavage of the C1–Cα bond, we show the incorporation of heavy oxygen in the CP1_ox_ product when *Mt*PPO7 reactions on GBG occur in the presence of H_2_^18^O (Figures 2A and 1C). Specifically, CP1_ox_ appears as a mixture of mono-^18^O-labeled and di-^18^O-labeled CP1_ox_ products, indicating ^16^O/^18^O exchange with the solvent. We examined this exchange phenomenon by incubating the CP1_ox_ standard in H_2_^18^O and followed the nonenzymatic exchange of oxygen over time (Figure 2A), which shows that one of the oxygens is prone to exchange. Previous studies have shown that especially ketones are prone to exchange,^13^ which leads us to suggest that the single exchange we observe occurs on either the C1 or the C4 position of CP1_ox_, with C4 likely being the most susceptible site due to the activating effect of its neighboring oxygen atoms. Reaction with *Mt*PPO7 and GBG in H_2_^18^O hence results in the formation of both a single- and a double-labeled CP1_ox_ product. While the MS data do not definitely reveal the positions of the heavy oxygen atoms, they are consistent with the hypothesis that one heavy oxygen originates from hydrolysis (at the C1 position), while the other is a result of exchange with the solvent (at the C4 position). In an attempt to lower the nonenzymatic exchange rate, the experiments in Figure 2A (both control and enzyme reaction) were performed at neutral pH and without enzyme heat inactivation.

**Figure 2:**
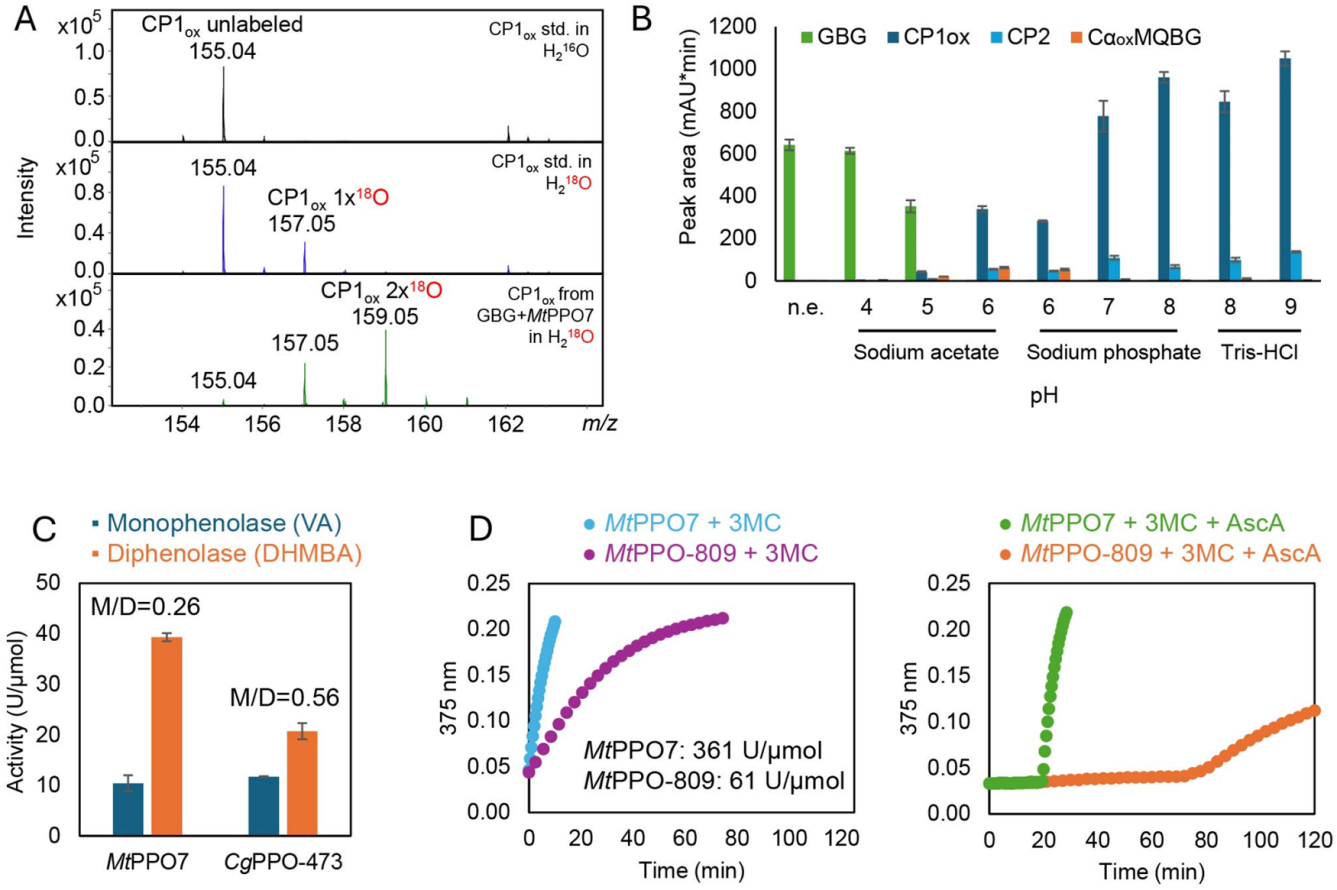
(A) Mass spectra of CP1_ox_ standard (0.3 mM) prepared in H_2_^16^O or H_2_^18^O and of CP1_ox_ generated from GBG (1 mM) following incubation with *Mt*PPO7 (5 μM) in the presence of H_2_^18^O. All reaction mixtures, including the CP1_ox_ standard, were buffered with 5 mM sodium phosphate (pH 7.0), incubated for 48 min at 25 °C, and analyzed directly by LC-MS. (B) Effect of pH on the formation of C1–Cα cleavage products (CP1_ox_ and CP2) and Cα-oxidized-methoxy-*o*-quinone-glycerol-β-guaiacyl ether (Cα_ox_MQBG) from GBG upon reaction with *Mt*PPO7 for 30 min. The relative quantification of the substrate and the products is based on LC-PDA-MS peak areas at 280 nm. A control reaction without enzyme (*n.e*.) is included. (C) Monophenolase and diphenolase activities of *Mt*PPO7 and *Cg*PPO-473 on vanillic acid (VA, 1 mM) and 3,4-dihydroxy-5-methoxybenzoic acid (DHMBA, 1 mM), respectively, at pH 6.0. Enzyme concentrations of 1.0 and 0.25 μM are used for VA and DHMBA, respectively. Specific activity rates (1 unit (U) = 1 μmol/min) are calculated based on substrate consumption – quantified by LC-PDA-MS – during the linear phase of the reaction (see Materials and Methods for further experimental details). The monophenolase-to-diphenolase (M/D) ratios are shown for each enzyme. Please refer to Table S1 for enzyme activities measured at other pH values. (D) Activities of *Mt*PPO7 and *Mt*PPO-809 (both at 0.5 μM) on 3-methoxycatechol (3-MC, 0.2 mM) in the absence (left panel) or presence (right panel) of ascorbic acid (AscA, 1 mM). Measurements are taken spectrophotometrically at 375 nm, corresponding to the formation of the chromophoric 3-methoxy-1,2-benzoquinone and/or further oxidation products. Activity rates (1 unit (U) = 1 ΔAbs_375 nm_/min) in the absence of ascorbic acid, shown in the left panel, are calculated based on the linear phase of the reaction.

As *o*-MPs are monooxygenases, it may be tempting to speculate that the C1–Cα-bond cleavage could be the result of yet another round of monooxygenation activity by the enzyme. However, our previous experiments with monomeric model compounds clearly show the incorporation of merely a single oxygen atom at the *ortho* position in experiments performed in the presence of ^18^O_2_.^5^ Furthermore, all previous literature we have come across related to PPOs (exemplified by the extensive review by Preztler et al.^14^) indicates high selectivity for the *ortho* position relative to phenolic hydroxyls, as these are prerequisites for productive substrate binding in the copper site. In all, we find it unlikely to consider that a second monooxygenation event occurs at the C1 position.

Given that aryl-alkyl cleavage likely results from nucleophilic attack by water on a highly electrophilic C1 carbocation, we hypothesized that increasing pH would favor this pathway over Cα-oxidation due to the greater availability of hydroxide ions. *Mt*PPO7 assays conducted at pH 4.0 – 9.0 reveal a trend in which the formation of CP1_ox_ and CP2 increases, while the levels of Cα-oxidized dimers decrease as pH rises (Figure 2B). This supports a mechanism in which elevated hydroxide levels enhance nucleophilic attack on the carbocation intermediate, promoting aryl-alkyl bond cleavage. Notably, this is a cautious interpretation, as *Mt*PPO7 exhibits limited activity at a pH below 6.

The other reaction trajectory (ii), as mentioned above, includes the hypothesis that MQBG can undergo rearrangement to form the Cα-oxidized, *o*-diphenolic dimer (Cα_ox_MDBG), instead of proceeding through hydrolytic C1–Cα cleavage (Figure 1C, Reaction 1″). The regeneration of the *o*-diphenol moiety in Cα_ox_MDBG allows the enzymes to catalyze yet another round of two-electron oxidation into the corresponding quinone, Cα_ox_MQBG, and both products appear in all the reactions with *o*-methoxyphenolases in this study (Figure 1A, retention times of 18.8–20.4 min; Figures S4 and S5, Data set 1). Notably, when *Mt*PPO7 – selected as a model *o*-MP – is treated with an optimized copper saturation protocol, the diphenolic intermediate (Cα_ox_MDBG) is no longer present in the product profile, whereas Cα_ox_MQBG remains detectable. This suggests that *Mt*PPO7 acquires a higher diphenolase activity and highlights the importance of copper saturation for reaction rates. The presence of Cα_ox_MQBG is further supported by its LC-MS peaks, which show absorption at λ ≈ 360 nm (Figures S16 and S17), a wavelength characteristic of quinone-containing species. Detailed HSQC and HMBC analyses of the pool of products also suggest the presence of Cα_ox_MQBG, with all expected coupling correlations of the Cα-oxidized quinone (Figure S18). Yet, due to the high complexity of the sample and the absence of an exact reference model structure, assignments remain provisional for now. Consistent with the LC-MS data from reactions with copper-saturated *Mt*PPO7, no direct couplings are observed in the HSQC and HMBC spectra that indicate the presence of the Cα-ketone diphenolic product (Cα_ox_MDBG).

The implication of the re-establishment of the diphenol is that the enzymes are then capable of extracting additional electrons and theoretically thereby continuously harvest electrons if the substrate is a large polymeric network. How such a continuous electron drain affects the stability of a polymer is an extremely interesting question, which calls for further investigations.

If MQBG generated by *o*-MPs can rearrange into Cα_ox_MDBG, then a similar event should occur when *o*-MPs oxidize the monomeric vanillyl alcohol, and indeed, *Mt*PPO7 converts vanillyl alcohol into 3,4-dihydroxy-5-methoxybenzaldehyde as the main product (Figure 3, reaction 2; Figure S19). A similar rearrangement is observed in the conversion of 3,4-dihydroxybenzyl alcohol into 3,4-dihydroxybenzaldehyde by *Ab*Tyr.^15^ All together, we conclude that *o*-MPs can mediate the Cα-oxidation of phenolic compounds with a primary or secondary alcohol at the Cα position, even though their redox activity targets the aromatic ring rather than the aliphatic side chain of their substrate.

**Figure 3:**
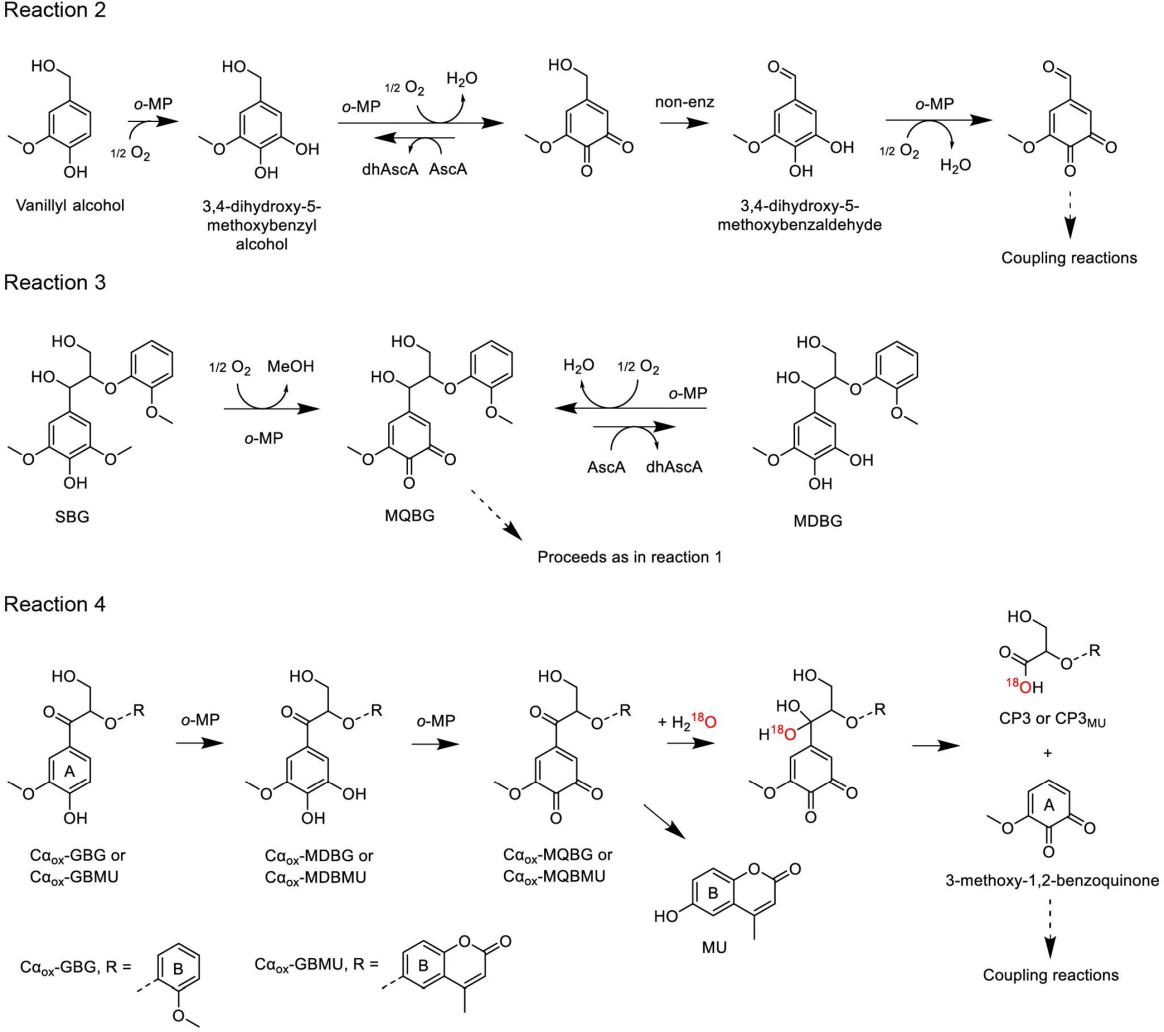
Proposed reaction pathways initiated by the activity of fungal *o*-methoxyphenolases (*o*-MPs) on various lignin model compounds, namely vanillyl alcohol (reaction 2), syringylglycerol-β-guaiacyl ether (SBG, reaction 3), Cα-oxidized-guaiacylglycerol-β-guaiacyl ether (Cα_ox_GBG, reaction 4), and Cα-oxidized-guaiacylglycerol-β-4-methylumbelliferyl ether (Cα_ox_GBMU, reaction 4). The incorporation of oxygen atoms from water into cleavage products is illustrated in reaction 4 based on experimental results using H_2_^18^O (Figure S31). SBG conversion (reaction 2) is catalyzed by *Mt*PPO7, *Cg*PPO-473, and *Cg*PPO-266 but not by the other tested *o*-MPs. Other abbreviations: MDBG, methoxy-*o*-diphenol-glycerol-β-guaiacyl ether; MQBG, methoxy*-o*-quinone-glycerol-β-guaiacyl ether; Cα_ox_MDBG, Cα-oxidized-methoxy-*o*-diphenol-glycerol-β-guaiacyl ether; Cα_ox_MQBG, Cα-oxidized-methoxy-*o*-quinone-glycerol-β-guaiacyl ether; CP3, cleavage product 3 containing a guaiacyl moiety; Cα_ox_MDBMU, Cα-oxidized-methoxy-*o*-diphenol-glycerol-β-4-methylumbelliferyl ether; Cα_ox_MQBMU, Cα-oxidized-methoxy-*o*-quinone-glycerol-β-4-methylumbelliferyl ether; CP3_MU_, cleavage product containing a 4-methylumbelliferyl moiety; MU, 4-methylumbelliferone; AscA, ascorbic acid; dhAscA, dehydroascorbic acid.

Despite the bond cleavage events, the continued activity toward cleavage products and the reactive nature of *o*-quinones lead to oxidative coupling reactions. The formation of coupling products is evident by the detection of species with molecular weights exceeding that of the starting material, and masses are consistent with the addition of one extra aromatic unit relative to GBG. Among the provisionally annotated coupling products are CP1_ox_-dimer, CP1_ox_-CP2, CP1_ox_-Cα_ox_MDBG, and CP1_ox_-Cα_ox_MQBG (Figure S20). On the other hand, we do not observe any insoluble product formation during reactions. Even after prolonged reaction times (24 h) and sample acidification following enzyme inactivation, no pellet is formed. In contrast, *Mt*PPO7 reactions with 3-methoxycatechol (3-MC) and *Ab*Tyr reactions with tyrosine clearly yield insoluble products, as evidenced by pellet formation (Figure S21). This suggests that the repolymerization of GBG cleavage products does not achieve the same degree of polymerization as that observed with the oxidation of 3-MC and tyrosine. We speculate that protection at C5 and C1 in CP1_ox_ prevents its extensive polymerization.

### 3.2 Effect of Ascorbic Acid and Hydrogen Peroxide on GBG Conversion by *o*-MPs

The addition of ascorbic acid (1 mM) accelerates GBG transformation by all *o*-MPs, likely by promoting the transition of the enzyme from its met-form to the oxy-form, the only state capable of catalyzing monophenol hydroxylation.^16^ The acceleration is particularly evident for *Cg*PPO-473, *Cg*PPO-266, and *Tt*PPO, as these exhibit slower activity on GBG in the absence of ascorbic acid compared to that of the other enzymes (Figures S3–S5). Similarly, 100 μM H_2_O_2_ can also promote the oxy-form of *o*-MPs^17,18^ and accelerates GBG conversion by *Mt*PPO7, though to a lesser extent than 1 mM ascorbic acid (Figure S15).

In the presence of ascorbic acid, MQBG is nonenzymatically reduced to MDBG, which consequently accumulates as the main product for four of the enzymes (*Cg*PPO-473, *Cg*PPO-266, *Tt*PPO, and especially *Mt*PPO-809) after 40 min incubation (Figures S3–S5). However, extended incubation time leads to reoxidation of MDBG, likely due to ascorbic acid depletion, except in reactions with *Mt*PPO-809, where MDGB remains the sole product after 150 min (Figures S3–S5). In the case of *Mt*PPO7 and *Pp*PPO-c2092, MDBG does not accumulate significantly at any time point during the reactions, even when ascorbic acid is present (Figures 1B and S3–S5), and the explanation likely lies in differences in the ratios of their monophenolase-to-diphenolase reaction kinetics. We determine monophenolase and diphenolase activities of *Cg*PPO-473 and *Mt*PPO7 (representing either *o*-MPs with low or high diphenolase activity, respectively) by measuring the consumption of vanillic acid and its corresponding *o*-diphenol, 3,4-dihydroxy-5-methoxybenzoic acid (DHMBA). At pH 6.0, the same condition used for assays on GBG, *Mt*PPO7 exhibits higher diphenolase activity on DHMBA (39.3 U/μmol) than *Cg*PPO-473 (20.7 U/μmol), while their monophenolase activities on vanillic acid are comparable (10.4 and 11.7 U/μmol, respectively). The monophenolase-to-diphenolase ratio for *Cg*PPO-473 is therefore approximately 0.56, compared to 0.26 for *Mt*PPO7 (Figure 2C and Table S1).

Ascorbic acid can effectively prevent or reverse 3-methoxycatechol (3-MC) oxidation by both *Mt*PPO-809 and *Mt*PPO7 (Figure 2D). Yet the latency in 3-methoxy-1,2-benzoquinone formation is longer in reactions with *Mt*PPO-809 than *Mt*PPO7, and the specific activity of *Mt*PPO7 on 3-MC is approximately 6 times higher than the specific activity of *Mt*PPO-809. Altogether, *Mt*PPO-809, *Cg*PPO-473, *Cg*PPO-266, and *Tt*PPO convert the *o*-diphenol MDBG at a lower rate compared to *Mt*PPO7 and *Pp*PPO-c2092, explaining the differences in MDGB accumulation in the presence of ascorbic acid.

Notably, in cases in which no or only minor MDGB accumulation occurs, ascorbic acid introduces other changes in the reaction. This is evident from unidentified peaks in the chromatograms, which may result from grafting of ascorbic acid-derived structures onto GBG-derived compounds (Figures 1A and S5).

### 3.3 *o*-MP Activity toward a Syringyl-Type Lignin Model Dimer

Evaluation of the same enzymes on syringylglycerol-β-guaiacol ether (SBG), a model dimer featuring a syringyl unit as the A-ring, shows that only three *o*-MPs – namely *Mt*PPO7, *Cg*PPO-473, and *Cg*PPO-266 – are active on SBG, and that conversion occurs only in the presence of ascorbic acid or H_2_O_2_ (Figures S3 and S22–S24). This likely reflects the need to promote the enzyme from its met-form to the oxy-form. Although these enzymes’ ability to attack SBG is consistent with previous findings on monomeric syringyl-type compounds, neither ascorbic acid nor H_2_O_2_ is required for its activity on syringol, syringic acid, or sinapic acid.^5^

The product profile of *o*-MPs activity on SBG closely resembles that of GBG conversion (Figures S22–S24). Based on the proposed mechanism of *Mt*PPO7 activity on monomeric syringyl-type compounds,^5^ we hypothesize that *o*-MPs convert SBG directly into MQBG through oxygenation and demethoxylation at position 3 or 5 of ring A, with methanol as a coproduct (Figure 3, reaction 3). Once MQBG is formed, the reaction evolves in the same way as the reactions on GBG (Figure 1C).

In reactions with *Cg*PPO-473 and *Cg*PPO-266 and in the presence of ascorbic acid, MDBG accumulates as the predominant product for at least 40 min, after which it undergoes further oxidation to MQBG, likely due to ascorbic acid depletion. In contrast, MDBG does not accumulate significantly with *Mt*PPO7 (Figures S3 and S22–S24), resulting from its higher diphenolase activity (Figure 2C–D). Enzyme reactions without ascorbic acid or H_2_O_2_ yield only trace amounts of Cα_ox_SBG, comparable to levels in control reactions with free CuSO_4_, which suggests catalysis by free copper released upon enzyme inactivation (Figures S3 and S24).

### 3.4 *o*-MP Activity on 4-Methylumbelliferyl-Labeled Model Dimer

The lignin model dimer guaiacylglycerol-β-4-methylumbelliferyl ether (GBMU)^19^ facilitates the identification of *o*-MP catalytic products because it contains different ring A and ring B structures (Figure 1C). Comparison between *Mt*PPO7 reaction products on GBMU and GBG reveals similarities and corroborates the hydrolytic aryl-alkyl (C1–Cα) cleavage pathway, evidenced by the presence of CP1_ox_, CP2_MU_, and CP2_MU_^alk^. Cα-oxidized-methoxy-*o*-quinone-glycerol-β-MU (Cα_ox_MQBMU) also appears to exist, along with potential coupling products such as the CP1_ox_ dimer and CP1_ox_-CP2_MU_ (Figures S25 and S26). Importantly, the release of 4-methylumbelliferone (MU) (Figures 1C and S25) suggests β-ether bond cleavage^20^ corresponding to about 2.5 to 5% of the reactions taking place, making β-O-4 cleavage a minor reaction pathway compared to C1–Cα cleavage. Additionally, β-ether cleavage products are not obvious from the *o*-MPs activity on GBG. We propose two possible explanations for this difference, which are not necessarily mutually exclusive.

Either β-O-4′ cleavage does occur in GBG reactions but goes undetected due to subsequent oxidation and repolymerization of the resulting cleavage products. Notably, β-O-4′ cleavage products of GBG comprising the A-ring or the B-ring (having methoxycatechyl or guaiacyl moieties, respectively) would be favorable substrates for *o*-MPs activity, and some of the coupling products we observe may have such fragments incorporated (Figure S17). The low activity of *Mt*PPO7 on nonmethoxylated phenols like MU explains why the MU unit released from β-ether cleavage does not undergo further oxidation and repolymerization. As such, this represents another advantage of using GBMU for providing insights into bond cleavage events that are difficult to observe with GBG as a substrate.

The alternative explanation is that the electron-withdrawing carbonyl group in the MU unit may increase susceptibility to β-ether bond cleavage upon enzymatic oxidation by facilitating electron delocalization and lowering the energy barrier for ether cleavage. The structure of GBMU resembles engineered *Arabidopsis* lignin containing scopoletin linked via β-O-4′ bonds to lignin units.^21^ This engineered lignin type is more susceptible to β-ether bond cleavage under alkaline conditions than natural lignin. Given the structural similarity between MU and scopoletin (6-methoxyumbelliferone), a comparable effect on β-O-4′ bond lability is reasonable to expect. Other studies have also shown that a carbonyl functionality at the Cα’ position of the B unit in lignin dimers enhances β-O-4′ bond cleavage under alkaline conditions,^22,23^ and this structural feature is also present in GBMU. While our reactions proceed at pH 6.0, the presence of an electron-withdrawing group in the MU unit may similarly promote β-ether bond cleavage through a distinct mechanism facilitated by local electron deficiency immediately after enzymatic oxidation. With this in mind, it is relevant to consider whether GBMU fairly represents lignin chemistry, as its reactivity may overestimate enzymatic efficiency in β-O-4′ bond cleavage compared to natural lignin.

Activity on monomeric model compounds that have different *para*-aliphatic chains further demonstrates that the aliphatic chain also influences how the *o*-quinone, formed via *o*-MP activity, evolves and drives specific bond cleavage events. *Mt*PPO7 activity on guaiacylpropane-1,2-diol does not lead to C1–Cα bond cleavage but, instead, promotes Cα-Cβ cleavage and Cα oxidation, yielding 3,4-dihydroxy-5-methoxybenzaldehyde as the major product (Figure 4, reaction 5). In contrast, activity on guaiacylpropane-1,2-dione leads to both Cβ-Cγ and Cα-Cβ cleavage, yielding hydroxy-acetovanillone (1-(3,4-dihydroxy-5-methoxyphenyl)­ethan-1-one) and 3,4-dihydroxy-5-methoxybenzoic acid (Figure 4, reaction 6), respectively. Meanwhile, activity on vanillyl alcohol, vanillin, and acetovanillone does not lead to any type of bond cleavage (Figure 3, reaction 2; Figure 4, reactions 7 and 8).

**Figure 4:**
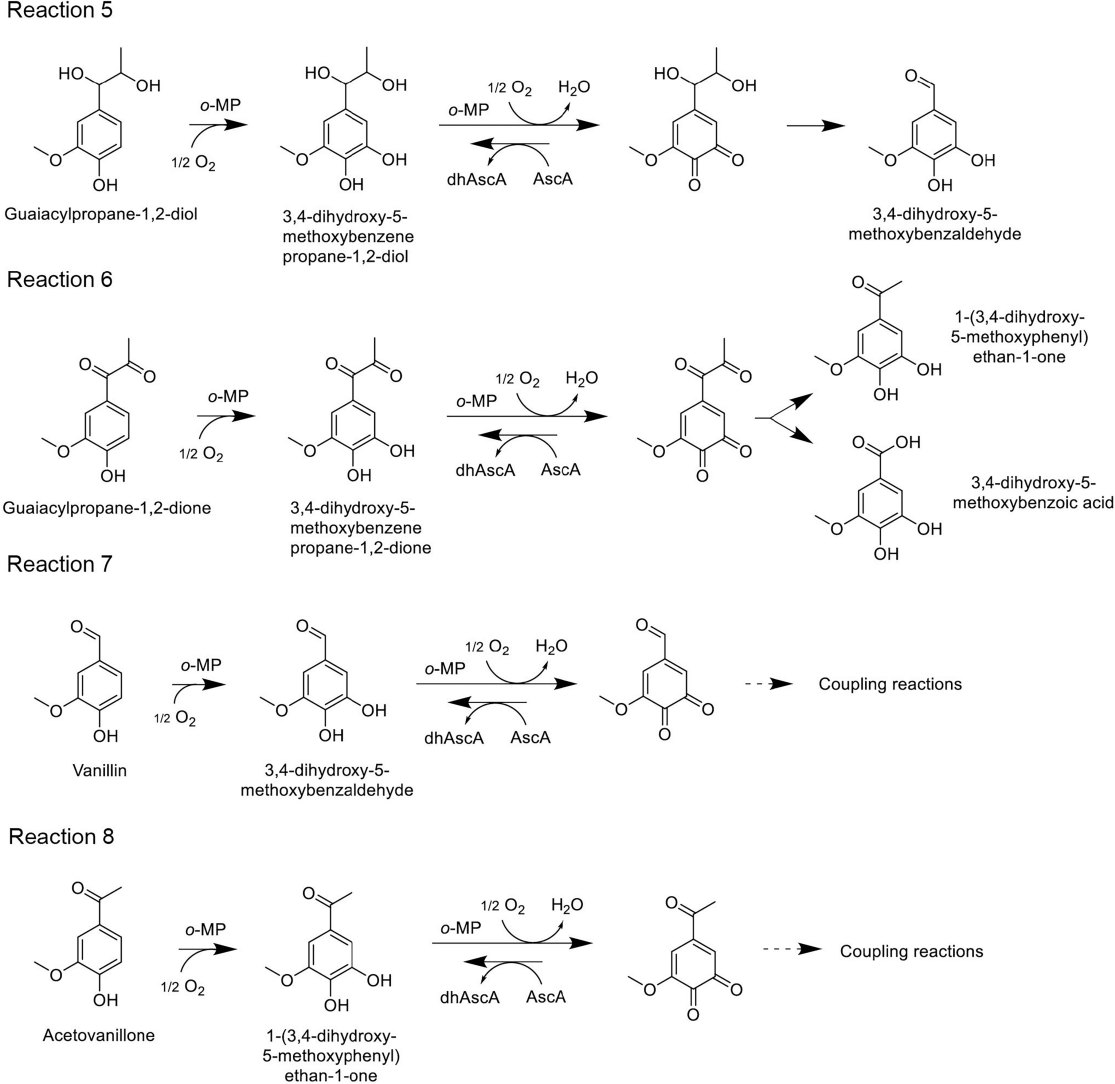
Proposed reaction pathways initiated by *Mt*PPO7 activity on guaiacylpropane-1,2-diol (reaction 5), guaiacylpropane-1,2-dione (reaction 6), vanillin (reaction 7), and acetovanillone (reaction 8). AscA, ascorbic acid; dhAscA, dehydroascorbic acid.

### 3.5 *o*-MP Activity on Cα-Oxidized Model Dimers

As Cα-oxidation is a frequent lignin modification introduced by ligninolytic fungi^20,24^ and is associated with an increased propensity for chemocatalytic bond cleavage,^25^ investigating *o*-MP activity on Cα-oxidized species is of particular relevance. Chemically synthesized Cα-oxidized GBG (Cα_ox_GBG) serves as a substrate for *Mt*PPO7, and the conversion happens at a lower rate compared to unmodified GBG (Figures S15 and S28). Initially, Cα_ox_GBG undergoes hydroxylation at the *ortho* position of ring A, forming Cα_ox_MDBG (Figure 3, reaction 4), and is then subsequently oxidized into Cα_ox_MQBG. The formation of this *o*-quinone likely increases the electrophilicity of the Cα carbon, rendering it more susceptible to nucleophilic attack by water, and we observe C1–Cα bond cleavage and the generation of cleavage product CP3, which retains the B-ring and features a carboxyl group at the Cα position.

^31^P NMR analysis of the product pool from *Mt*PPO7́s activity on Cα_ox_GBG reveals a small COOH signal (ca. 2.3 mol % of the substrate), absent in buffer-only control reactions, which may correspond to the CαOOH group in CP3 (Figure S30 and Table S2). In reactions with H_2_^18^O, the incorporation of ^18^O into CP3 supports a mechanism involving water-mediated attack at the Cα (Figure S31). The complementary C1–Cα cleavage product containing ring A – presumed to be 3-methoxy-1,2-benzoquinone – was not detected in its free form. However, the presence of multiple coupling products likely incorporating this species suggests its transient formation (Figure S29).

The absence of CP1 and CP2 from Cα_ox_GBG oxidation is due to their formation via water attack at C1 following the generation of MQBG from GBG, an intermediate that does not occur in the Cα_ox_GBG oxidation pathway. On the other hand, traces of CP3 are also released from GBG oxidation (Figure S32) as a result of water attack at the Cα of the Cα_ox_MQBG intermediate, confirming that the reaction pathways for GBG and Cα_ox_GBG partially overlap.

*Mt*PPO7 activity on the analogous Cα_ox_GBMU substrate further substantiates the same reaction path leading to C1–Cα bond cleavage via hydrolytic attack to Cα, yielding CP3_MU_ and 3-methoxy-1,2-benzoquinone (Figure 3, reaction 4; Figures S25 and S28). Additionally, we again detect free MU, which implies β-ether bond cleavage. The amount of MU released from Cα_ox_GBMU was similar to that from GBMU (Figures S25 and S33), suggesting that prior Cα-oxidation is neither required nor facilitates β-ether bond cleavage by *o*-MP action.

### 3.6 Cα-Oxidation Retards *o*-MP Activity

*Mt*PPO7 activity on Cα-oxidized dimers Cα_ox_GBG and Cα_ox_GBMU is slower than that on GBG and GBMU (Figures S28 and S33). Similarly, *Mt*PPO7 activity on Cα-oxidized monomeric substrates such as vanillin, guaiacylpropane-1,2-dione, and acetovanillone is slower compared to activity on the non-Cα-oxidized counterparts vanillyl alcohol and guaiacylpropane-1,2-diol (Figures S34–38). This is likely due to the electron-withdrawing effect of the carbonyl group at Cα, which decreases electron density in the aromatic ring and consequently raises the redox potential of these compounds,^26^ and the lower electron density in the ring will effectively slow down hydroxylation by *o*-MPs.

The Cα-carbonyl’s inhibitory effect on the *o*-MP activity further extends into the two-electron oxidation step (the diphenolase activity), as Cα-oxidized *o*-diphenols such as 3,4-dihydroxy-5-methoxybenzaldehyde generated from vanillyl alcohol and vanillin accumulate after reaction with *Mt*PPO7 (Figures S34–38). The same type of accumulation occurs with the *o*-hydroxylated products of Cα_ox_GBG and Cα_ox_GBMU (Figures S25 and S28). This aligns with findings on *Streptomyces glaucescens* tyrosinase activity toward *para*-substituted phenols,^6,27^ which indicate that electron-withdrawing *para* groups slow down *o*-diphenol oxidation to *o*-quinone.

## 4 Discussion

The key finding of this study is that fungal *o*-methoxyphenolases exhibit activity toward model dimers that represent phenolic β-O-4′-linked substructures of lignin, where they catalyze sequential *ortho*-hydroxylation and two-electron oxidation of guaiacyl and, in some cases, syringyl terminal phenolic groups. Our results also meticulously inform about the reaction trajectories that follow enzyme-catalyzed oxidation, a cascade of spontaneous, nonenzymatic reactions.

The nonenzymatic reaction trajectory is affected by pH and the presence of reducing agents. C1–Cα-bond breakage is favored at elevated pH (pH 7–9), and MDBG accumulates in the presence of a reducing agent. The accumulation of MDBG is strongest in cases where *o*-MP has low diphenolase activity. The possibility to accumulate methoxylated *o*-diphenols in certain conditions is interesting in itself, since the increase in vicinal phenolic OHs can enhance the reactivity of lignin oligomers and monomers for further chemical functionalization^28,29^ and thereby improve application as core components of functional materials such as lignin-based epoxy resins.^30–32^

While C1–Cα bond cleavage appears to predominate in our results (represented by a least 33% formation of CP1_ox_ from GBG in *Mt*PPO7 reactions), Cα-Cβ or even β-O-4′ cleavage is certainly possible, yet it depends highly on the nature of the B-ring and on the oxidation state of the Cα-position.

Despite cleavage events, the reactive nature of the *o*-quinones formed by *o*-MPs also leads to oxidative coupling reactions of the dimers and their cleavage products and likely also grafting reactions with reducing agents if these are present. The fact that some of the cleavage products themselves are substrates for the enzyme not only complicates the interpretation of the reaction pathway but also contributes to additional coupling reactions. It is worth noting that, in a fungal degradation system, the small soluble quinones that form are likely to be taken up immediately by the fungus, preventing their polymerization. From an application perspective, simultaneous depolymerization and repolymerization represent a major bottleneck in the enzymatic deconstruction of lignin or its oligomers by oxidoreductases. Developing strategies to mitigate this should be a focus of future research. Yet, depending on their stability, the quinones that form upon extensive oxidation of the dimers may find application, e.g., as electron carriers for energy storage applications, as they can undergo reversible two-electron reactions.^33^

Canonical lignin-modifying enzymes like laccases and peroxidases that perform one-electron oxidation generally tend to form polymerization products via the C5 (*ortho*) position on guaiacyl units.^9,34,35^ However, protection of this *ortho* position by a methoxy-group (in S-units) appears to favor Cα-oxidation and bond cleavage over oxidative polymerization, also from one-electron oxidations.^12,35–38^ Hence, the special ability of the *o*-MPs to introduce a hydroxylation exactly in the *ortho* position offers a new type of substitution of this position and thereby the possibility to steer reactions toward bond cleavage and Cα-oxidation rather than coupling reactions.

Interestingly, two recent studies suggest GBG cleavage by tyrosinases without the essential hydroxylation,^39,40^ and in one study, the reaction appears greatly accelerated by the presence of excess copper sulfate.^40^ To our knowledge, such activity by a tyrosinase is unprecedented and should be investigated further.

The annotation of the reaction products on the lignin model dimers is challenging due to the high complexity of the product profiles (including formation of cleavage products that act as substrates), combined with the struggle to distinguish isobaric compounds and the lack of authentic standards. The strategic use of a diverse set of lignin model compounds, including monomers and dimers (some bearing 4-methylumbelliferyl units), facilitates the characterization of *o*-MP-catalyzed reactions and product annotation. Complementary NMR analyses can further confirm the presence of specific functional groups and, together with isotopic labeling experiments, support the proposal of plausible reaction pathways initiated by these enzymes.

Whether *o*-methoxyphenolases are active on polymeric, insoluble lignin remains unanswered at this point, and investigating this is beyond the scope of our study. Nevertheless, it is certain that these enzymes can catalyze the necessary chemistry to modify the subunits in lignin, even in larger subunits. Actual activity on polymeric lignin, though, will depend on the enzyme’s ability to cross the liquid–solid interface and the accessibility of the phenolic units. *o*-MP activity is strictly dependent on the presence of free phenolic groups, and considering the effective concentration of these in native polymeric lignin (<20%),^41^ it is clear that activity may be rather limited. Yet, chemocatalytic depolymerization of lignin also likely initiates at free phenolic sites.^42^ Importantly, the introduction of the vicinal phenolic hydroxyl opens the opportunity of pumping electrons out of the lignin polymer because continuous reorganization and reconstitution of the phenolic hydroxyls will allow continued *o*-MP-catalyzed two-electron oxidation of the hydroxylated ring structure. It is interesting to note that the major cleavage event, the C1–Cα cleavage (as we observe it on the model dimers), will effectively reduce the residual concentration of phenols in lignin, as it will not generate a new phenolic OH group on the remaining polymer. Hence, this type of cleavage event will effectively stop *o*-MP activity and not result in a potential peeling mechanism suggested, for example, for the action of laccases.^43^ If *o*-MPs are to substantially contribute to lignin depolymerization, then cleavage events must occur at the β-O-4′ bond, thereby releasing a new phenolic end group that can sustain further enzyme activity. *o*-MPs may display high applicability for oxyfunctionalization of lignin fractions, where the phenolic content is high, and the molecular weight is low.

## 5 Conclusions

The current study establishes that *o*-MP-catalyzed oxygenation and oxidation promote bond cleavage in lignin model dimers, and they introduce unique chemical features. *o*-MPs are therefore new and highly interesting candidates for oxyfunctionalization of lignin-derived compounds, and these findings open new research avenues for studying a hitherto unexplored enzyme type within lignin conversion or valorization.

## Supplementary Material





## Notes

Summary of the most abundant RP-UHPLC-PDA-ESI-MS peaks arising from the reaction of lignin model dimers with fungal *o*-methoxyphenolases (XLSX)
Materials and methods; amino acid sequence identity (%) among the enzymes characterized in this study (Figure S1); GBG consumption by *Mt*PPO7, *Mt*PPO-809, and *Cg*PPO-473 (Figure S2); time-course plots of GBG and SBG conversion by *o*-MPs (Figure S3); RP-UHPLC-PDA-ESI-MS chromatograms of the reaction products from *o*-MP-catalyzed oxidation of guaiacylglycerol-β-guaiacyl ether (GBG) (Figure S4); RP-UHPLC-PDA-ESI-MS chromatograms of the reaction products from *o*-MP-catalyzed oxidation of guaiacylglycerol-β-guaiacyl ether (GBG) in the presence of ascorbic acid (Figure S5); detailed reaction pathways initiated by *o*-methoxyphenolases on guaiacylglycerol-β-guaiacyl ether (GBG) (Figure S6); 2D ^1^H–^13^C HSQC and HMBC spectra and COSY spectrum of the pool of products of *Mt*PPO7 activity on GBG emphasis on the cleavage product CP2 (Figure S7); 2D ^1^H–^13^C HSQC and HMBC spectra and COSY spectrum of the pool of products of *Mt*PPO7 activity on GBG, emphasis on a Cα-aldehyde group (Figure S8); NMR assignment of CP1_red_ (Figure S9); NMR assignment of CP1_ox_ (Figure S10); independent enzymatic routes for the production of CP1_ox_ from three different substrates (Figure S11); verification of enzymatic production of CP1_ox_ from four different substrates (Figure S12); MS1 and MS2 spectra and UV–vis spectra of CP1_ox_ authentic standard and of that produced from *Mt*PPO7 activity on GBG (Figure S13); LC–UV chromatograms of the CP1_ox_ synthetic standard at varying concentrations (Figure S14); time-course plots of *Mt*PPO7 activity on GBG and Cα_ox_GBG (Figure S15); RP-UHPLC-PDA-ESI-MS chromatograms of the products of *Mt*PPO7 reaction on GBG. Traces relative to absorbance at λ = 280 nm and λ = 360 nm (Figure S16); hypothetical structures for the peaks at retention times of 18.8 and 20.4 min in Figure S16 (Figure S17); 2D ^1^H–^13^C HSQC and HMBC spectra of the pool of products of *Mt*PPO7 activity on GBG (Figure S18); RP-UHPLC-PDA-ESI-MS chromatograms of the products of *Mt*PPO7 reaction on vanillyl alcohol (Figure S19); hypothetical structures of coupling products generated by *Mt*PPO7 activity on GBG (Figure S20); visual inspection of the formation of water-insoluble products (Figure S21); RP-UHPLC-PDA-ESI-MS chromatograms of the reaction products from *o*-MP-catalyzed oxidation of SBG (Figure S22); RP-UHPLC-PDA-ESI-MS chromatograms of the reaction products from *o*-MP-catalyzed oxidation of SBG in the presence of ascorbic acid (Figure S23); time-course plots of *Mt*PPO7 activity on SBG (Figure S24); RP-UHPLC-PDA-ESI-MS chromatograms of *Mt*PPO7 activity on GBMU and Cα_ox_GBMU (Figure S25); hypothetical structures of some coupling products or cleavage products generated by *Mt*PPO7 activity toward GBMU (Figure S26); hypothetical structures of some coupling products generated by *Mt*PPO7 activity toward Cα_ox_GBMU (Figure S27); RP-UHPLC-PDA-ESI-MS chromatograms of the products of *Mt*PPO7 activity on Cα_ox_GBG (Figure S28); hypothetical structures of coupling products generated during *Mt*PPO7 reaction toward Cα_ox_GBG (Figure S29); ^31^P NMR spectra of the pool of products of *Mt*PPO7 activity on Cα_ox_GBG (Figure S30); study on the incorporation of ^18^O from H_2_^18^O in the cleavage product CP3 from Cα_ox_GBG upon reaction with *Mt*PPO7 (Figure S31); detection of CP3 in *Mt*PPO7 reactions with Cα_ox_GBG and GBG (Figure S32); time course of *Mt*PPO7 activity on GBMU and Cα_ox_GBMU (Figure S33); *Mt*PPO7 activity on monomeric guaiacyl-type compounds (Figure S34); RP-UHPLC-PDA-ESI-MS chromatograms of *Mt*PPO7 reaction toward vanillin (Figure S35); RP-UHPLC-PDA-ESI-MS chromatograms of *Mt*PPO7 reaction toward 3,4-dihydroxy-5-methoxybenzaldehyde (Figure S36); RP-UHPLC-PDA-ESI-MS chromatograms of *Mt*PPO7 reaction toward acetovanillone (Figure S37); RP-UHPLC-PDA-ESI-MS chromatograms of *Mt*PPO7 reaction toward guaiacylpropane-1,2-diol (Figure S38); RP-UHPLC-PDA-ESI-MS chromatograms of *Mt*PPO7 reaction toward on guaiacylpropane-1,2-dione (Figure S39); monophenolase and diphenolase activities of *Mt*PPO7 and *Cg*PPO-473 on vanillic acid and 3,4-dihydroxy-5-methoxybenzoic acid (Table S1); ^31^P NMR quantification of aliphatic, phenolic, and carboxylic hydroxyl groups of dimer model compounds (Table S2) (PDF)
