# Supplementary material for: Oxygenation and Oxidation of Lignin Model Dimers by Fungal Ortho-Methoxyphenolases
Source: J Am Chem Soc. 2026 Feb 5;148(6):6727–38. doi: 10.1021/jacs.5c22901 (PMC12921834; doi:10.1021/jacs.5c22901)
Supplement: Supplementary file 2 [file ja5c22901_si_002.pdf]

Supporting information for:

Oxygenation and oxidation of lignin model dimers by fungal *ortho*-methoxyphenolases

Caio de Oliveira Gorgulho Silva[1], Nakul Abhay Bapat[2], Claire L. Bourmaud[2], Cecilie Nørskov Jensen[1], Jean Behaghel de Bueren[2], Jeremy Luterbacher[2], Anne S. Meyer[1], Gijs van Erven[3,4], Willem J. H. van Berkel[3], Mirjam A. Kabel[3], Jane W. Agger[1]*

[1]Department of Biotechnology and Biomedicine, Technical University of Denmark, Søltofts Plads, Building 221, Kgs. Lyngby 2800, Denmark

[2]Laboratory of Sustainable and Catalytic Processing, Institute of Chemical Sciences and Engineering, École Polytechnique Fédérale de Lausanne (EPFL), CH-1015 Lausanne, Switzerland

[3]Laboratory of Food Chemistry, Wageningen University & Research, Bornse Weilanden 9, 6708 WG Wageningen, The Netherlands

[4]Wageningen Food & Biobased Research, Wageningen University & Research, Bornse Weilanden 9, 6708 WG Wageningen, The Netherlands

*Corresponding author

Table of Contents

Materials and methods ... 4
Chemicals ... 4
Synthesis of 6-methoxybenzene-1,2,4-triol ($CP1_{red}$) ... 4
Auto-oxidation of $CP1_{red}$ to $CP1_{ox}$ ... 5
Synthesis of guaiacyl-propane-1,2-dione ... 5
Synthesis of guaiacyl-propane-1,2-diol ... 6
Synthesis of $C\alpha_{ox}$GBG ... 7
Synthesis of 4-methylumbelliferyl-labeled dimers (GBMU and $C\alpha_{ox}$GBMU) ... 7
Enzyme production ... 10
Copper saturation of recombinant fungal *o*-methoxyphenolases (*o*-MPs) ... 10
Enzyme assays on dimers ... 11
Enzyme assays on monomeric lignin model compounds ... 11
Enzyme assays in $H_2{}^{18}O$ ... 11
Effect of pH on GBG cleavage by *Mt*PPO7 ... 12
Quantification of monophenolase and diphenolase activities of *o*-MPs on vanillic acid and 3,4-dihydroxy-5-methoxybenzoic acid ... 12
Effect of ascorbic acid on diphenolase activity of *o*-MPs towards 3-methoxycatechol ... 12
Quantification of GBG conversion rates by *o*-MPs ... 13
RP-UHPLC-PDA-ESI-MS analysis of enzyme assays ... 13
Nuclear Magnetic Resonance (NMR) analysis of enzymatic reaction products ... 14
Quantification of $CP1_{ox}$ in the standard solution and in enzymatic reactions ... 15
SI Figures ... 16
Figure S1. Amino acid sequence identity (%) among the enzymes used in this study ... 16
Figure S2. GBG consumption by *Mt*PPO7, *Mt*PPO-809 and *Cg*PPO-473. ... 17
Figure S3. Time-course plots of GBG and SBG conversion by *o*-MPs ... 18
Figure S4. RP-UHPLC-PDA-ESI-MS of reaction products from *o*-MP-catalyzed oxidation of GBG ... 19
Figure S5. RP-UHPLC-PDA-ESI-MS of reaction products from *o*-MP-catalyzed oxidation of GBG in the presence of ascorbic acid ... 20
Figure S6. Detailed proposed reaction pathways initiated by activity of fungal *o*-MPs on GBG. ... 21
Figure S7. 2D ^1H–^{13}C HSQC and HMBC spectra and COSY spectrum of the pool of products of *Mt*PPO7 activity on GBG, with emphasis on the cleavage product CP2 ... 22
Figure S8. 2D ^1H–^{13}C HSQC and HMBC spectra and COSY spectrum of the pool of products of *Mt*PPO7 activity on GBG, with emphasis on a Cα-aldehyde group ... 24
Figure S9. NMR assignment of $CP1_{red}$ ... 26
Figure S10. NMR assignment of $CP1_{ox}$ ... 28
Figure S11. Independent enzymatic routes for the production of $CP1_{ox}$ from three different substrates ... 33
Figure S12. Verification of enzymatic production of $CP1_{ox}$ from four different substrates ... 34

Figure S13. MS1 and MS2 spectra and UV-vis spectra of $CP1_{ox}$ authentic standard and of that produced from *Mt*PPO7 activity on GBG. 35

Figure S14. LC-UV chromatograms of the $CP1_{ox}$ synthetic standard at varying concentrations........ 36

Figure S15. Time-course plots of *Mt*PPO7 activity on GBG and $C\alpha_{ox}$GBG. 37

Figure S16. RP-UHPLC-PDA-ESI-MS of the products of *Mt*PPO7 reaction on GBG. Traces relative to absorbance at λ=280 nm and λ=360 nm........ 38

Figure S17. Hypothetical structures for LC-PDA-MS peaks. 39

Figure S18 2D ^{1}H–^{13}C HSQC and HMBC spectra of the pool of products of *Mt*PPO7 activity on GBG........ 40

Figure S19. RP-UHPLC-PDA-ESI-MS of the products of *Mt*PPO7 reaction on vanillyl alcohol. 43

Figure S20. Hypothetical structures of coupling products generated by *Mt*PPO7 activity on GBG.. 44

Figure S21. Visual inspection of the formation of water-insoluble products........ 45

Figure S22. RP-UHPLC-PDA-ESI-MS of reaction products from *o*-MP-catalyzed oxidation of SBG 46

Figure S23. RP-UHPLC-PDA-ESI-MS of reaction products from *o*-MP-catalyzed oxidation of SBG in the presence of ascorbic acid........ 47

Figure S24. Time-course plots of *Mt*PPO7 activity on SBG. 49

Figure S25. RP-UHPLC-PDA-ESI-MS of products from *Mt*PPO7 activity on GBMU and $C\alpha_{ox}$GBMU........ 50

Figure S26. Hypothetical structures of coupling products or cleavage products generated by *Mt*PPO7 activity towards GBMU 51

Figure S27. Hypothetical structures of coupling products generated by *Mt*PPO7 activity towards $C\alpha_{ox}$GBMU. 52

Figure S28. RP-UHPLC-PDA-ESI-MS of products from *Mt*PPO7 activity on $C\alpha_{ox}$GBG........ 53

Figure S29. Hypothetical structures of coupling products generated during *Mt*PPO7 reaction towards $C\alpha_{ox}$GBG..... 54

Figure S30. ^{31}P NMR spectra of the pool of products of *Mt*PPO7 activity on $C\alpha_{ox}$GBG 55

Figure S31. Study on the incorporation of ^{18}O from $H_2{}^{18}O$ in the cleavage product CP3 from $C\alpha_{ox}$GBG upon reaction with *Mt*PPO7 56

Figure S32. Detection of CP3 in *Mt*PPO7 reactions with $C\alpha_{ox}$GBG and GBG. 57

Figure S33. Time course of *Mt*PPO7 activity on GBMU and $C\alpha_{ox}$GBMU. 58

Figure S34. *Mt*PPO7 activity on monomeric guaiacyl-type compounds 59

Figure S35. RP-UHPLC-PDA-ESI-MS of *Mt*PPO7 reaction towards vanillin. 60

Figure S36. RP-UHPLC-PDA-ESI-MS of *Mt*PPO7 reaction towards 3,4-dihydroxy-5-methoxybenzaldehyde. 61

Figure S37. RP-UHPLC-PDA-ESI-MS of *Mt*PPO7 reaction towards acetovanillone 62

Figure S38. RP-UHPLC-PDA-ESI-MS of *Mt*PPO7 reaction towards guaiacylpropane-1,2-diol. 63

Figure S39. RP-UHPLC-PDA-ESI-MS of *Mt*PPO7 (2 µM) reaction towards on guaiacylpropane-1,2-dione........ 64

SI tables 65

Table S1. Monophenolase and diphenolase activities of *Mt*PPO7 and *Cg*PPO-473 on vanillic acid and 3,4-dihydroxy-5-methoxybenzoic acid 65

Table S2. ^{31}P NMR quantification of aliphatic, phenolic and carboxylic hydroxyl groups in dimer model compounds 66

SI References........ 67

Materials and methods

Chemicals

Guaiacylglycerol-β-guaiacyl ether (GBG) was purchased from TCI Chemicals (Tokyo, Japan). Syringylglycerol-β-guaiacyl ether (SBG, also named 1-(4-hydroxy-3,5-dimethoxyphenyl)-2-(2-methoxyphenoxy)propane-1,3-diol) was purchased from abcr Gute Chemie (Karlsruhe, Germany). Vanillyl alcohol, vanillin, vanillic acid, acetovanillone, 3,4-dihydroxy-5-methoxybenzoic acid, 3,4-dihydroxy-5-methoxybenzaldehyde, 2-methoxy-1,4-hydroquinone, 3-methoxycatechol, and superoxide dismutase from bovine erythrocytes were purchased from Sigma-Aldrich (St. Louis, MO, USA). 2-Methoxy-1,4-benzoquinone was purchased from BLDPharma (Shanghai, China). $H_2{}^{18}O$ (^{18}O, 97%) was purchased from Cambridge Isotope Laboratories, Inc. (Xenia, OH, USA).

Synthesis of 6-methoxybenzene-1,2,4-triol ($CP1_{red}$)

3,4-Dihydroxy-5-methoxybenzaldehyde was purchased from BLD Pharma, sodium percarbonate was purchased from Sigma-Aldrich, and THF was purchased from Thermo Scientific.

6-Methoxybenzene-1,2,4-triol ($CP1_{red}$) was synthesized from 3,4-dihydroxy-5-methoxybenzaldehyde via a Dakin reaction as depicted below, following a previously described methodology for 2-methoxy-1,4-hydroquinone synthesis from vanillin.[1]

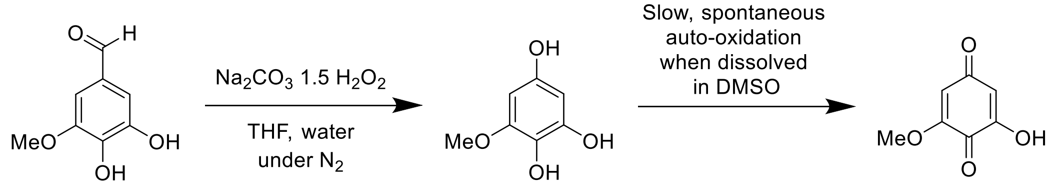

3,4-dihydroxy-5-methoxybenzaldehyde | 6-methoxybenzene-1,2,4-triol ($CP1_{red}$) | 2-hydroxy-6-methoxy-1,4-benzoquinone ($CP1_{ox}$)

In a 10 mL glass tube with a stirring bar, 100 mg 3,4-dihydroxy-5-methoxybenzaldehyde (1 equivalent) were dissolved in 2 mL THF and 0.8 mL Milli-Q water. The solution was degassed using nitrogen and sodium percarbonate (2 molar equivalents, 187.2 mg) was added under nitrogen and agitation. The reaction was stirred at room temperature and monitored using GC-MS. One extra addition of sodium percarbonate (1 molar equiv., 93.6 mg) after 2 h was required for complete substrate conversion. After a total of 3 h, the reaction was quenched by the stepwise addition of 0.3 N HCl until ~pH 3.0. THF was then evaporated under vacuum, and the aqueous phase was extracted three times with ethyl acetate. The extract was dried with $MgSO_4$ and evaporated under vacuum. Addition of chloroform followed by evaporation under vacuum was employed to remove any residual ethyl acetate.

$CP1_{red}$ characterization: **^{1}H NMR** (400 MHz, DMSO) δ 8.59 (s, 1H, O*H*), 8.57 (s, 1H, O*H*), 7.39 (s, 1H, O*H*), 5.87 (s, 2H, ArC*H*), 3.67 (s, 3H, OCH_3). **^{13}C NMR** (101 MHz, DMSO) δ 150.40, 149.49, 146.88, 126.90, 96.45, 92.05, 55.96. **^{13}C DEPT 135 NMR** (101 MHz, DMSO) δ 96.45, 92.04, 55.96. NMR spectra are shown in Figure S9.

Auto-oxidation of $CP1_{red}$ to $CP1_{ox}$

$CP1_{red}$, when dissolved in DMSO-d_6 for NMR analysis, slowly and spontaneously oxidized into $CP1_{ox}$ (2-hydroxy-6-methoxy-1,4-benzoquinone). We estimate that 15% is converted to $CP1_{ox}$ after 1 day and almost complete conversion is observed after two weeks.

$CP1_{ox}$ characterization: **^{1}H NMR** (500 MHz, DMSO) δ 5.89 (d, J = 2.4 Hz, 1H), 5.78 (d, J = 2.4 Hz, 1H), 3.74 (s, 3H). **^{13}C NMR** (126 MHz, DMSO) δ 188.17, 178.40, 157.40, 156.38, 108.54, 107.54, 56.81. **^{13}C DEPT 135 NMR** (126 MHz, DMSO) δ 108.54, 107.54, 56.81. NMR spectra are shown in Figure S10.

Synthesis of guaiacyl-propane-1,2-dione

tert-Butyl hydroperoxide solution (70% in water) was purchased from ABCR. tetrabutylammonium iodide (>99%) and 1,2,4,5-tetrachloro-3-nitrobenzene (TCNB, TraceCERT®) were purchased from Sigma Aldrich. Sodium thiosulfate (99%) and sodium borohydride (>98%) were purchased from Chemie Brunschwig AG. Hydrochloric acid (37 wt%), ethanol (99.8%), methanol (99.8%), and potassium carbonate (anhydrous, 98.5%) were purchased from Fischer Chemicals. Dichloro(*p*-cymene) Ru(II) dimer was purchased from Fluorochem. Isoeugenyl acetate was purchased from Toronto Research Chemical Inc. Ethyl acetate and toluene were purchased from Thommen-Furler. Magnesium sulfate (anhydrous, 98%) was purchased from VWR Chemicals.

1-(4-hydroxy-3-methoxyphenyl)-1,2-propane dione (guaiacyl-propane-1,2-dione) was synthesized using a modified procedure from literature.[2]

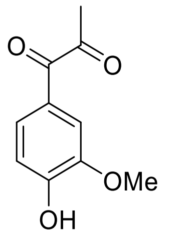

Prod. Code: RFON129
Purity (NMR): 98.2 wt%
Aspect: bright yellow crystalline solid

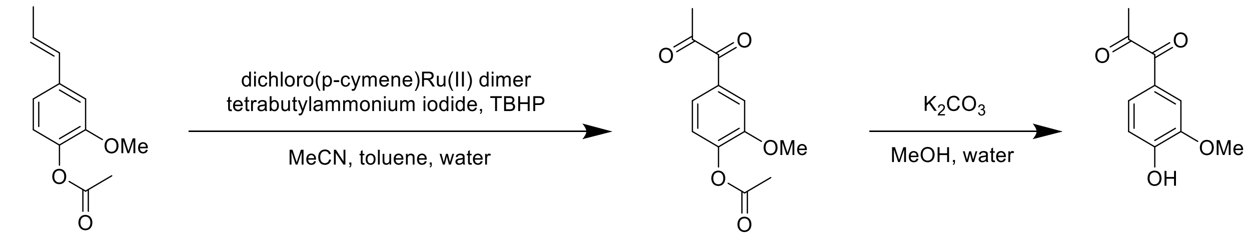

Isoeugenyl acetate (10 g, 48 mmol, 1.0 equiv.), toluene (150 mL), acetonitrile (150 mL), water (35 mL), tetrabutylammonium iodide (5.6 g, 15 mmol, 0.31 equiv.), dichloro(*p*-cymene)Ru(II) dimer (346 mg , 0.565 mmol, 1.5 mol%), and a PTFE coated stir bar were added sequentially in a 1 L round bottom flask equipped with a condenser and an addition funnel. t-Butyl hydroperoxide

(50 mL, 70% in water, 361 mmol, 7.4 eq.) was added dropwise to the stirred reaction solution at room temperature. After 45 min stirring, a saturated solution of sodium thiosulfate (300 mL) was introduced. The organic layer was separated and then concentrated in vacuo, yielding a black viscous oil. This crude was purified by flash chromatography. The appropriate fractions were collected and concentrated in vacuo to afford 1-(4-acetoxy-3-methoxyphenyl)-1,2-propane dione as a yellow crystalline solid (4.26 g, 36%).

1-(4-hydroxy-3-methoxyphenyl)-1,2-propane dione was prepared by deprotection of the acetylated product. 1-(4-acetoxy-3-methoxyphenyl)-1,2-propane dione (4.26 g, 18.0 mmol, 1.00 equiv.) was dissolved in MeOH (100 mL). The obtained solution was introduced in a 250 mL round bottom flask with a PTFE coated stir bar. Deionized water (50 mL) and K_2CO_3 (6.19 g, 44.8 mmol, 2.48 eq.) were then sequentially added. The reaction was stirred at room temperature for 1 h then neutralized with 6 N HCl (14.9 mL, 89.4 mmol, 4.97 equiv.). The neutralized solution was concentrated in vacuo to remove the methanol and then transferred to a 500 mL separatory funnel. The aqueous phase was extracted with ethyl acetate (3 x 50 mL). The organic layers were then combined, washed with brine (50 mL), dried with $MgSO_4$, and concentrated in vacuo. The product was purified by flash chromatography to afford 1-(4-hydroxy-3-methoxyphenyl)-1,2-propane dione as a yellow crystalline solid (2.24 g, 60%). The purity of the final product (98.2 wt%) was evaluated by ^{1}H-NMR using TCNB as internal standard.

Synthesis of guaiacyl-propane-1,2-diol

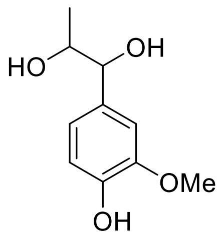

Prod. Code: RFON121

Purity (NMR): 90.5 wt%

Aspect: very viscous oil - transparent and uncoloured.

Note: the lower purity result from the difficulties of drying such a viscous paste. The main impurity is most probably ethyl acetate that comes from the work up process.

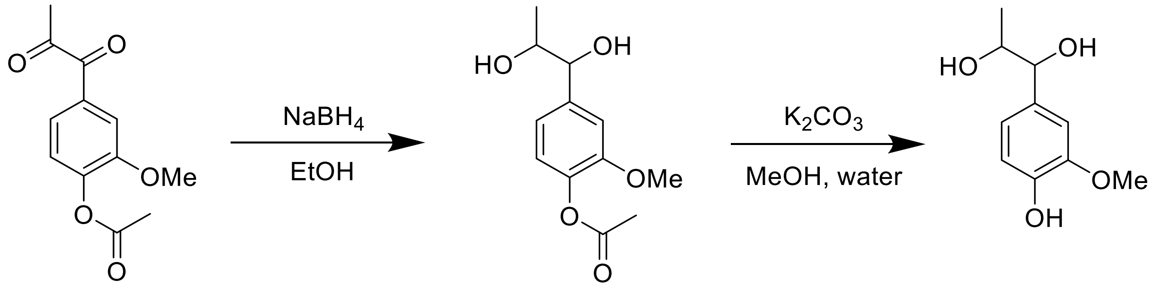

1-(4-Acetoxy-3-methoxyphenyl)-1,2-propane dione (2.46 g, 10.4 mmol, 1.00 eq.) was dissolved in ethanol (25.7 mL) and the resulting solution was transferred in a 100 mL round bottom flask with a PTFE coated stir bar and cooled down in an ice bath. Sodium borohydride (478 mg, 12.6

mmol, 1.21 eq.) was then added slowly, to avoid excessive gas release. The reaction was stirred at 0°C for 1h then quenched with 10 mL of water. The resulting solution was diluted with water (150 mL) and extracted with ethyl acetate (3 x 150 mL). The organic layers were combined, washed with brine, dried with $MgSO_4$ and concentrated in vacuo to obtain 4-(1,2-dihydroxypropyl)-2-methoxyphenyl acetate (2.23 g, 89.2%) as a crude product.

1-(4-hydroxy-3-methoxyphenyl)propane-1,2-diol was prepared by deprotection of the crude acetylated product without further purification. The crude product (2.23 g, 9.28 mmol, 1 eq.) was dissolved in a methanol (51.5 mL) and water (25.7 mL) mixture and transferred in a 250 mL round bottom flask with a PTFE coated stir bar. Potassium carbonate (3.19 g, 23.1 mmol, 2.48 eq.) was then added and the reaction was stirred at RT for 1h before being neutralized with 6N HCl 6M (7.67 mL, 46 mmol, 4.96 eq.). The neutralized solution was concentrated in vacuo to remove the methanol and then transferred to a 500 mL separatory funnel. The resulting solution was diluted with water (100 mL) and brine (50 mL) before being extracted with ethyl acetate (3 x 250 mL). The organic layers were combined, washed with brine, dried with $MgSO_4$ and concentrated in vacuo. The product was purified by flash chromatography to afford 1-(4-hydroxy-3-methoxyphenyl)propane-1,2-diol as a viscous colorless oil (1.43 g, 70.3%).

The purity of the final product (90.5 wt%) was evaluated by 1H-NMR using TCNB as internal standard.

Synthesis of Cα_{ox}GBG

100 mg (1 eq) of the GBG dimer model compound and 2 eq of 2,3-dichloro-5,6-dicyano-1,4-benzoquinone (DDQ, Sigma-Aldrich) were added to a 15 mL glass vial containing 2 mL of dichloromethane. The reaction mixture was vigorously stirred overnight at room temperature. The reaction mixture was then evaporated under reduced pressure. The crude product was purified over silica gel chromatographic column, with solvent gradient: starting from ethyl acetate/hexane 1:1 v/v to 100% ethyl acetate. The identity of Cα_{ox}GBG was verified by HRMS (ESI/QTOF) and HSQC NMR.

HRMS (ESI/QTOF) *m/z*: $[M + Na]^+$ Calcd for $C_{17}H_{18}NaO_6^+$ 341.0996; Found 341.1005.

^{1}H NMR (500MHz, $CDCl_3$) : δ 7.67 (1H, dd, *J* = 8.2, 2.4 Hz), 7.60 (1H, d, *J* = 2.3 Hz), 7.02 (1H, t, *J* = 7.8 Hz), 6.94 (2H, m, *J* = 8.5 Hz), 6.86 (2H, -, *J* = 2.3 Hz), 5.40 (1H, dd, *J* = 6.6, 3.8 Hz), 4.05 (2H, m), 3.96 (3H, s), 3.86 (3H, s).

Synthesis of 4-methylumbelliferyl-labeled dimers (GBMU and Cα_{ox}GBMU)

Thin layer chromatography (TLC) was done on Merck Aluminum TLC plates, silica gel 60 coated with fluorescent indicator F254. Flash column chromatography was performed using glass columns of varying sizes packed with Merck Geduran 60 Angstrom silica gel (40-64 µm particles) as the stationary phase.

Analytic UPLC-MS analysis was run on Waters AQUITY UPLC system equipped with PDA and SQD electrospray MS detector. Column: Kinetex 1.7 µm XB-C18, 2.1 x 50 mm. Column temperature: 50°C. Flowrate: 0.50 mL/min. Eluent A (0.1% HCOOH in H_2O) and B (0.1% HCOOH in MeCN) used in a linear gradient (5% B to 100% B) in 4.8 min, hold 0.1 min, total run time 5.0 min.

NMR spectra were recorded on a Bruker Ascend spectrometer with a Prodigy cryoprobe operating at 400 MHz for ^{1}H NMR and 101 MHz for ^{13}C NMR using the residual non-deuterated

solvent as internal standards ($CDCl_3$, ^{1}H: 7.26 ppm, ^{13}C: 77.16 ppm; DMSO-d_6, ^{1}H: 2.50 ppm, ^{13}C: 39.52 ppm). Chemical shifts (δ) are reported in ppm downfield from an internal Standard of tetramethylsilane (δ = 0) and coupling constants (J) are reported in Hz.

Preparative HPLC was performed using REVELERIS Prep Purification System by GRACE, equipped with a diode array detector using a C_{18} column (5 µM; flow 20 mL/min). Eluents A (0.1% HCOOH in H_2O) and B (0.1% HCOOH in ACN).

GBMU and Cα_{ox}GBMU substrates were synthesized as described previously[3] with modifications, as described below.

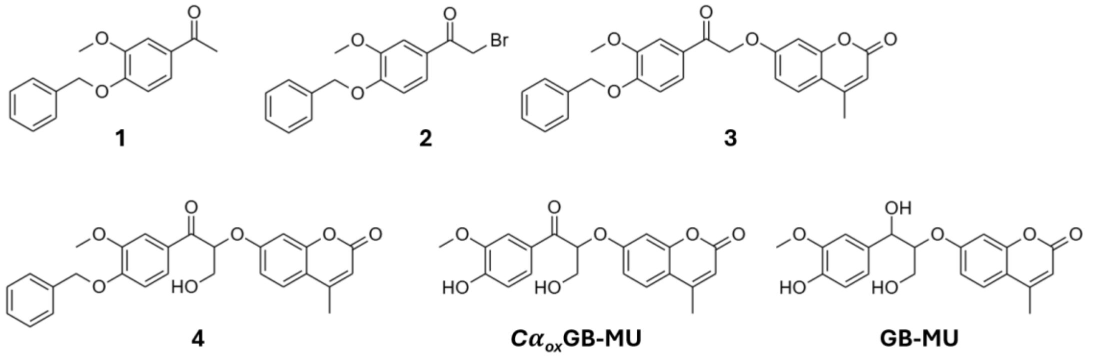

1-(4-hydroxy-3-methoxyphenyl)ethan-1-one [1]

To acetovanilone (10 g, 0.0602 mol, 1 eq) dissolved in MeOH (150 mL), NaOH (3.74 g, 0.0935 mol, 1.5 eq) and benzyl chloride (37.926 g,0.301 mol, 5 eq) was added and the reaction proceeded with stirring at room temperature. After 4h, additional benzyl chloride was added (7.5852 g, 0.0602 mol, 1 eq). Reaction was monitored by TLC (40:60 EtOAc/heptane). After 7 h, the reaction was stopped and MeOH removed under N_2 flow. EtOAc (300mL) was added to the concentrate and the mixture was washed with brine and extracted with EtOAc (2x300mL). The combined organic phase was dried over Na_2SO_4, filtered, and concentrated under reduced pressure. The crude product was purified by flash column chromatography using a graduated solvent system, DCM:MeOH (v/v, 100:0 to 98.5:1.5). White crystals were afforded (12.68 g, yield 82%). **^{1}H NMR** (400 MHz, $CDCl_3$) δ 7.55 (d, J = 4, 1H), 7.5 (dd, J = 8.2 Hz, 2 Hz, 1H), 7.43 (m, 2H), 7.35 (m, 3H), 6.89 (d, J = 8 Hz, 1H), 5.24 (s, 2H), 3.95 (s, 3H), 2.55 (s, 3H). **^{13}C NMR** (101 MHz, $CDCl_3$) δ 196.82, 152.40, 149.50, 136.29, 130.73, 128.69, 128.12, 127.18, 123.08, 112.11, 110.51, 70.80, 56.07, 26.22.

1-(4-(benzyloxy)-3-methoxyphenyl)-2-bromoethan-1-one [2]

To a solution of [1] (4.0 g, 0.0156 mol, 1 eq) in dry ACN (350 mL), *p*-toluenesulfonic acid (1.5 g, 0.0234 mol, 1.5 eq) was added and the reaction mixture was heated to reflux. NBS (0.555 g, 3.1 mmol, 0.20 eq) was added over 5 times with an interval of 10 min. The reaction was carried out under reflux and argon atmosphere and monitored by TLC (90:10 toluene/ACN). After 3h, NBS (0.555 g, 3.1mmol, 0.20 eq) was added and after a total of 5h the reaction was stopped. ACN was removed under reduced pressure. Concentrate was dissolved in DCM (500 mL), washed with brine (500 mL) which was extracted with DCM (2x500 mL). The combined organic phases were dried over Na_2SO_4, filtered, and concentrated under reduced pressure. Crude product was purified by flash column chromatography using a graduated solvent system, toluene:heptane (v/v, 100:20 to 100:0) followed by toluene:ACN (v/v, 100:0 to 100:5). Light pink crystals were afforded (4.03 g.

yield 77%). **^{1}H NMR** (400 MHz, $CDCl_3$) δ 7.57 – 7.51 (m, 2H), 7.45 – 7.30 (m, 6H), 6.91 (d, J = 8.3 Hz, 1H), 5.24 (s, 2H), 4.38 (s, 2H), 3.94 (s, 3H). **^{13}C NMR** (101 MHz, $CDCl_3$) δ 190.08, 153.18, 149.79, 136.04, 128.75, 128.22, 127.27, 127.20, 123.66, 112.13, 111.28, 70.87, 56.13, 30.40.

7-(2-(4-(benzyloxy)-3-methoxyphenyl)-2-oxoethoxy)-4-methyl-2H-chromen-2-one [3]

[2] (5.0 g, 0.0149 mol, 1 eq), 4-methylumbelliferone (2.62 g, 0.0149 mol, 1 eq) and anhydrous K_2CO_3 (3.78 g, 0.0273 mol, 1.8 eq) were dissolved in dry acetone (250 mL) and heated to reflux under argon atmosphere. The reaction was monitored by TLC (30:70 ACN/toluene) and stopped after 2.5h. Acetone was removed by reduced pressure. The concentrate was dissolved in brine (300 mL) and extracted with DCM (3x250 mL). The combined organic phases were dried over Na_2SO_4, filtered, and concentrated under reduced pressure. Crude product was purified by flash column chromatography using a solvent gradient DCM:EtOAc (v/v, 100:10 to 25:1). White crystals were afforded (4.48 g, 70% yield). **^{1}H NMR** (400 MHz, $CDCl_3$) δ 7.57 – 7.29 (m, 8H), 6.93 (d, J = 8.0 Hz, 2H), 6.77 (d, J = 2.5 Hz, 1H), 6.13 (s, 1H), 5.31 (s, 2H), 5.25 (s, 2H), 3.94 (s, 3H), 2.40 – 2.36 (m, 3H). **^{13}C NMR** (101 MHz, $CDCl_3$) δ 191.69, 161.08, 161.00, 155.08, 153.35, 152.42, 149.93, 136.00,128.76, 128.24, 127.52, 127.20, 125.75, 122.40, 114.25, 112.66, 112.33, 110.58,101.89, 70.89, 70.37, 56.14, 18.68.

7-((1-(4-(benzyloxy)-3-methoxyphenyl)-3-hydroxy-1-oxopropan-2-yl)oxy)-4-methyl-2H-chromen-2-one [4]

Solution of dry formaldehyde-EtOAc was prepared prior to reaction start: 10 mL formaldehyde (37%) washed and extracted with 3x50 mL EtOAc, then blown through with argon and kept in flask with activated molecular sieves to keep dry. [3] (2.0 g, 0.00465 mol, 1 eq) was dissolved in 70 mL of dry formaldehyde-EtOAc solution (0.04687 mol, 10 eq) and K_2CO_3 (0.075 g, 0.000511 mol, 0.11 eq) was added. Reaction heated to reflux until no visible precipitation. Reaction was carried out at room temperature under argon atmosphere and monitored by TLC (30:70 ACN/toluene). After 3h, 35 mL formaldehyde-EtOAc (0.02344 mol, 5 eq) was added. After additional 2h, K_2CO_3(0.075 g, 0.000511 mol, 0.11 eq) was added. After a total of 7h, the reaction was stopped by dilution with brine (150 mL) and extraction with DCM (3x150 mL). The combined organic phases were dried over Na_2SO_4, filtered, and concentrated under reduced pressure. Recrystallisation was performed using 70:30 toluene/EtOAc. Flash column chromatography was performed using a solvent gradient DCM:EtOAc (v/v, 100:10 to 25:1). Light pink crystals were afforded (1.047 g, 52% yield).**^{1}H NMR** (400 MHz, $CDCl_3$) δ 7.56 (d, J = 2.0 Hz, 1H), 7.51 – 7.32 (m, 7H), 6.95 – 6.88 (m, 2H), 6.71 (d, J = 2.5 Hz, 1H), 6.12 (d, J = 1.3 Hz, 1H), 5.64 (td, J = 6.4, 2.9 Hz, 1H), 5.26 (s, 2H), 4.23 – 4.09 (m, 2H), 3.92 (s, 3H), 2.36 (d, J = 1.3 Hz, 3H).**^{13}C NMR** (101 MHz, $CDCl_3$) δ 193.26, 161.02, 160.15, 155.00, 153.71, 152.36, 150.02, 135.93, 128.79, 128.27, 127.54, 127.20, 125.91, 123.10, 114.47, 112.80, 112.48, 112.27, 111.07, 102.37, 80.98, 70.95, 63.63, 56.11, 18.66.

Guaiacylglycerol-β-O-4-methylumbelliferone [GBMU] and α-O-(β-methylumbelliferyl)acetovanillone [$C\alpha_{ox}$GBMU]

[4] (0.602 g, 0.0013 mol, 1 eq) dissolved in EtOH (30 mL) was heated to reflux until dissolved. Pd/C (0.059 g, 10%) was added and a H_2 flow was kept constant during reaction. The reaction was monitored by TLC every 30 min (40:60 ACN/toluene. After 3h, the reaction was stopped, and the reaction mixture was filtered through celite and rinsed with DCM. DCM and EtOH were evaporated under reduced pressure. Crude was dissolved in EtOAc (100 mL) and washed with brine (100 mL). The aqueous phase was extracted with EtOAc (2x100 mL). The combined organic

phases were dried over Na_2SO_4, filtered, and concentrated under reduced pressure. UPLC-MS (ESI) and ^{1}H NMR spectra confirmed the presence of a mixture of GBMU and $C\alpha_{ox}$GBMU, which were subsequently separated and purified using preparative HPLC. Water was removed by freeze drying, yielding white crystals.

$C\alpha_{ox}$GBMU characterization: **^{1}H NMR** (400 MHz, DMSO-d_6) δ 7.73 (dd, J = 8.3, 2.0 Hz, 1H), 7.66 (d, J = 8.8 Hz, 1H), 7.52 (d, J = 2.0 Hz, 1H), 6.98 – 6.88 (m, 2H), 6.82 (d, J = 2.5 Hz, 1H), 6.19 (d, J = 1.4 Hz, 1H), 6.01 (dd, J = 5.6, 3.5 Hz, 1H), 3.93 (qd, J = 12.0, 4.4 Hz, 2H), 3.83 (s, 3H), 2.36 (d, J =1.2 Hz, 3H). **^{13}C NMR** (101 MHz, DMSO-d_6) δ 193.94, 161.10, 160.48, 154.91, 153.80, 153.08, 148.20,127.06, 126.88, 124.18, 115.58, 113.84, 113.10, 112.05, 111.75, 102.25, 81.31, 62.98, 56.12, 18.55. **UPLC-MS** (ESI) m/z found 370.8 $[M+H]^+$, calculated 371.11 $[C_{20}H_{19}O_7]^+$.

GBMU characterization: **^{1}H NMR** (400 MHz, DMSO-d_6) δ 7.58 (d, J = 8.7 Hz, 1H), 6.97 – 6.85 (m, 3H), 6.77 (dd, J = 8.1, 1.9 Hz, 1H), 6.67 (d, J = 8.1 Hz, 1H), 6.16 (d, J = 1.3 Hz, 1H), 4.67 (d, J = 5.8 Hz, 1H), 4.52 (td, J = 6.2, 3.3 Hz, 1H), 3.71 (s, 3H), 3.62 (dd, J = 11.9, 6.3 Hz, 1H), 2.36 (d, J = 1.2 Hz, 3H). **^{13}C NMR** (101 MHz, DMSO-d_6) δ 162.59, 160.69, 154.94, 153.85, 147.48, 146.05, 133.40, 126.60, 119.97, 115.19, 113.85, 113.34, 111.74, 111.37, 103.00, 83.97, 71.96, 60.91, 56.01, 18.56. **UPLC-MS** (ESI) m/z found 372.8 $[M+H]^+$, calculated 373.11 $[C_{20}H_{21}O_7]^+$.

Enzyme production

*Mt*PPO7 (Genbank accession number XP_003662515.1), *Mt*PPO-809 (XP_003659809.1), and *Tt*PPO (XP_003666010.1) from *Myceliophthora thermophila*, *Cg*PPO-473 (XP_001221473.1) and *Cg*PPO-266 (XP_001224266.1) from *Chaetomium globosum*, and *Pp*PPO-c2092 from *Parascedosporium putredinins* NO1 (sequence obtained from Oates et al.[4]) were heterologously expressed in *Pichia pastoris* X33 and purified as described previously.[5]

Tyrosinase (*Ab*Tyr) from *Agaricus bisporus* was purified from a commercial mushroom tyrosinase powder (Sigma Aldrich) as previously described.[5] Noteworthy, the purified *Ab*Tyr was previously shown to contain the isoforms *Ab*PPO3 and *Ab*PPO4.[6] *Ab*Tyr did not require copper saturation.

Copper saturation of recombinant fungal *o*-methoxyphenolases (*o*-MPs)

The purified fungal *o*-MPs were incubated with $CuSO_4$ at a 1:6 molar ratio (enzyme:$CuSO_4$) for 30 min at 4°C in 20 mM sodium acetate, pH 6.0. Excess free copper was removed from the samples with a PD-10 desalting column (Cytiva) equilibrated with 20 mM sodium acetate, pH 6.0.

*Mt*PPO7, *Mt*PPO-809 and *Cg*PPO-473 were, at a later stage, subjected to an optimized copper-saturation protocol. The enzymes were treated with EDTA at a 1:10 molar ratio (enzyme:EDTA) for 2 h at room temperature and overnight at 4°C in 20 mM sodium acetate, pH 6.0. The samples were desalted with a PD-10 desalting column (Cytiva) and then incubated with $CuSO_4$ at a 1:50 molar ratio (enzyme:$CuSO_4$) for 2 h at room temperature in 20 mM sodium acetate, pH 6.0. A desalting step with a PD-10 desalting column (Cytiva) was applied, and the eluted samples were buffer exchanged additionally three times with the same buffer (20 mM sodium acetate, pH 6.0) using a 10 kDa Vivaspin 6 Centrifugal Concentrator Polyethersulfone (Sartorius) at 4°C to ensure removal of excess free copper.

Enzyme assays on dimers

The phenolic dimers GBG, Cα_{ox}GBG, SBG, GBMU, or Cα_{ox}GBMU (0.2 mM) were incubated with purified *o*-MPs (2 or 5 µM, as specified in the figures legends) in the presence or absence of ascorbate (1 mM) or hydrogen peroxide (100 µM), in 20 mM sodium acetate, pH 6.0. Control reactions with $CuSO_4$ (4 or 10 µM, as specified in the figures legends), instead of enzyme, were performed under the same conditions. Reactions were performed in duplicate via incubation at 30°C during mixing at 1200 rpm in an Eppendorf ThermoMixer® for different periods, as indicated in the Figures. The assays were stopped by incubation at 95°C for 5 min in an Eppendorf ThermoMixer® and centrifuged (10,000 g, 5 min). Enzyme reaction products were then analyzed by RP-UHPLC-PDA-ESI-ITMS (system 1 or system 2, as specified in the figures legends), as detailed below.

The release of 4-methylumbelliferone (MU) from GBMU and Cα_{ox}GBMU was verified and quantified by using a calibration curve of an authentic MU standard.

Enzyme assays on monomeric lignin model compounds

Vanillyl alcohol, vanillin, acetovanillone, 3,4-dihydroxy-5-methoxybenzaldehyde, guaiacyl-propane-1,2-diol, or guaiacyl-propane-1,2-dione (0.2 mM) were incubated with *Mt*PPO7 (2 µM) in the presence or absence of ascorbic acid (1 mM) or hydrogen peroxide (100 µM), in 20 mM sodium acetate, pH 6.0. Control reactions with $CuSO_4$ (4 µM) instead of enzyme were performed. Assays were performed in duplicate and incubated for 150 min at 25 °C and 1200 rpm in an Eppendorf ThermoMixer®. Reactions were heat-inactivated at 95°C for 5 min, centrifuged (10,000 g, 5 min), and analyzed by RP-UHPLC-PDA-ESI-ITMS (system 2, described below).

2-Methoxy-1,4-hydroquinone (MHQ, 0.2 mM), 2-methoxy-1,4-benzoquinone (MQ, 0.2 mM) and 6-methoxybenzene-1,2,4-triol ($CP1_{red}$, 0.2 mM) were incubated with *Mt*PPO7 (2 µM) at 25 °C and 1200 rpm for 30 min and heat-inactivated at 95 °C for 5 min prior to RP-UHPLC-PDA-ESI-ITMS analysis (system 2, described below). In the case of MQ, enzyme reactions were also carried out in the presence of ascorbic acid (1 mM). In the case of $CP1_{red}$, superoxide dismutase (100 U/mL) and ascorbic acid (1 mM) were added to $CP1_{red}$ solution to mitigate its spontaneous oxidation to $CP1_{ox}$. For the three substrates, control reactions were also performed in the absence of *Mt*PPO7.

Enzyme assays in $H_2{}^{18}O$

GBG (1 mM) was mixed with *Mt*PPO7 (5 µM) in $H_2{}^{18}O$ buffered with 5 mM sodium phosphate pH 7.0 (final ^{18}O concentration of 88%) in a LC vial and incubated for 48 min at 25 °C. A 5 µL aliquot was injected directly for UHPLC-MS analysis (system 4, described below) without prior heat inactivation. As a control experiment, a $CP1_{ox}$ standard solution was diluted in either $H_2{}^{16}O$ or $H_2{}^{18}O$ (also buffered with 5 mM sodium phosphate, pH 7.0) to a final $CP1_{ox}$ concentration of *ca.* 0.3 mM and analyzed by the same UHPLC-MS method.

Cα_{ox}GBG (1 mM) was incubated with *Mt*PPO7 (5 µM) and ^{16}O-hydrogen peroxide (100 µM, to accelerate substrate conversion) in $H_2{}^{18}O$ (final ^{18}O concentration 86 % v/v) or Milli-Q $H_2{}^{16}O$. The reaction was incubated at 25 °C and 1200 rpm in an Eppendorf ThermoMixer® for 2 h and heat-inactivated at 95 °C for 5 min. An aliquot of the reaction was directly analyzed by UHPLC-MS (system 2, described below), while another aliquot was vacuum-dried at 60°C in an Eppendorf

Concentrator plus. The dried reaction performed in $H_2{}^{18}O$ was resuspended in $H_2{}^{16}O$, while the dried reaction performed in $H_2{}^{16}O$ was resuspended in $H_2{}^{18}O$ and analyzed by UHPLC-MS within 2 h after resuspension.

Effect of pH on GBG cleavage by *Mt*PPO7

GBG (0.2 mM) was incubated with *Mt*PPO7 (2 µM) in 20 mM sodium acetate (pH 4.0, 5.0, and 6.0), 20 mM sodium phosphate (pH 6.0, 7.0, and 8.0) or 20 mM Tris-HCl (pH 8.0 and 9.0). Reactions were performed in duplicate via incubation at 30°C during mixing at 1200 rpm in an Eppendorf ThermoMixer® for 30 min. The assays were stopped by incubation at 95°C for 5 min in an Eppendorf ThermoMixer® and centrifuged (10,000 g, 5 min). Reaction products were analyzed by RP-UHPLC-PDA-ESI-ITMS using system 2 (described below).

Quantification of monophenolase and diphenolase activities of *o*-MPs on vanillic acid and 3,4-dihydroxy-5-methoxybenzoic acid

The monophenolase and diphenolase activities of *Mt*PPO7 and *Cg*PPO-473 were measured on vanillic acid and 3,4-dihydroxy-5-methoxybenzoic acid, respectively, at different pH conditions. *Mt*PPO7 and *Cg*PPO-473 were subjected to the same optimized copper saturation protocol prior to this experiment. Activities were quantified by measuring substrate consumption over time using a short UHPLC-PDA-MS gradient (System 3 described below). Reactions were performed in sodium acetate (pH 4.0, 5.0 and 6.0), sodium phosphate (pH 6.0, 7.0 and 8.0) or Tris-HCl (pH 8.0 and 9.0).

Enzyme reactions (450 µL initial volume) were performed in HPLC vials and incubated in the autosampler of the UHPLC instrument with the temperature set at 25 °C. The reaction mixtures contained 1 mM substrate in a 10 mM reaction buffer and an appropriate dose of enzyme depending on the substrate analyzed, i.e. 1 µM *o*-MP for vanillic acid and 0.25 µM *o*-MP for 3,4-dihydroxy-5-methoxybenzoic acid. This ensured a linear substrate conversion over the assay time. When assessing the enzymatic activity on vanillic acid, the reaction mixture contained either 20 or 30 molar equivalents H_2O_2 for *Cg*PPO-473 and *Mt*PPO7, respectively. The reactions were initiated by the addition of substrate followed by thorough mixing. Aliquots (5 µL) from the same vial were injected into the UHPLC-PDA-MS instrument at intervals of 6 min, in a total of four time points. Substrates were identified using MS data and quantified by integrating UV peak absorption at 280 nm. Calibration curves for both substrates (0.01 – 1.5 mM) at each pH were constructed. Controls were made for both substrates at each pH comprising either 3,4-dihydroxy-5-methoxybenzoic acid or vanillic acid with 30 µM H_2O_2. All reactions were performed in triplicates. Activity was measured in units (U) and defines the amount of enzyme able to catalyze the conversion of 1 µmol of substrate per minute under the assay conditions.

Effect of ascorbic acid on diphenolase activity of *o*-MPs towards 3-methoxycatechol

3-Methoxycatechol (0.2 mM) was incubated with *Mt*PPO7 (0.5 µM) or *Mt*PPO-809 (0.5 µM) in the presence or absence of ascorbic acid (1 mM), in 20 mM sodium acetate pH 6.0. Reactions (150 µL volume) were conducted in a 96-well UV-transparent plate (Corning, Arizona, USA), and absorbance at 375 nm was continuously monitored at 25°C using a BioTek Epoch2 spectrophotometer (Agilent, California, USA). All reactions were performed in duplicate.

Catalytic rates were calculated during the linear phase of product formation. One unit (U) of enzymatic activity was determined as 1 ΔAbs_{375nm}/min. *Mt*PPO7 and *Mt*PPO-809 were subjected to the same optimized copper saturation protocol prior to this experiment.

Quantification of GBG conversion rates by *o*-MPs

GBG (2 mM) was incubated with *Mt*PPO7 (0.1 µM), *Mt*PPO-809 (0.1 µM) or *Cg*PPO-473 (0.5 µM) and H_2O_2 (50 µM) in a LC vial at 25 °C and 1200 rpm in an Eppendorf ThermoMixer®. Immediately after enzyme addition, aliquots were repeatedly injected directly for LC–UV analysis without prior heat inactivation. Injections were performed every 16 min using a short UHPLC gradient (system 4, described below). GBG consumption rates were quantified using a GBG calibration curve.

RP-UHPLC-PDA-ESI-MS analysis of enzyme assays

Enzyme assay products were subjected to reverse phase ultra-high performance liquid chromatography coupled to photodiode array detection, electron-spray ionization and mass spectrometry analysis (RP-UHPLC-PDA-ESI-MS) using one of the systems described below.

System 1: A Vanquish UHPLC system (Thermo Scientific, San Jose, CA, USA) was utilized. Injections of 5 µL samples were made onto an Acquity UPLC BEH C18 column (150 × 2.1 mm, 1.7 µm particle size) equipped with a VanGuard pre-column of identical material (5 × 2.1 mm, 1.7 µm particle size) (Waters, Milford, MA, USA). The flow rate was maintained at 400 µL/min, with the column temperature set to 45°C. The mobile phases consisted of water (A) and acetonitrile (B), both containing 0.1% (v/v) formic acid. The gradient elution profile was as follows: 0–1.5 min at 5% B (isocratic); 1.5–32 min linear increase from 5% to 35% B; 32–33 min linear ramp to 100% B; 33–38 min at 100% B (isocratic); 38–39 min linear decrease to 5% B; and 39–44 min at 5% B (isocratic). The photodiode array (PDA) detector was configured to monitor wavelengths between 200 and 700 nm.

Mass spectrometry was performed using a Thermo Scientific LTQ Velos Pro mass spectrometer, connected to the UHPLC system via a heated electrospray ionization (ESI) source. Nitrogen was used as the sheath and auxiliary gas throughout the analysis. Data acquisition was conducted in both positive and negative ionization modes, covering an m/z range of 120–2000. For MS2 data-dependent analysis, collision-induced dissociation (CID) was applied with a normalized collision energy of 35%. The system was calibrated before analysis using LTQ Tune Plus 2.7 (Thermo Scientific) with direct injection of a mixture containing the tetrapeptide MRFA (Met-Arg-Phe-Ala) and chlorogenic acid (Sigma-Aldrich) in positive and negative ionization modes. Key operational parameters included an ion transfer tube temperature of 300°C, a source heater temperature of 250°C, and a source voltage of ±3.5 kV. Data processing was carried out using Xcalibur 4.5 and FreeStyle 1.8 software (Thermo Scientific).

System 2: A Dionex UltiMate 3000 UPLC system (Thermo Scientific, San Jose, CA, USA) was employed. A Hypersil Gold Phenyl column (150 × 2.1 mm, 3 µm particle size; Thermo Fisher Scientific, Waltham, MA, USA) was used for 5 µL sample injections. The flow rate was set at 400 µL/min, and the column temperature was maintained at 40°C. Elution was conducted using a three-solvent system: 0.1% formic acid in water (eluent A), acetonitrile (eluent B), and water (eluent C). The gradient profile was as follows: 0-2 min, 10% A, 5% B, 85% C; 2-32 min, linear gradient to

10% A, 35% B, 55% C; 32-38 min, isocratic at 100% B; 38-45 min, isocratic at 10% A, 5% B, 85% C. The PDA detector recorded wavelengths in the range of 190–600 nm.

Mass spectrometry was carried out with an Amazon SL ion trap mass spectrometer (Bruker Daltonics, Bremen, Germany), coupled to the UHPLC system via a heated ESI source. Data were acquired in both positive and negative ultra-scan mode, with a detection range of *m/z* 100–2000 and a target mass of *m/z* 300. Automatic data-dependent MS2 events were triggered for the three most abundant precursor ions. Key settings included a capillary voltage of 4.5 kV, an end plate offset of 0.5 kV, a nebulizer pressure of 3.0 bar, a dry nitrogen flow rate of 12.0 L/min, and a dry gas temperature of 280°C. The resulting data were analyzed using Compass DataAnalysis 5.2 software (Bruker Daltonics, Bremen, Germany).

System 3: The same UPLC instrument and mass spectrometer described in System 2 were used. A Cortecs C18 column (150 mm x 2.1 mm; 1.6 µm, Thermo Fisher Scientific, Sunnyvale, CA, USA) was used at a flow rate of 500 µL/min and column temperature of 55°C. Elution was conducted using a two-solvent system: 0.1% formic acid in water (eluent A) and acetonitrile (eluent B). The gradient profile was as follows: 0-1.5 min, isocratic 90% A, 10% B; 1.5-2.0 min, linear gradient to 10% A, 90% B; 4.0-6.0 min, isocratic at 90% A, 10% B. All other instrument conditions were the same as described in System 2.

System 4: The same UPLC instrument described in System 2 was used but with a different elution gradient. Elution was conducted using a three-solvent system: 0.1% formic acid in water (eluent A), acetonitrile (eluent B), and water (eluent C). The gradient profile was as follows: 0 – 2 min, 10% A, 5% B, 85% C; 2 – 11 min, linear gradient to 10% A, 50% B, 40% C; 11.01 – 15.5 min, isocratic at 10% A, 5% B, 85% C.

Mass spectrometry was carried out with a Bruker compact QTOF mass spectrometer (Bruker Daltonics, Bremen, Germany), coupled to the UPLC system via a heated ESI source. Data were acquired in positive mode, with a detection range of *m/z* 50–1300. Key settings included a capillary voltage of 3.0 kV, an end plate offset of 0.5 kV, a nebulizer pressure of 3.5 bar, a dry nitrogen flow rate of 12.0 L/min, and a dry gas temperature of 250°C. The resulting data were analyzed using Compass DataAnalysis 5.2 software (Bruker Daltonics, Bremen, Germany).

Nuclear Magnetic Resonance (NMR) analysis of enzymatic reaction products

In reactions aimed at NMR analysis, 2 mM GBG (20 mg solubilized in a final volume of 31.2 mL 10 mM sodium phosphate, pH 6.0) was incubated with 1 µM *Mt*PPO7 in 50 mL conical tubes for 21 h at 25 °C and 200 rpm. 5 mM $C\alpha_{ox}$GBG (50 mg solubilized in a final volume of 31.4 mL 10 mM sodium phosphate, pH 6.0) was incubated under the same conditions with 1 µM *Mt*PPO7 and two additions of 40 µM H_2O_2 (at zero and 6 h, respectively). Control reactions under identical conditions but lacking *Mt*PPO7 were performed in parallel. The samples were inactivated at 95 °C for 10 min and freeze-dried.

The NMR characterization of the mixture of products generated from the oxidation of GBG and $C\alpha_{ox}$GBG by *Mt*PPO7 was performed on a Bruker Avance Neo 500 MHz spectrometer equipped with a proton-optimized triple resonance NMR 'inverse' TCI cryoprobe (CPTCl).

HSQC (Heteronuclear Single Quantum Coherence) spectra were recorded using standard pulse sequences with some modifications: JCH = 145 Hz, D1=5s, NS=8, P1=7.15us, TD (F2, F1)=512-128, O1P(F2,F1) = 6.3ppm, 125ppm, SW(F2,F1) = 13.1547ppm, 250ppm. The spectra were processed using the software Bruker TopSpin 4.2.0. The deuterated DMSO peak was

systematically used as reference (DMSO-d6 δH/δC 2.50/39.50). A contour level of 20 was applied to improve peak definition. For the analysis of the $C\alpha_{ox}$GBG prior to any enzymatic treatment, we had to increase the d6 delay to 100ms instead to 50ms to allow for a longer evolution time for long-range couplings.

COSY (Correlated Spectroscopy) spectra were recorded using standard pulse sequence (cosygpppqf) with 16 scans and a relaxation delay of 2 seconds. HMBC (Heteronuclear Multiple Bond Correlation)l spectra were recorded using standard pulse sequence (hmbcgplpndqf) with 32 scans, O1P(F2,F1) = 6.3ppm, 125ppm, SW(F2,F1) = 13.1547ppm, 250ppm, and a relaxation delay of 2.5 seconds.

^{31}P NMR:

Quantitative ^{31}P NMR spectroscopy was performed following the procedure published by Meng et al. 2019 working in a nitrogen atmosphere. Briefly, after drying the samples overnight at 45 °C in a vacuum oven, approximately 30 mg of material were inserted in a glass vial equipped with a magnetic stir bar and closed with a polytetrafluoroethylene (PTFE) septum sealed cap. To this vial, we added 0.1 mL of a solution of deuterated pyridine and $CDCl_3$ (1.6 : 1, v/v) containing chromium(III) acetylacetonate solution (≈5.0 mg mL^{-1}) and NHND as an internal standard (≈18.0 mg mL^{-1}) with a gas-tight syringe. To the vial were then added 0.5mL of a solution of deuterated pyridine and $CDCl_3$ (1.6 : 1, v/v), and the material was allowed to fully solubilize while stirring. After complete solubilization, 0.1mL of 2-chloro-4,4,5,5-tetramethyl-1,3-2-dioxaphospholane (TMDP) was added dropwise to the solution. The mixture was stirred for an additional hour to achieve complete derivatization and then transferred to an NMR tube previously dried at 120 °C and equipped with a rubber septum. The samples were analyzed within 3h of their preparation. The ^{31}P NMR spectra were recorded on a Bruker Avance 600 MHz spectrometer equipped with a 5 mm BBO cryoprobe. The experimental parameters used for the spectra acquisition were: pulse program=inverse gated decoupling pulse (zgig), SW=100 ppm, O1P=140 ppm, AQ=0.8 s, D1=10 s, NS=128.

Quantification of $CP1_{ox}$ in the standard solution and in enzymatic reactions

^{1}H NMR was used to selectively quantify $CP1_{ox}$ in the standard solution generated from the spontaneous auto-oxidation of $CP1_{red}$ in DMSO-d_6. Dimethyl sulfone (6H, 2.99 ppm) was used as an internal standard. For analysis, 400 µL of dimethyl sulfone (43 mg/L in DMSO-d_6) was mixed with 100 µL of $CP1_{ox}$ solution in an NMR tube. ^{1}H NMR spectra were recorded using a relaxation delay (D1) of 30 seconds for quantitative analysis. To avoid interference by the presence of other auto-oxidation products (such as $CP1_{ox}$ dimer, see Figure S14), the ring C-H signals of $CP1_{ox}$ were used for quantifying its concentration relative to the internal standard, since these signals are most selective to free $CP1_{ox}$. We conservatively estimated the concentration of $CP1_{ox}$ at 0.40 mg/mL in DMSO-d_6 (assuming a DMSO-d_6 density of 1.19 g/mL).

On the same day, the same sample was used to make a calibration curve in LC-PDA-MS (Figure S14). This allowed the quantification of $CP1_{ox}$ released by enzyme treatment of GBG.

SI Figures

	Pp PPO-c2092	*Mt* PPO7	*Mt* PPO-809	*Cg* PPO-266	*Cg* PPO-473	*Tt* PPO	*Ab* PPO3
Pp PPO-c2092	100	60.44	63.11	63.7	59.85	60.59	15.26
Mt PPO7		100	63.56	56	86.37	56.89	12.15
Mt PPO-809			100	61.48	64.44	59.7	17.19
Cg PPO-266				100	54.81	55.85	16
Cg PPO-473					100	57.83	12.8
Tt PPO						100	11.52
Ab PPO3							100

Figure S1. Amino acid sequence identity (%) among the enzymes used in this study, based on Clustal Omega pair-wise alignment.

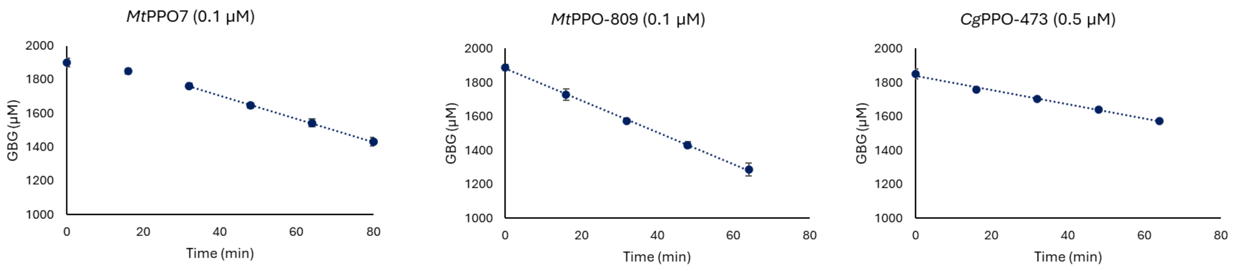

Enzyme	GBG conversion rate	GBG apparent turnover
	µM GBG · min^{-1}	µM GBG · µM Enzyme^{-1} · s^{-1}
*Mt*PPO7 (0.1 µM)	6.72	1.12
*Mt*PPO-809 (0.1 µM)	9.48	1.58
*Cg*PPO-473 (0.5 µM)	3.86	0.13

Figure S2. GBG consumption by *Mt*PPO7 (0.1 µM), *Mt*PPO-809 (0.1 µM) and *Cg*PPO-473 (0.5 µM). The reactions were carried out at 25 °C and 1200 rpm with 2 mM GBG and 50 µM H_2O_2. In the case of *Mt*PPO7 reaction, a lag phase was observed, and therefore the linear substrate consumption rate was measured only after the third time point (32 min reaction). Including the entire time frame decreases the overall *Mt*PPO7 rate by approximately 10%. The GBG conversion rates and turnover numbers are shown in the table.

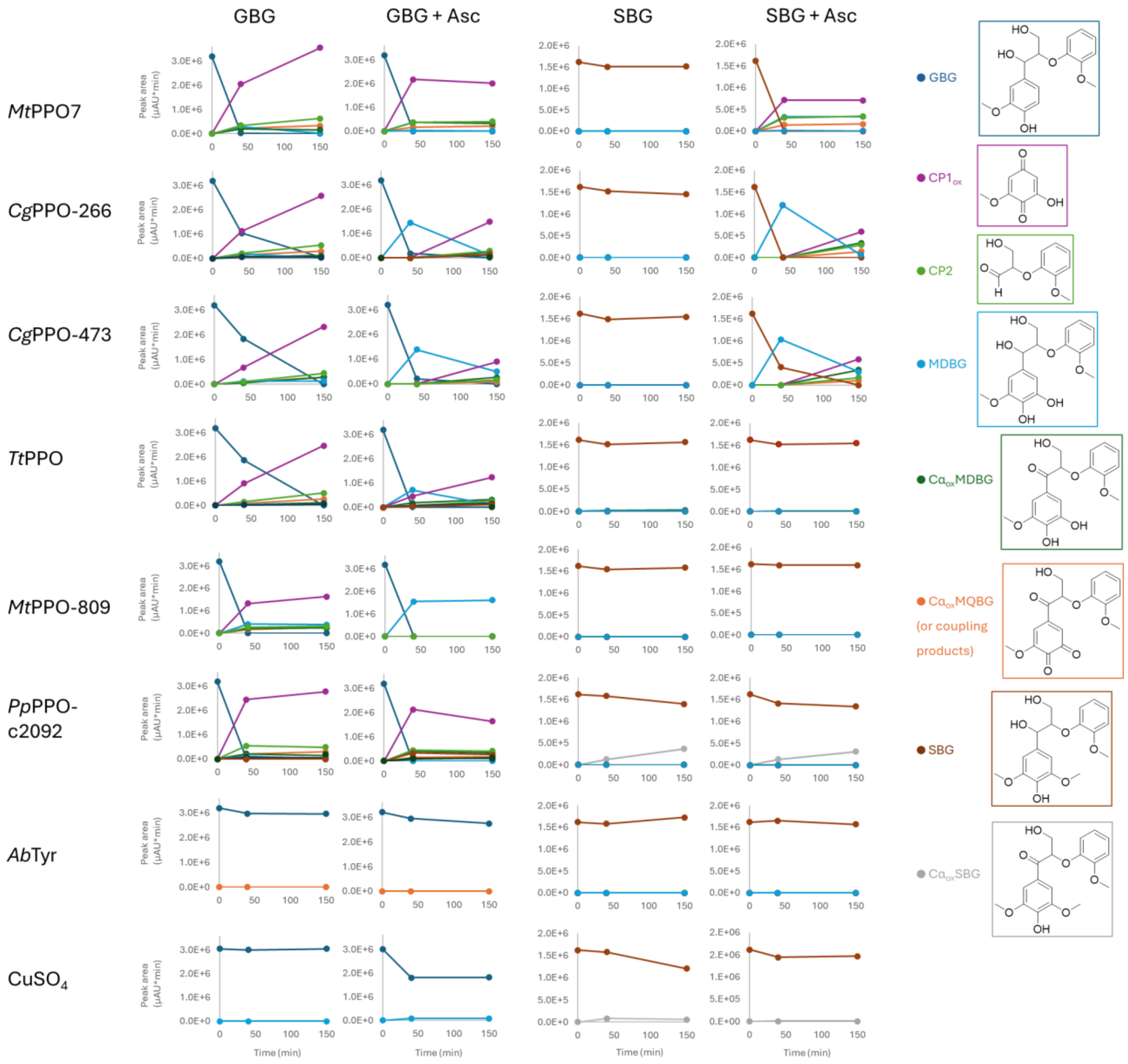

Figure S3. Time-course plots of GBG and SBG (0.2 mM) conversion by *o*-MPs (5 µM) in the absence or presence of ascorbic acid (1 mM). The relative quantification of the substrates and the main products was based on LC peak areas at 280 nm.

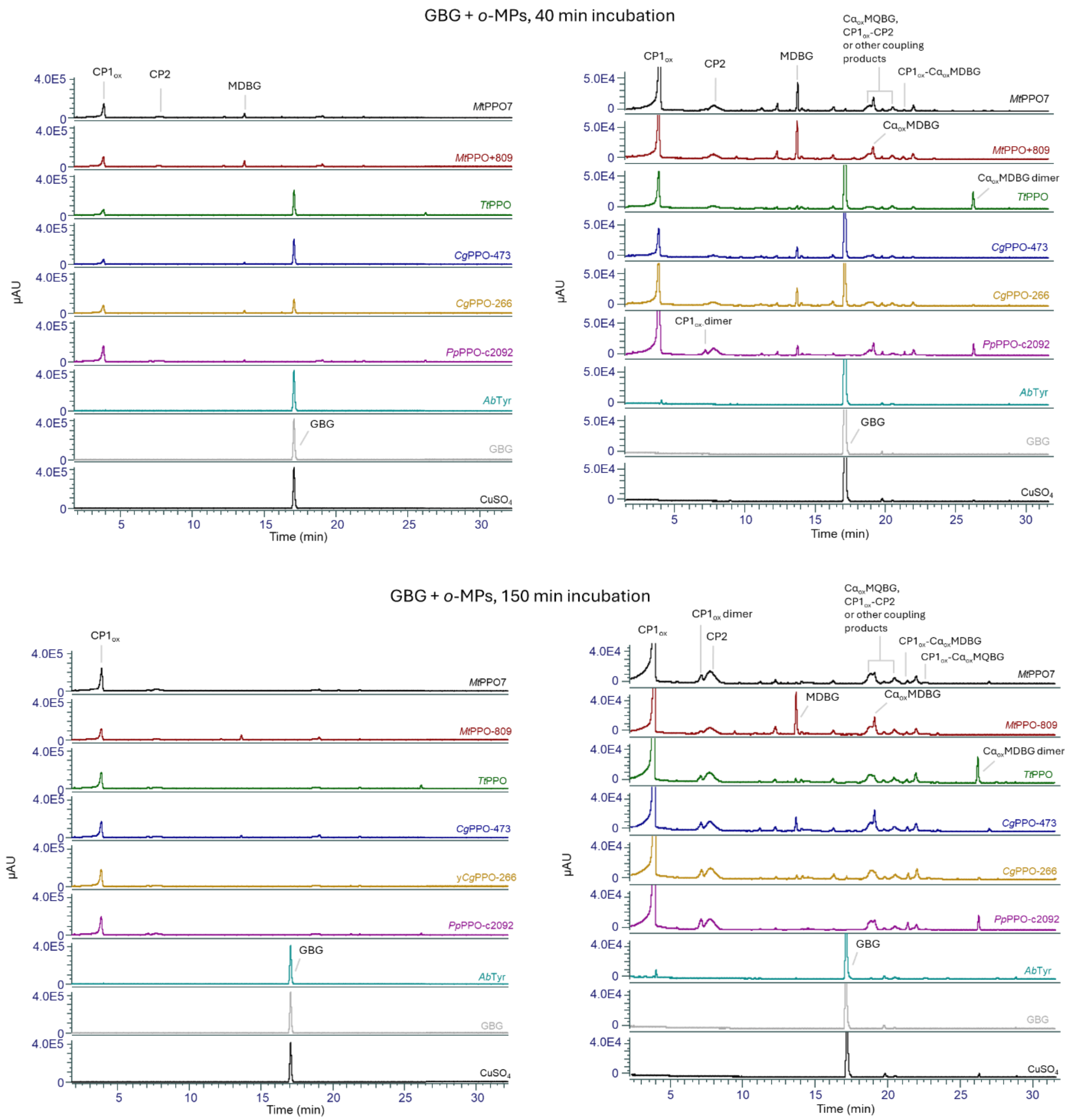

Figure S4. RP-UHPLC-PDA-ESI-MS chromatograms of the reaction products from *o*-MP-catalyzed (5 µM) oxidation of guaiacylglycerol-β-guaiacyl ether (GBG, 0.2 mM) after 40 min (upper panel) and 150 min (lower panel) of incubation. The chromatograms on the right are shown with a magnified Y-axis for better visualization of low-intensity signals, compared to those on the left. Control reactions with 10 µM $CuSO_4$, instead of enzyme, are also included. The analysis was performed using System 1 described in Materials and Methods.

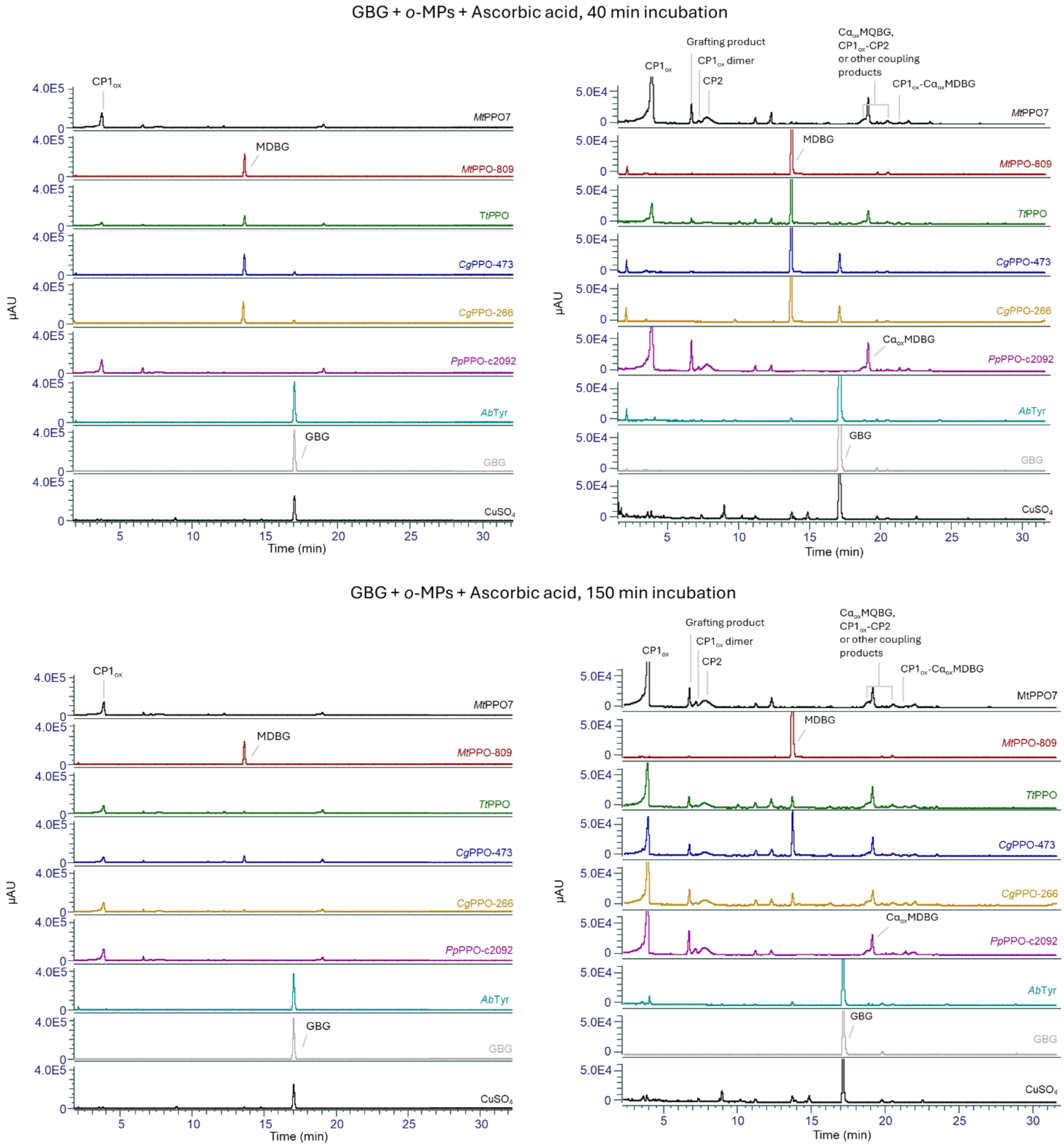

Figure S5. RP-UHPLC-PDA-ESI-MS chromatograms of the reaction products from *o*-MP-catalyzed (5 µM) oxidation of guaiacylglycerol-β-guaiacyl ether (GBG, 0.2 mM) in the presence of ascorbic acid (1 mM) after 40 min (upper panel) and 150 min (lower panel) of incubation. The chromatograms on the right are shown with a magnified Y-axis for better visualization of low-intensity signals, compared to those on the left. Control reactions with 10 µM $CuSO_4$, instead of enzyme, are also included. The analysis was performed using System 1 described in Materials and Methods.

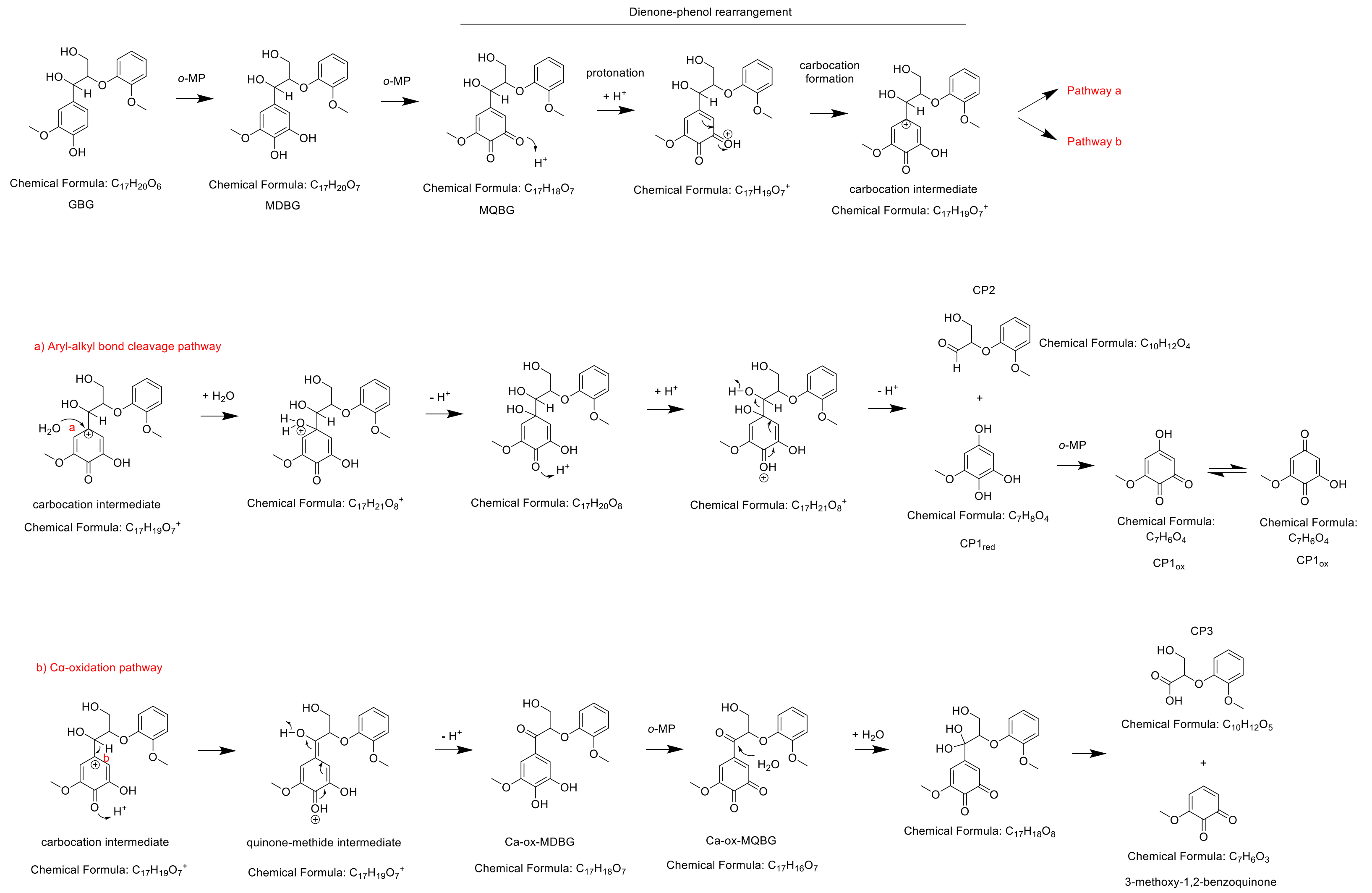

Figure S6. Detailed proposed reaction pathways initiated by activity of fungal *o*-MPs on guaiacylglycerol-β-guaiacyl ether (GBG).

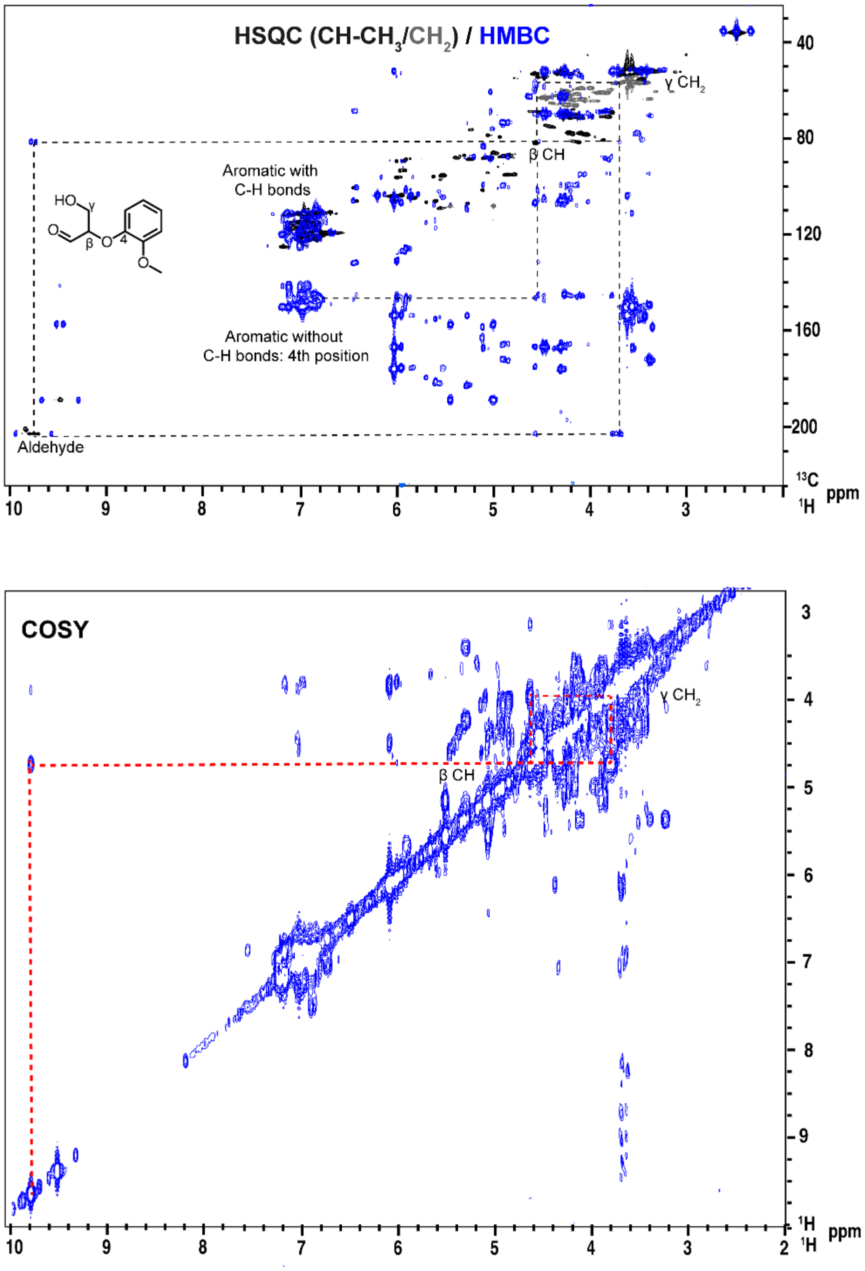

Figure S7. 2D ^{1}H–^{13}C HSQC and HMBC spectra (upper panel) and COSY spectrum (lower panel) of the pool of products of *Mt*PPO7 activity on GBG, with emphasis on the signals corresponding to the cleavage product <u>CP2</u> (structure shown in the upper panel).

The structure of the CP2 product was elucidated using a combination of HSQC, HMBC, and COSY NMR spectroscopy. The HSQC spectrum confirmed direct one-bond correlations between protons and their attached carbon atoms, while the HMBC spectrum provided long-range heteronuclear couplings that were instrumental in mapping out the carbon framework.

First, we identified the aldehyde peak (^{1}H: 9.75ppm, ^{13}C: 201ppm), which showed a direct coupling to an sp^3 carbon in the HMBC spectrum. Additional correlations to the same sp^3 carbon supported the connectivity and substitution pattern of the molecule. This β C–H signal was coupled to both a γ-CH_2 group and to an aromatic carbon lacking directly bonded protons (i.e., the fourth position on the aromatic ring). These assignments were further validated by COSY data, which revealed proton-proton couplings consistent with the proposed structure—most notably between the aldehyde and β C–H, and between the β C–H and γ C-H_2. Together with LC-MS data, these spectral features provide robust evidence for the identity and configuration of the aldehyde product.

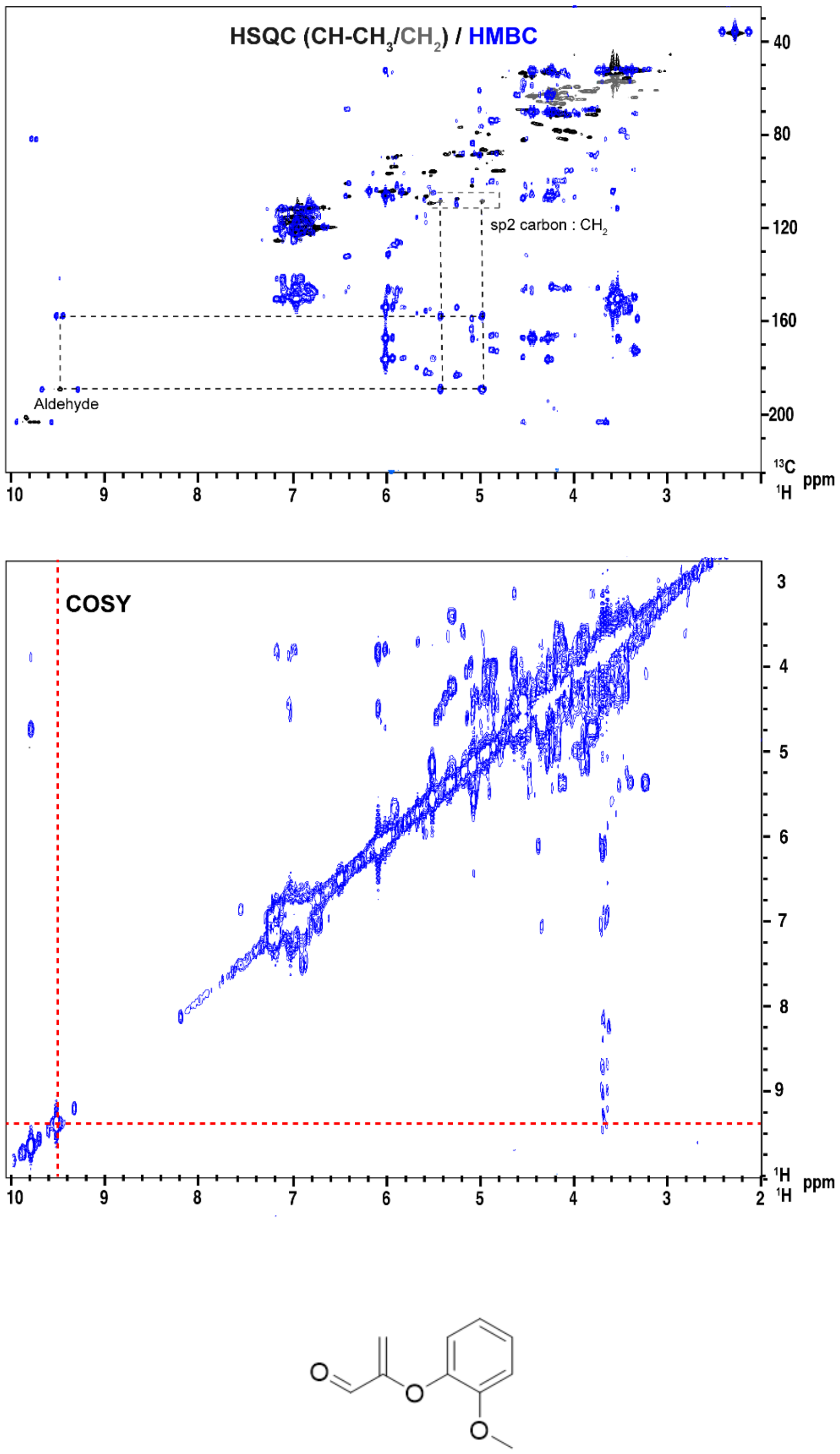

Figure S8. 2D ^{1}H–^{13}C HSQC and HMBC spectra (upper panel) and COSY spectrum (lower panel) of the pool of products of *Mt*PPO7 activity on GBG, with emphasis on the signals corresponding

to a Cα-aldehyde group adjacent to an alkene, potentially corresponding to cleavage product CP2^{alk} (hypothetical structure shown in the bottom).

The NMR analysis of the second aldehyde product reveals key structural insights through the HMBC spectrum, which shows a long-range correlation between the aldehyde proton (δ ≈ 9.5 ppm) and an sp^2 CH_2 group (δ ≈ 110 ppm), indicating proximity to an alkene moiety. This interaction suggests that the aldehyde group is conjugated with, or spatially close to, an unsaturated carbon center. Notably, the COSY spectrum shows no cross-peaks involving the aldehyde proton, confirming its isolation from other proton-rich environments and supporting its position at the terminus of the molecule. Together, these data support a structural motif in which the aldehyde is adjacent to an alkene, consistent with a conjugated or vinylic system.

(A) $CP1_{red}$: **^{1}H NMR** (400 MHz, DMSO)

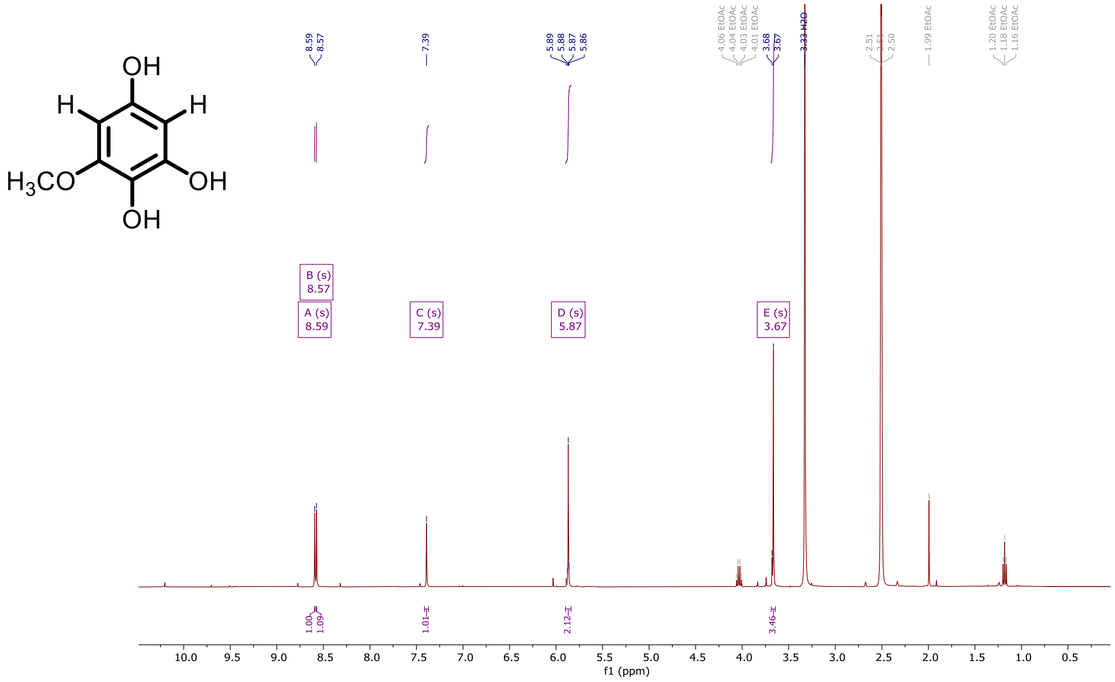

(B) $CP1_{red}$: **^{13}C NMR** (101 MHz, DMSO)

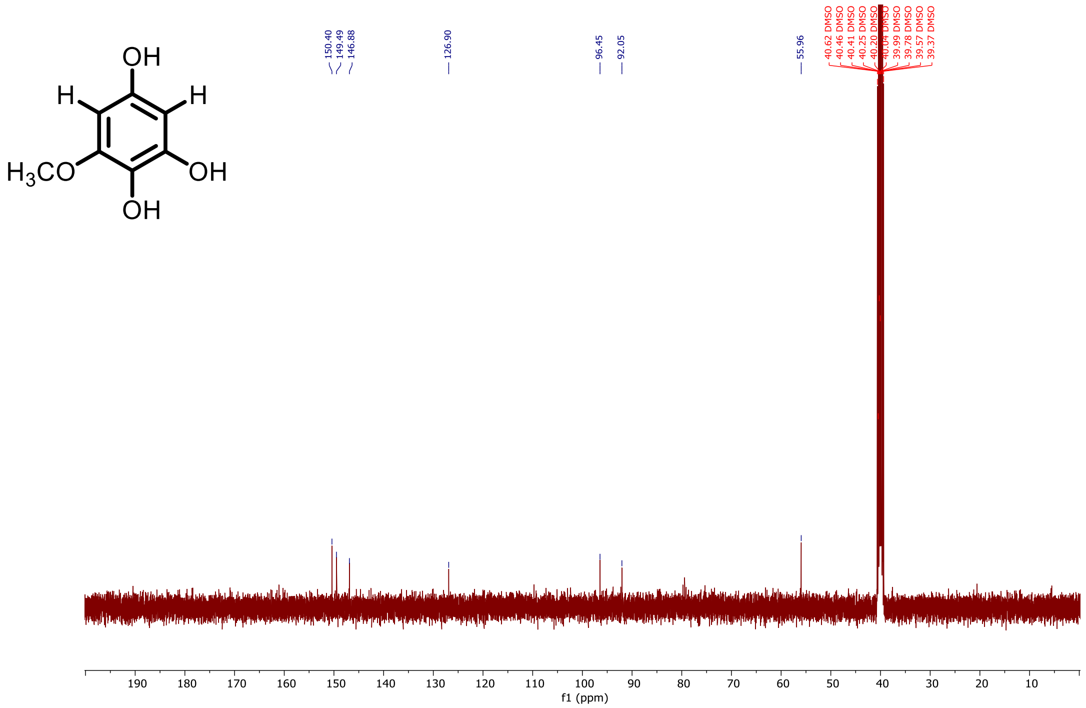

Figure S9. (continues below)

(C) CP1$_{red}$: **^{13}C DEPT 135 NMR** (101 MHz, DMSO)

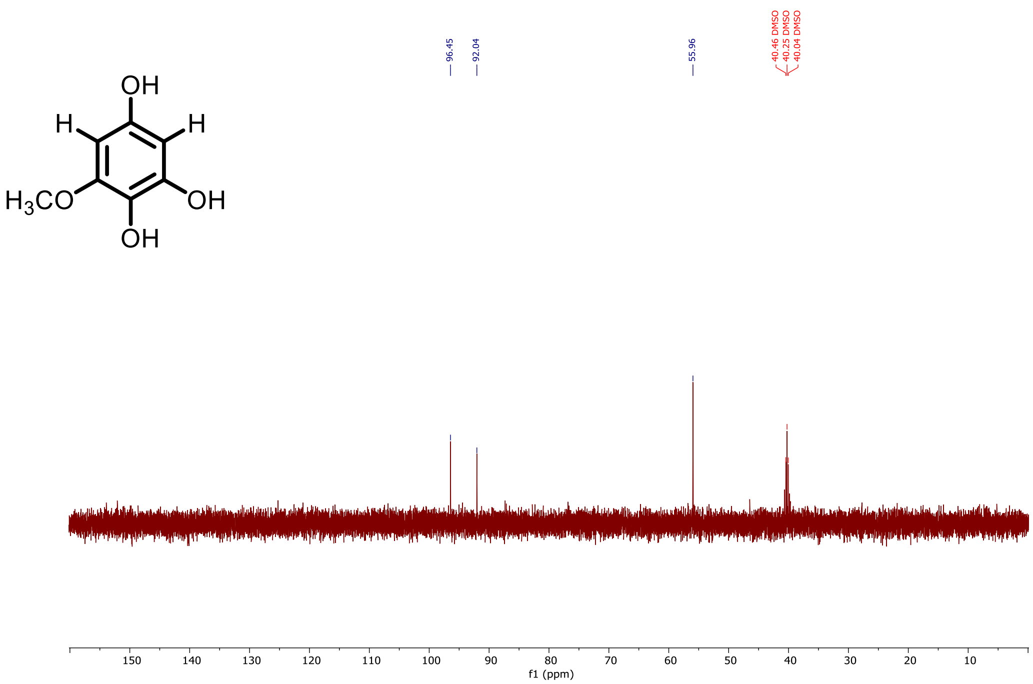

(D) CP1$_{red}$: **2D ^{1}H - ^{13}C HSQC NMR** (400 MHz / 101 MHz, DMSO)

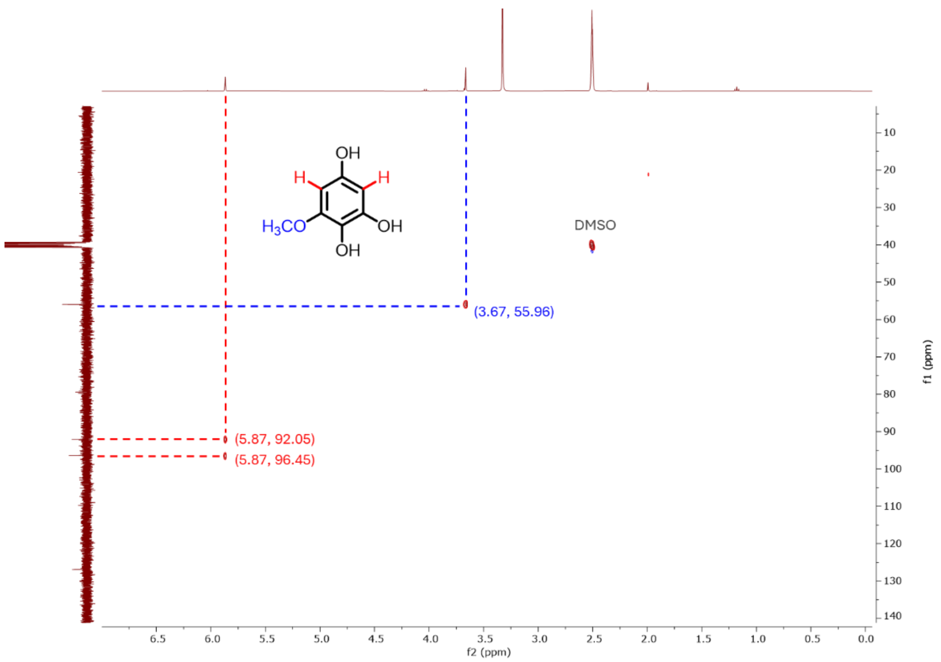

Figure S9. Assignment of CP1$_{red}$ by ^{1}H NMR (A), ^{13}C NMR (B), ^{13}C DEPT 135 NMR (C) and 2D ^{1}H - ^{13}C HSQC NMR (D).

(A) $CP1_{red}$ auto-oxidation after 1 day: **^{1}H NMR** (400 MHz, DMSO)

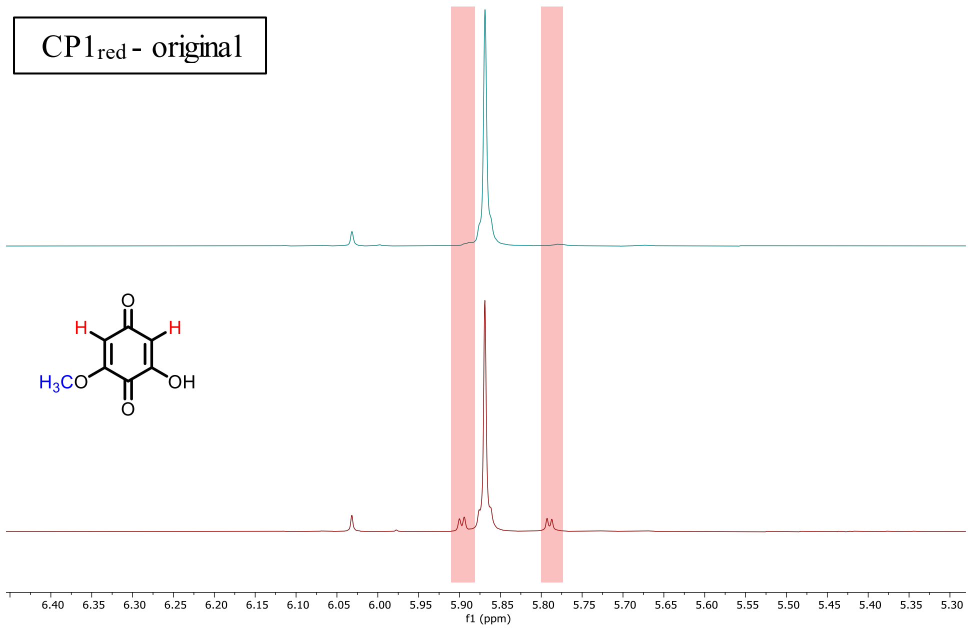

(B) $CP1_{red}$ auto-oxidation after 2 weeks: **^{1}H NMR** (400 MHz, DMSO)

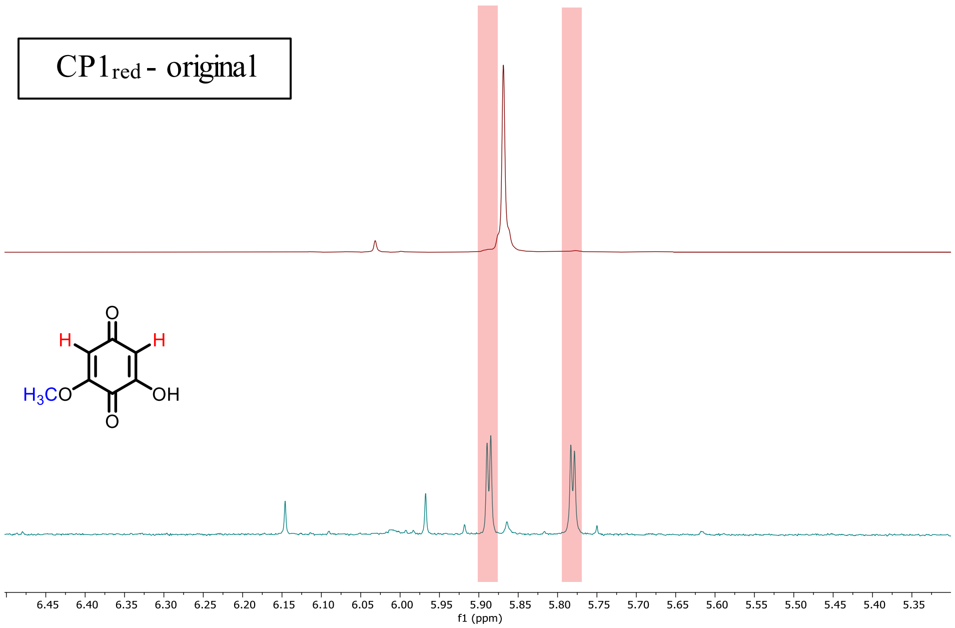

Figure S10. (continues below)

(C) $CP1_{ox}$: ^{1}**H NMR** (500 MHz, DMSO)

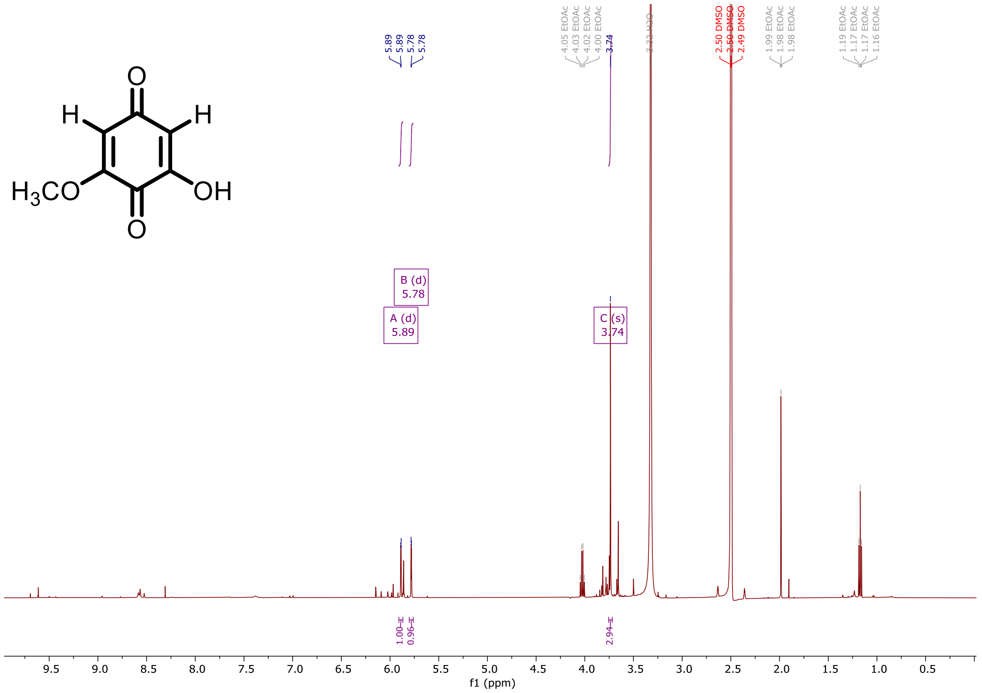

(D) $CP1_{ox}$: ^{13}**C NMR** (126 MHz, DMSO)

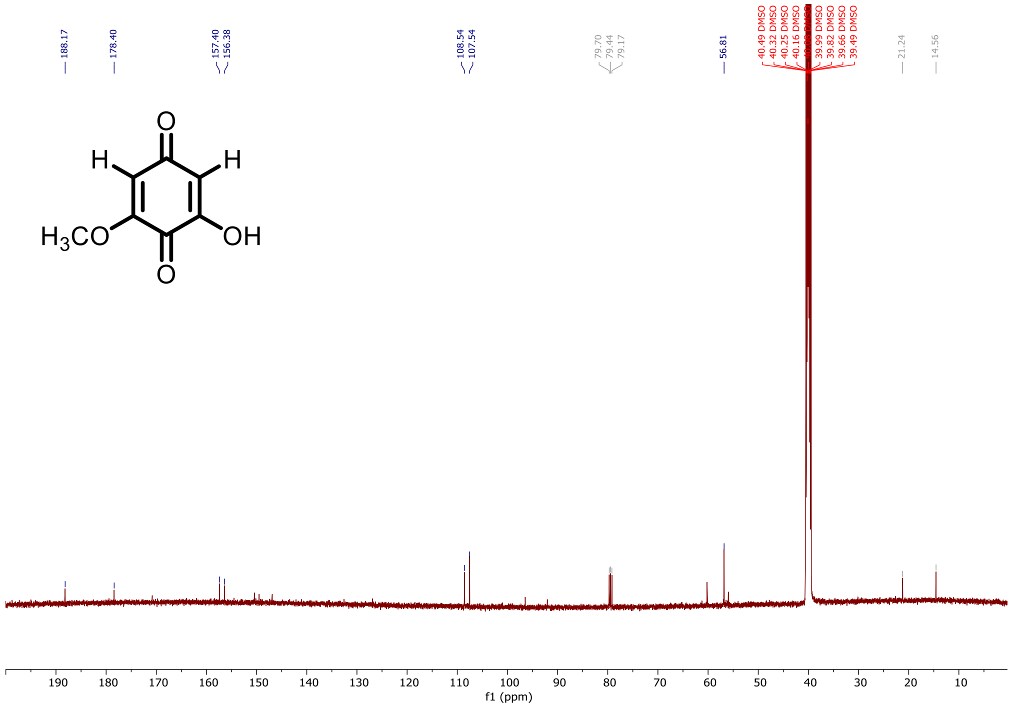

Figure S10. (continues below)

(E) CP1$_{ox}$: **^{13}C DEPT 135 NMR** (126 MHz, DMSO)

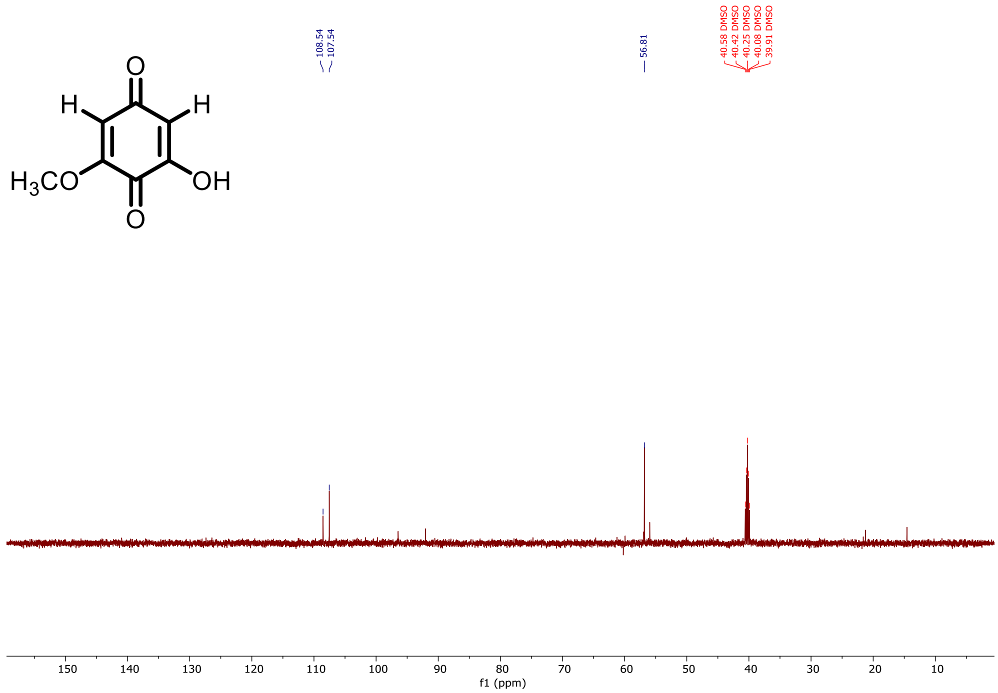

(F) CP1$_{ox}$: **2D ^{1}H - ^{13}C HSQC NMR** (500 MHz / 126 MHz, DMSO)

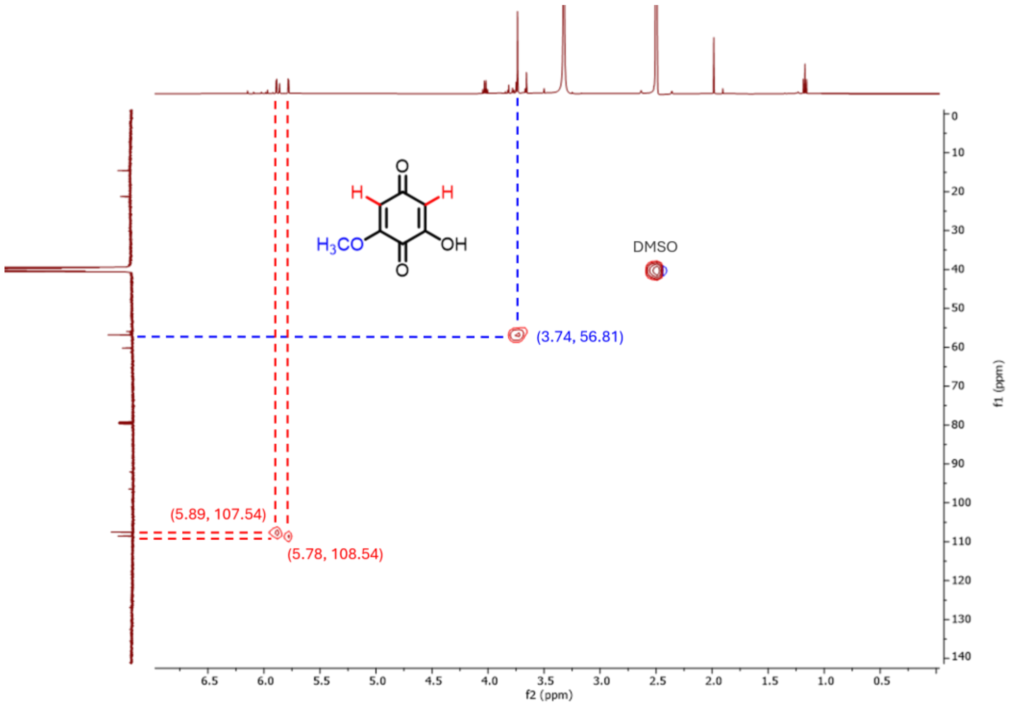

Figure S10. (continues below)

(G) CP1$_{ox}$: **2D ^{1}H - ^{13}C HMBC NMR** (500 MHz / 126 MHz, DMSO)

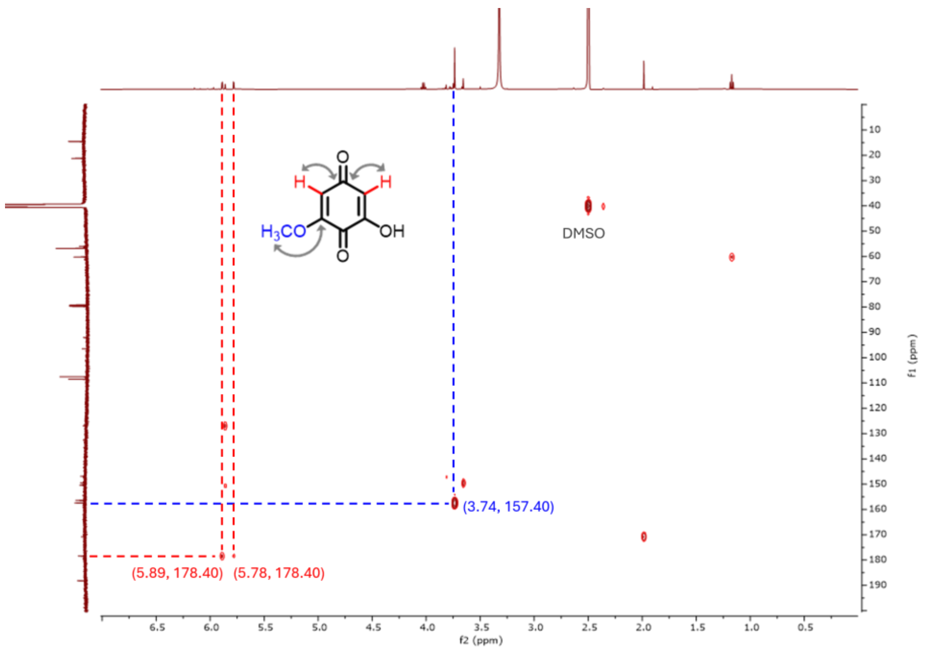

(H) CP1$_{ox}$ + Internal Standard (Dimethyl Sulfone): **^{1}H NMR** (500 MHz, DMSO)

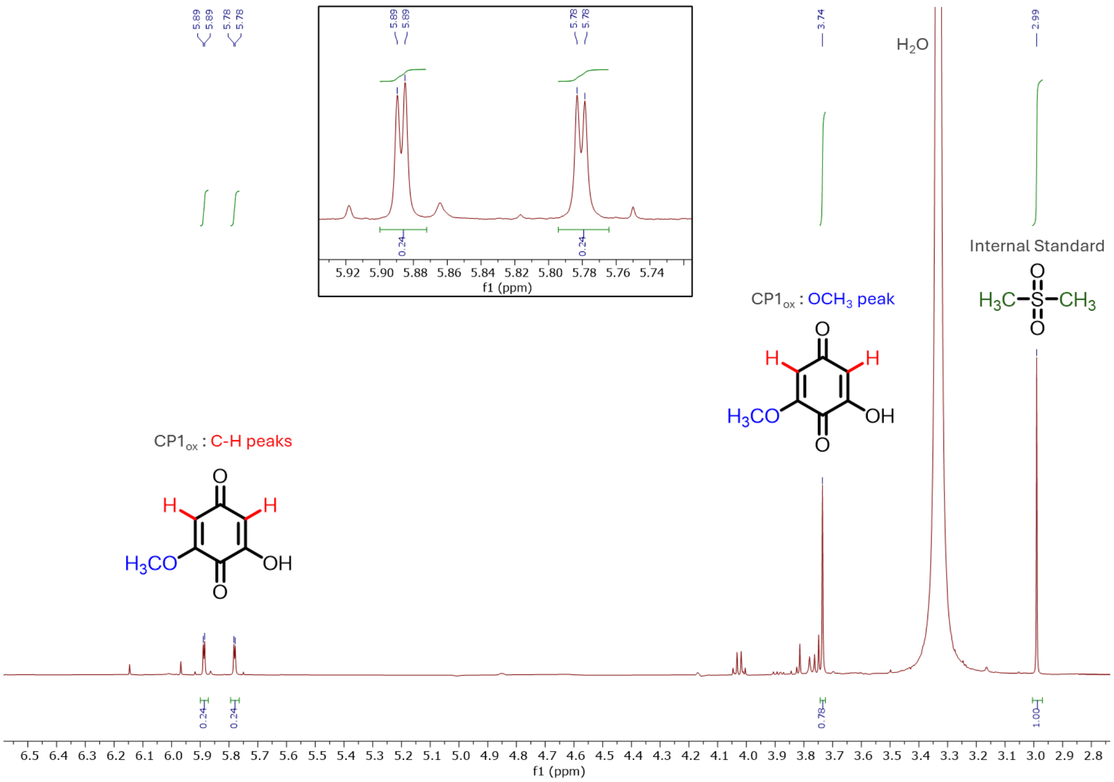

Figure S10. Assignment of CP1$_{ox}$ by ^{1}H NMR (A, B and C), ^{13}C NMR (D), ^{13}C DEPT 135

NMR (E), 2D ^{1}H - ^{13}C HSQC NMR (F), and 2D ^{1}H - ^{13}C HMBC NMR (G). The ^{1}H NMR spectra used for $CP1_{ox}$ quantification with an internal standard is shown in panel (H).

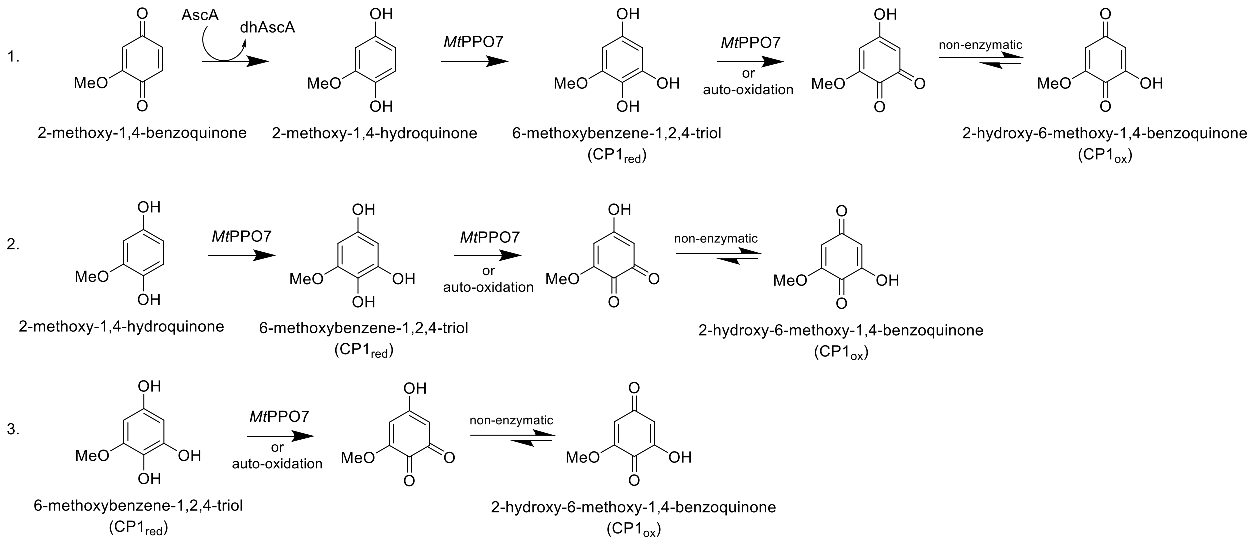

Figure S11. Independent enzymatic routes for the production of $CP1_{ox}$ from three different substrates, namely 2-methoxy-1,4-benzoquinone (reaction 1), 2-methoxy-1,4-hydroquinone (reaction 2), and 6-methoxybenzene-1,2,4-triol ($CP1_{red}$) (reaction 3).

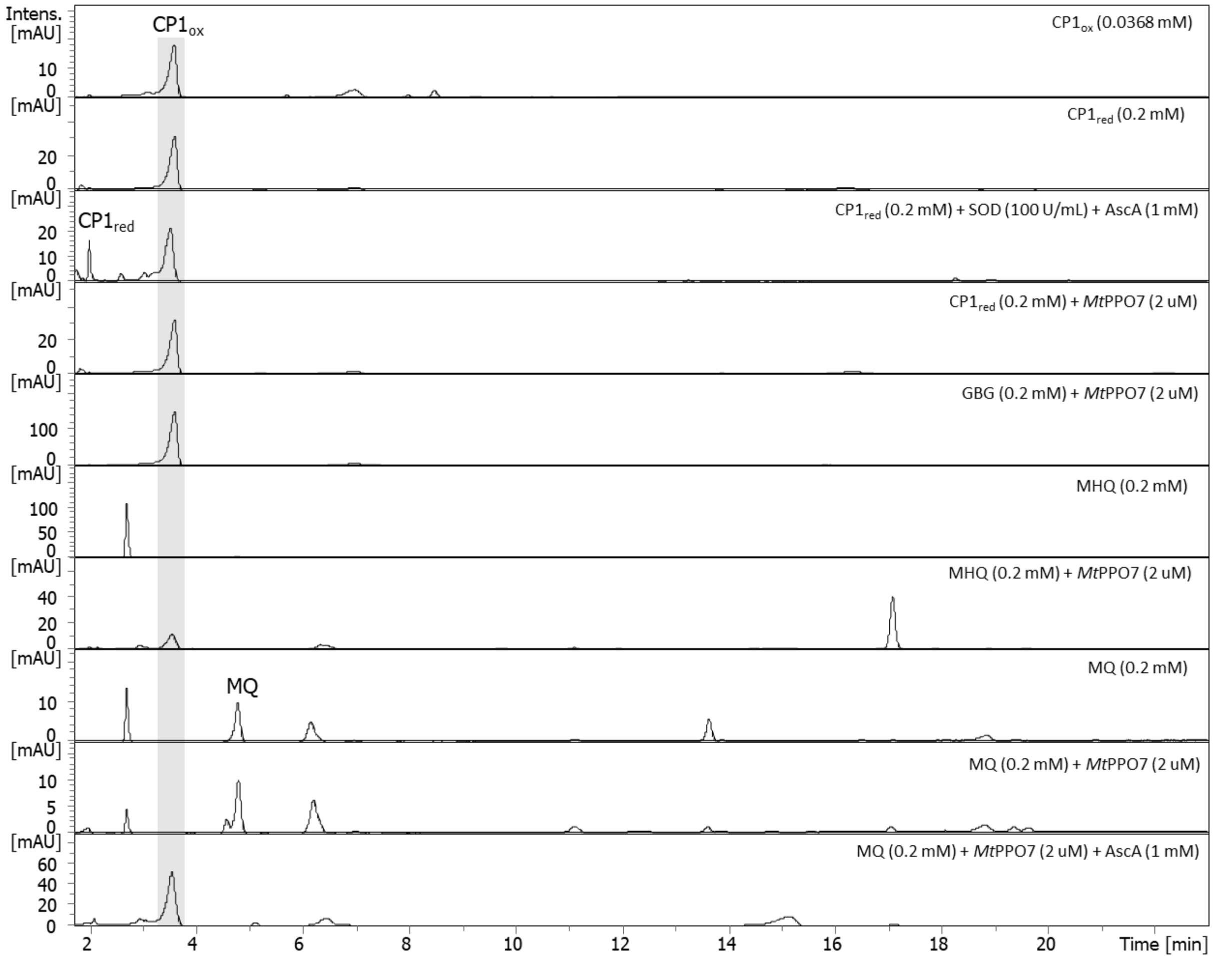

Figure S12. Verification of enzymatic production of $CP1_{ox}$ from four different substrates, namely GBG, $CP1_{red}$ (6-methoxybenzene-1,2,4-triol), 2-methoxy-1,4-hydroquinone (MHQ) and 2-methoxy-1,4-benzoquinone (MQ). The traces correspond to LC-UV chromatograms (absorbance at 280 nm) of the reaction products of *Mt*PPO7 activity on those substrates. The figure includes the chromatogram of $CP1_{ox}$ standard, as well as control reactions without *Mt*PPO7. Notes: $CP1_{red}$, when dissolved in aqueous buffer, rapidly oxidizes into $CP1_{ox}$ spontaneously. The addition of superoxide dismutase (SOD, 100 U/mL) and ascorbic acid (AscA, 1 mM) to $CP1_{red}$ solution partially prevents its oxidation.

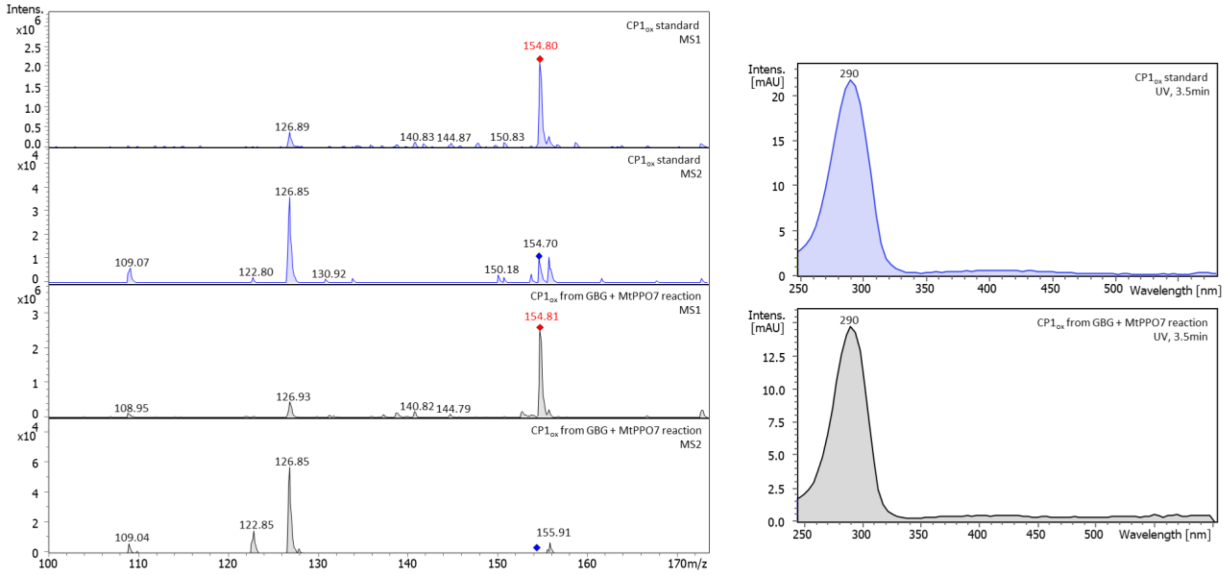

Figure S13. MS1 and MS2 spectra (left panel) and UV-vis spectra (right panel) of $CP1_{ox}$ authentic standard and of that produced from *Mt*PPO7 activity on GBG.

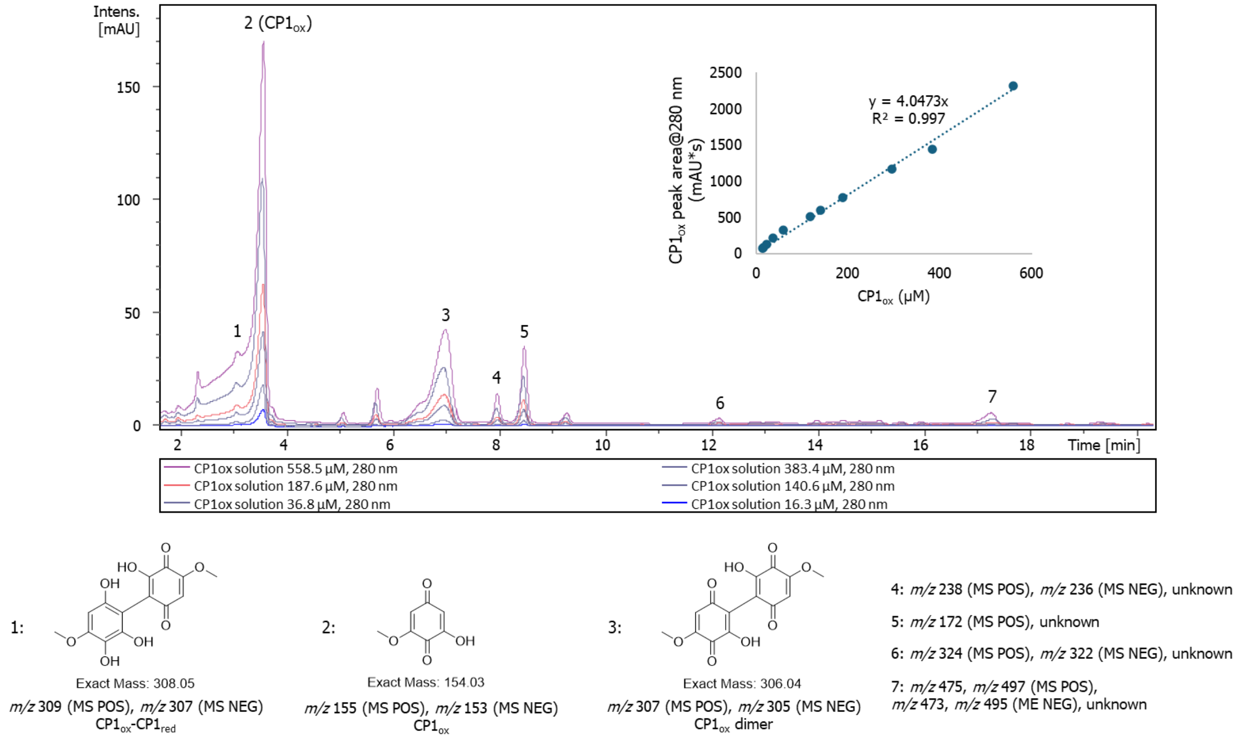

Figure S14. LC–UV chromatograms (λ 280 nm) of the $CP1_{ox}$ synthetic standard at varying concentrations. The corresponding calibration curve is shown in the inset. Major peaks are tentatively annotated in the lower panel.

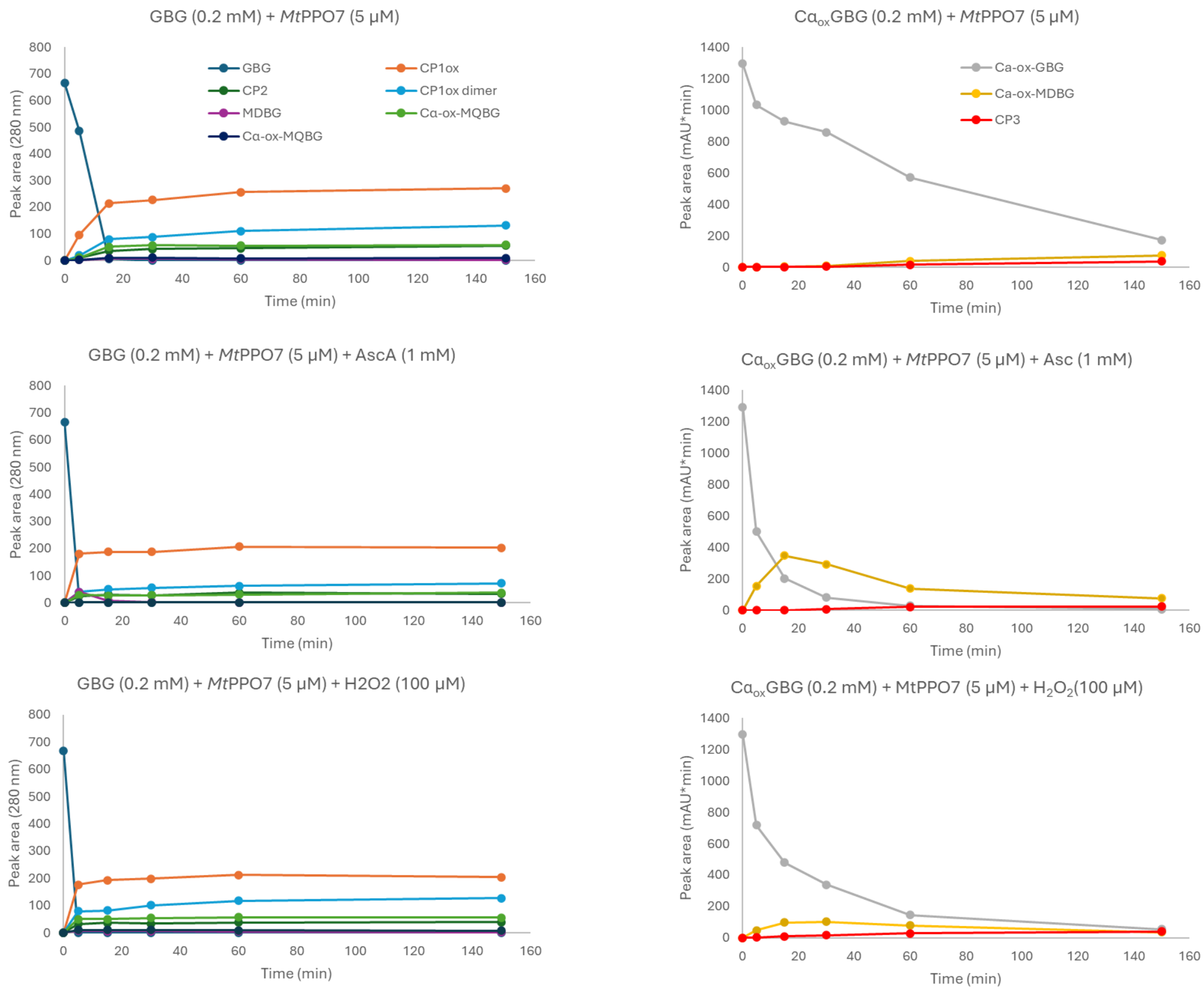

Figure S15. Time-course plots of *Mt*PPO7 (5 µM) activity on GBG and Cα_{ox}GBG (0.2 mM) in the absence or presence of ascorbic acid (1 mM) or hydrogen peroxide (100 µM). The relative quantification of the substrates and the main products was based on LC-PDA-MS peak areas at 280 nm.

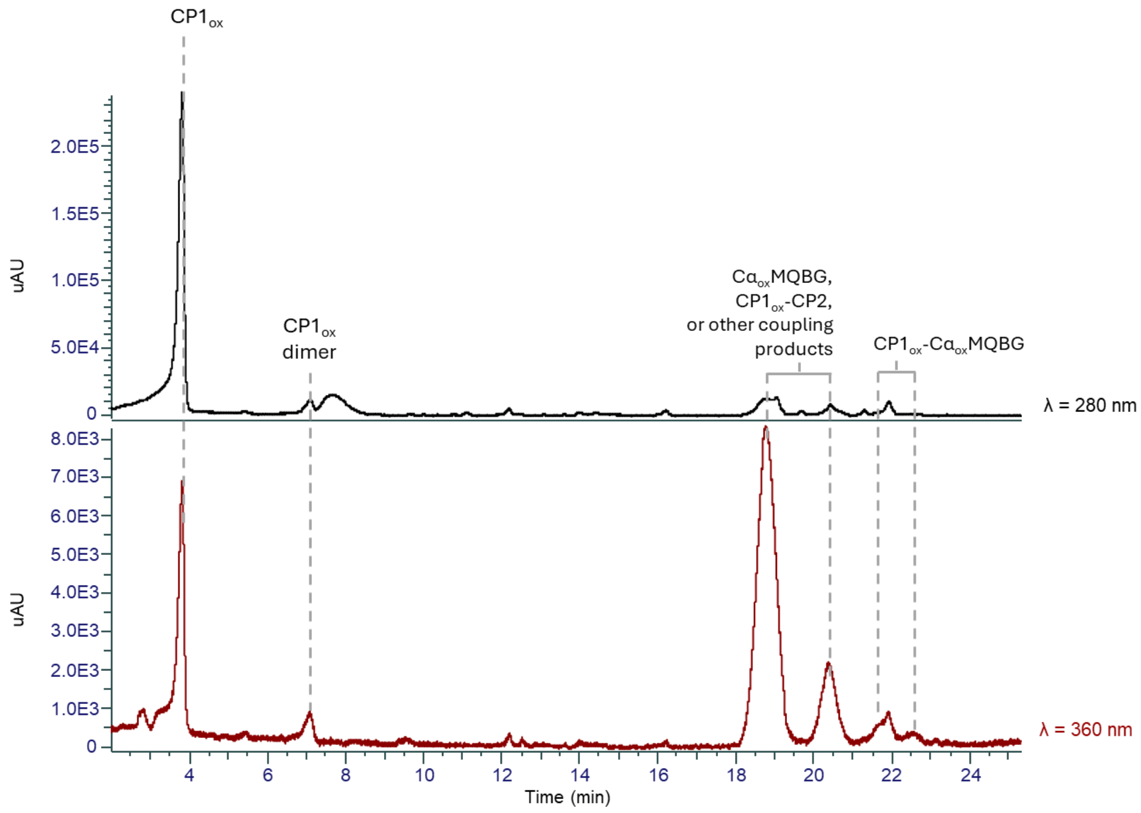

Figure S16. RP-UHPLC-PDA-ESI-MS chromatograms of the products of *Mt*PPO7 (5 μM) reaction on GBG (0.2 mM). Traces relative to absorbance at λ=280 nm and λ=360 nm are shown. Chromatograms were obtained with system 1, as described in Materials and Methods. The Y-axis of the chromatograms are on different scales.

The absorption at 360 nm, characteristic of quinone-containing species, observed for peaks at retention times of 18.8 and 20.4 min supports their annotation as $C\alpha_{ox}$MQBG. LC-MS peak assignment, however, is challenging due to multiple structural isomers and isobaric masses contributing to the MS signals, and peaks at 360 nm may also arise from e.g. coupling products incorporating the cleavage product $CP1_{ox}$ (please check hypothetical structures in Figure S17).

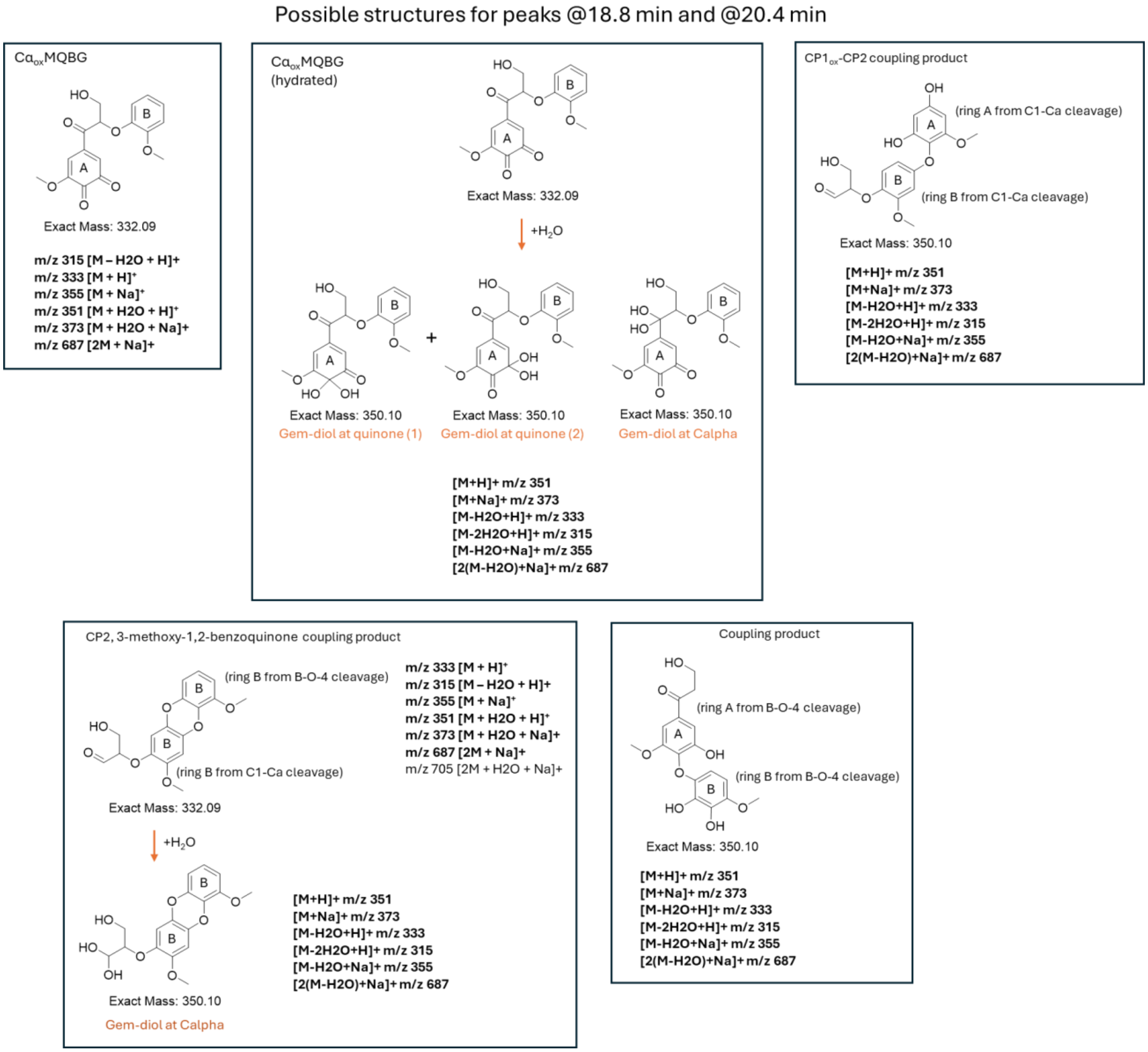

Figure S17. Hypothetical structures for the LC-PDA-MS (system 1) peaks at retention times of 18.8 and 20.4 min, as shown in Figure S16.

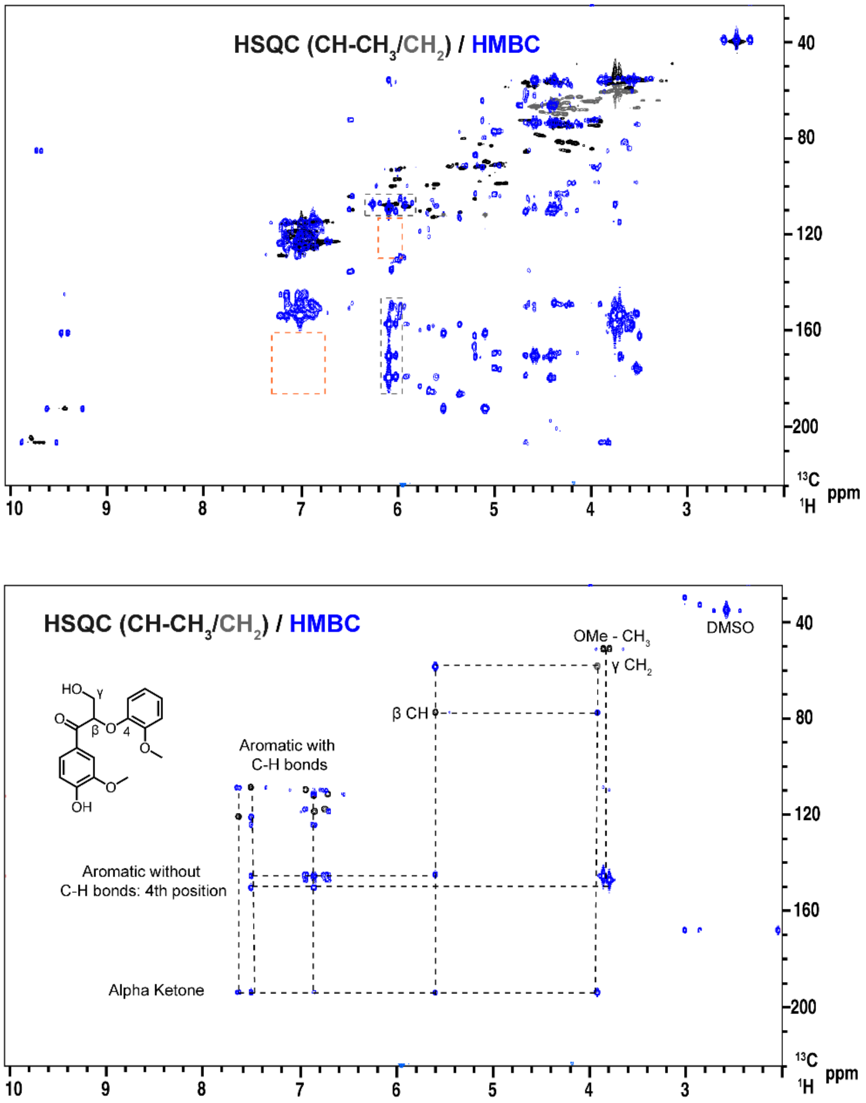

Figure S18 (continues below)

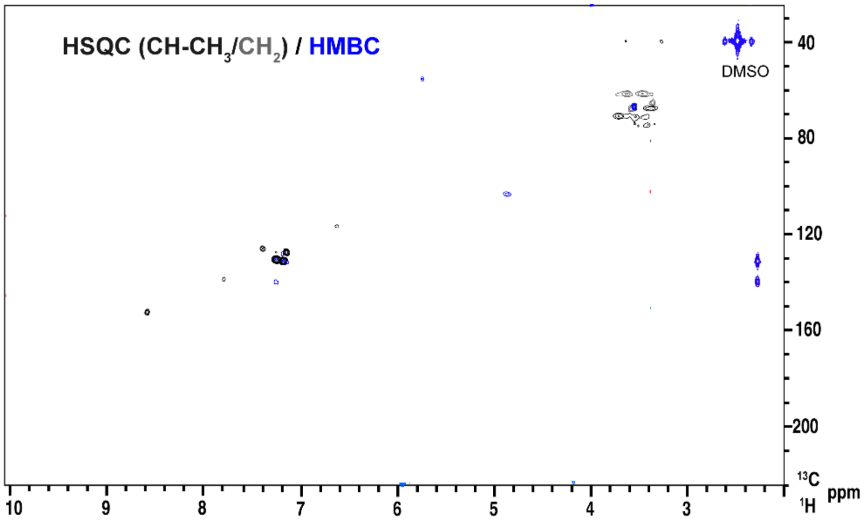

Figure S18. Upper panel: 2D ^{1}H–^{13}C HSQC and HMBC spectra of the pool of products of *Mt*PPO7 activity on GBG, with emphasis on the regions that suggest the presence of products with a Cα-ketone group conjugated with a quinone-like structure (such as $C\alpha_{ox}$MQBG, Cα-oxidized methoxy-*o*-quinone-glycerol-β-guaiacyl ether). Middle panel: 2D ^{1}H–^{13}C HSQC and HMBC spectra of $C\alpha_{ox}$GBG without enzyme treatment. Lower panel: 2D ^{1}H–^{13}C HSQC and HMBC spectra of *Mt*PPO7 protein sample.

The HSQC/HMBC spectrum of the reaction mixture reveals three distinct peaks around 6 ppm in the ^{1}H dimension and between 150–200 ppm in the ^{13}C dimension. These chemical shifts are characteristic of carbonyl-containing compounds, such as ketones or carboxylic acids. However, complementary ^{31}P NMR data confirms the really low abundance of carboxylic acid functionalities in the sample. This strongly suggests that the observed signals are more likely due to ketone groups.

To support this, we performed an HSQC/HMBC analysis of $C\alpha_{ox}$GBG (without enzyme treatment). These spectra clearly demonstrate a strong long-range coupling between the aromatic system and the carbonyl group, as evidenced by visible cross-peaks connecting the protons on the aromatic ring to the carbonyl carbon. To enhance the observation of these long-range correlations, we had to increase the d6 delay parameter in TopSpin – which governs the evolution time for long-range couplings – from the standard 50 ms to 100 ms. This adjustment allowed us to capture all relevant correlations more effectively.

In contrast, the current spectrum (of enzyme-treated GBG) lacks such correlations (orange squares), even at high d6 delays. This observation favors the presence of quinone-like structures, which could be consistent with the observed chemical shifts. Additionally, potential couplings with vinyl-like systems are suggested by signals in the 6–110 ppm range, further supporting the hypothesis of conjugated or extended π-systems typical of quinones.

The HSQC/HMBC spectrum of the *Mt*PPO7 protein in DMSO-d_6, shown here as a control, confirms that the protein itself does not contribute to the key peaks assigned in the experimental

spectra. Notably, the regions corresponding to aromatic (around 7–8 ppm ^{1}H / 120–140 ppm ^{13}C) and carbonyl (around 190–200 ppm ^{13}C) signals show minimal or no significant cross-peaks in this control sample.

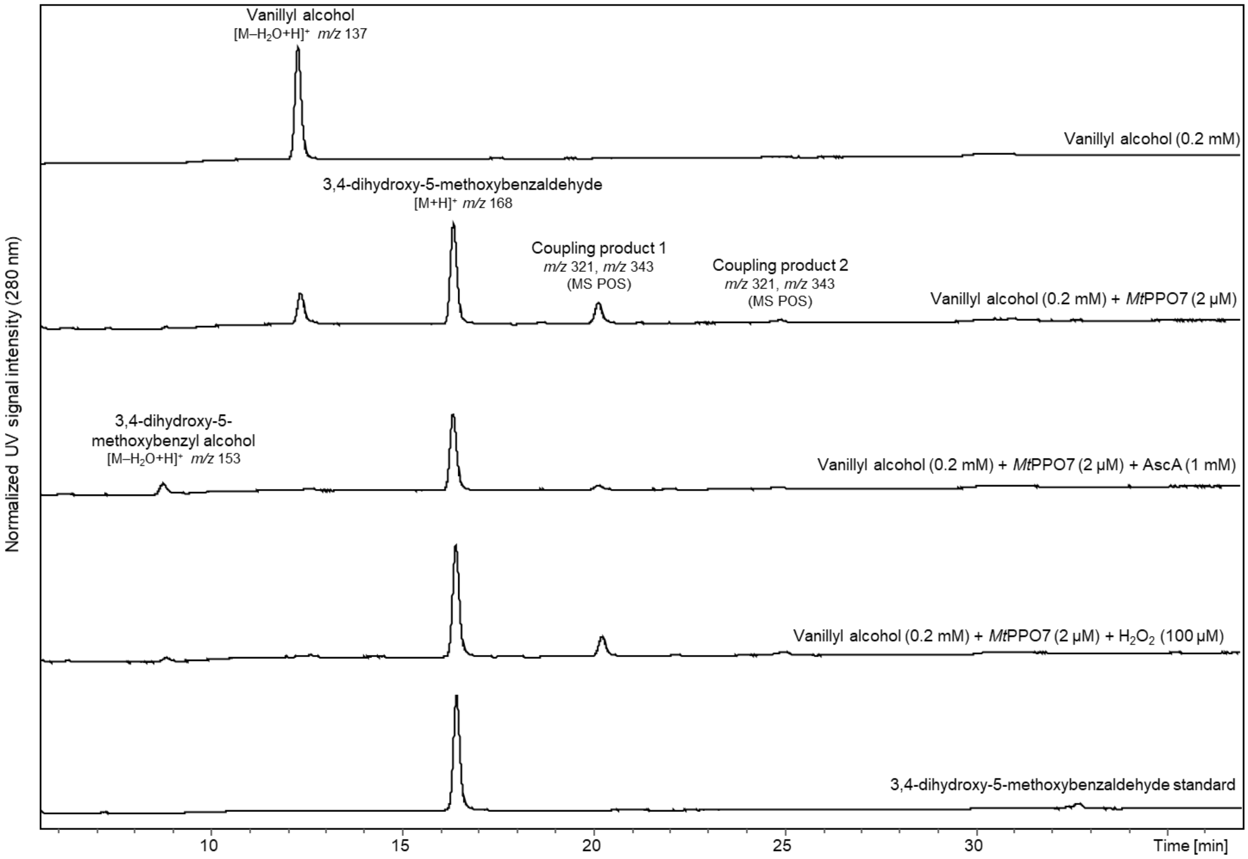

Figure S19. RP-UHPLC-PDA-ESI-MS chromatograms of the products of *Mt*PPO7 (2 µM) reaction on vanillyl alcohol (0.2 mM) in the presence or absence of ascorbic acid (1 mM) or hydrogen peroxide (100 µM). The chromatogram of an authentic standard of 3,4-dihydroxy-5-methoxybenzaldehyde is included. The chromatograms were obtained with system 2 described in Materials and Methods.

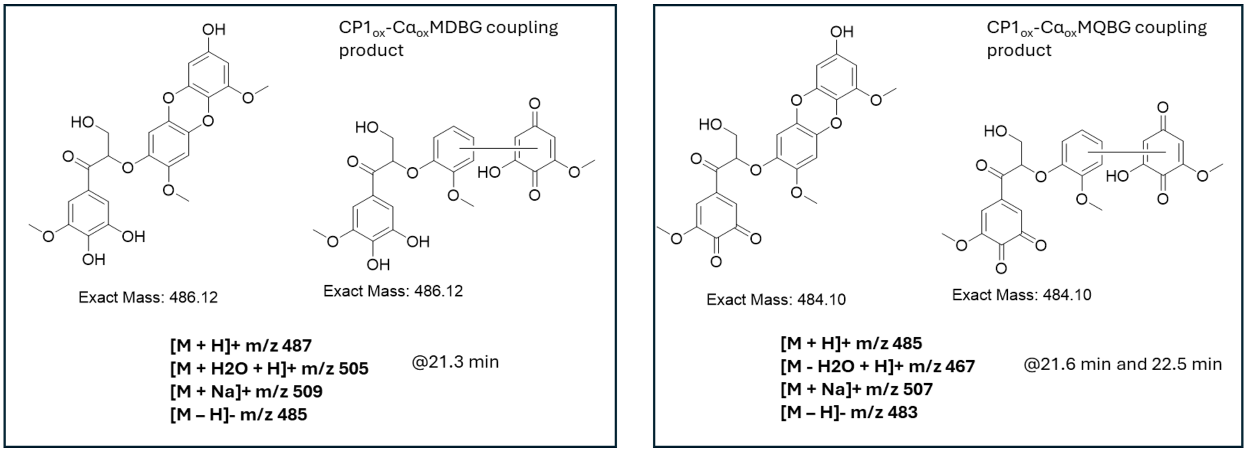

Figure S20. Hypothetical structures of coupling products generated by *Mt*PPO7 activity on GBG. Peak retention times correspond to LC-PDA-MS chromatograms obtained with system 1, as shown in Figure S16.

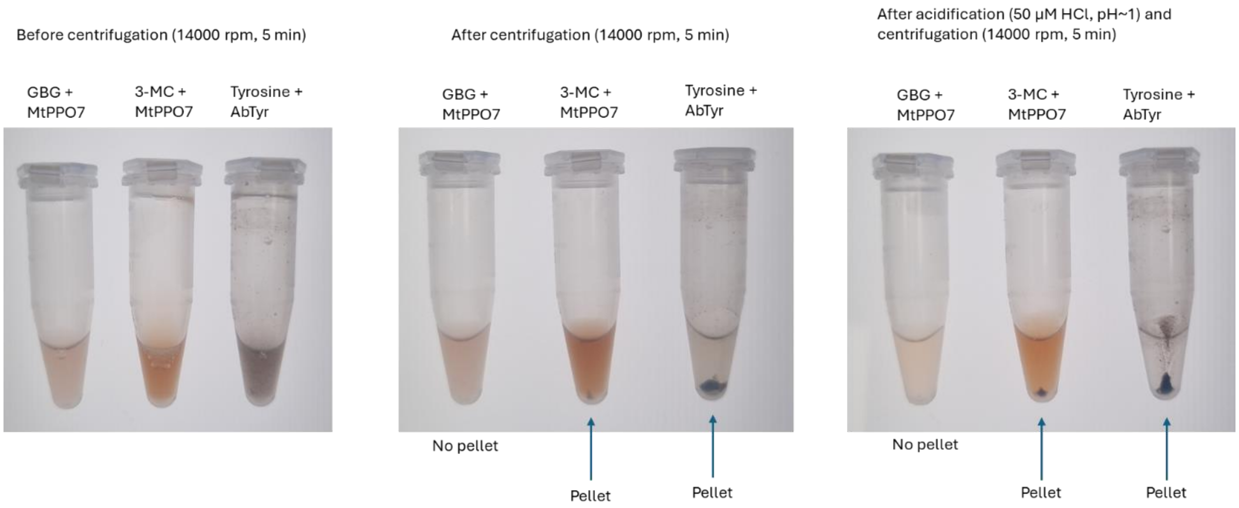

Figure S21. Visual inspection of the formation of water-insoluble products by *Mt*PPO7 activity on GBG and 3-methoxycatechol (3-MC) and by *Ab*Tyr on D/L-tyrosine. Reactions were performed using 1 mM of substrate and 5 µM of enzyme and incubated for 24 h at 30 °C and 1500 rpm in a Thermomixer. Samples were centrifuged (14000 rpm) before and after acidification with 50 µM HCl.

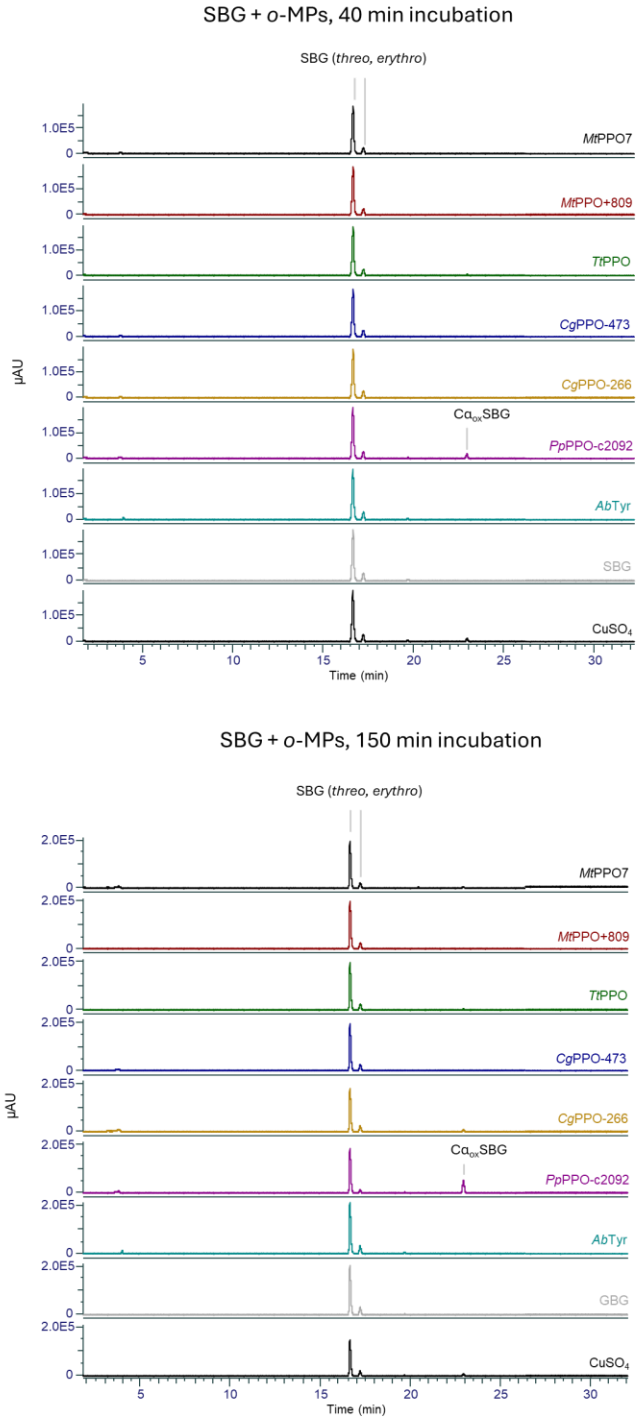

Figure S22. RP-UHPLC-PDA-ESI-MS chromatograms of the reaction products from *o*-MP-catalyzed oxidation of syringylglycerol-β-guaiacyl ether (SBG, 0.2 mM) after 40 min (upper panel) and 150 min (lower panel) of incubation. Control reactions with 10 µM $CuSO_4$, instead of enzyme, are also included. The traces correspond to absorbance at 280 nm. The analysis was performed using System 1 described in Materials and Methods.

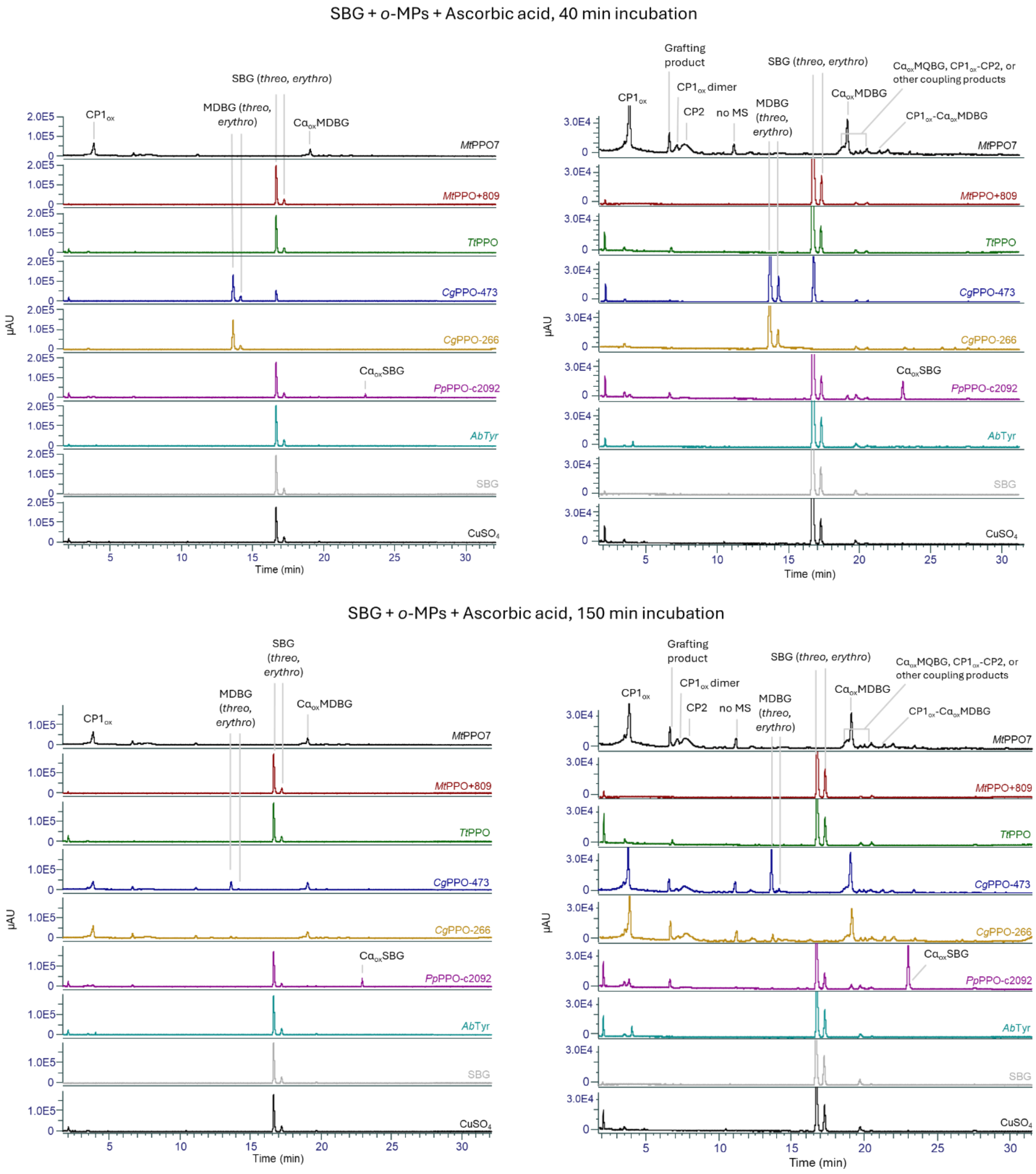

Figure S23. RP-UHPLC-PDA-ESI-MS chromatograms of the reaction products from *o*-MP-catalyzed oxidation of syringylglycerol-β-guaiacyl ether (SBG, 0.2 mM) in the presence of ascorbic acid (1 mM) after 40 min (upper panels) and 150 min (lower panels) of incubation. The chromatograms on the right are shown with a magnified Y-axis for better visualization of low-intensity signals, compared to those on the left. Control reactions with 10 µM $CuSO_4$, instead of

enzyme, are also included. The traces correspond to absorbance at 280 nm. The analysis was performed using System 1 described in Materials and Methods.

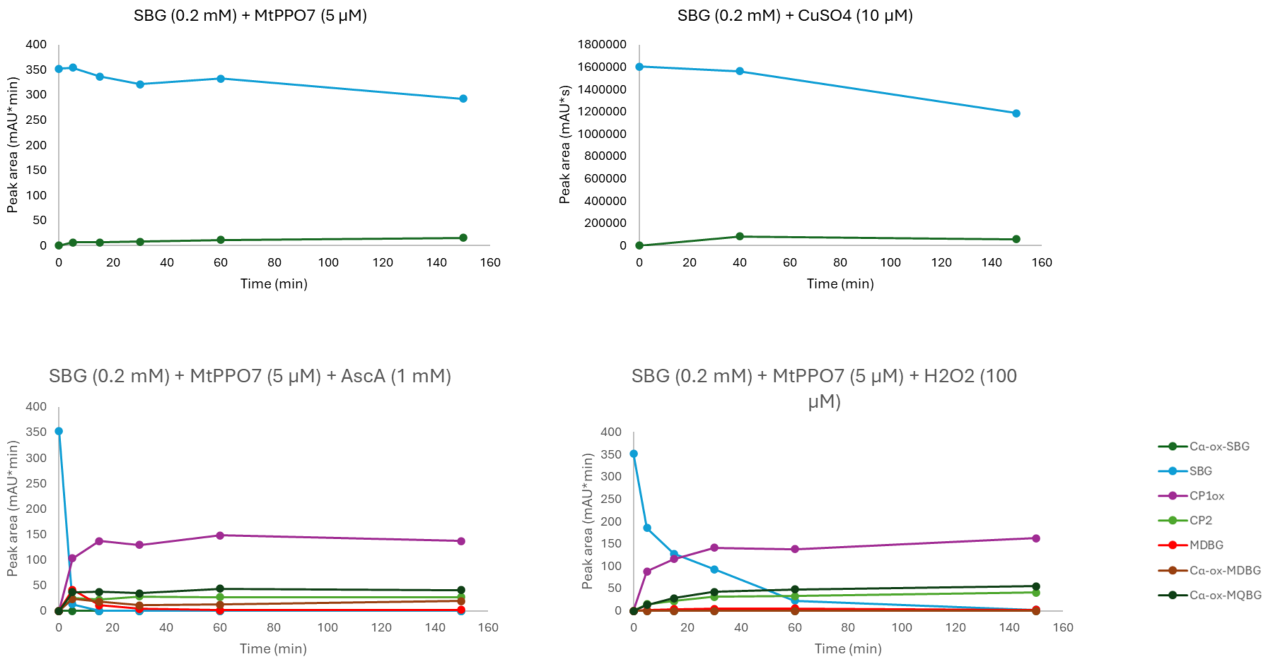

Figure S24. Time-course plots of *Mt*PPO7 (5 µM) activity on SBG (0.2 mM) in the absence or presence of ascorbic acid (1 mM) or hydrogen peroxide (100 µM). The relative quantification of the substrates and the main products was based on LC-PDA-MS peak areas at 280 nm (system 2 described in Materials and Methods).

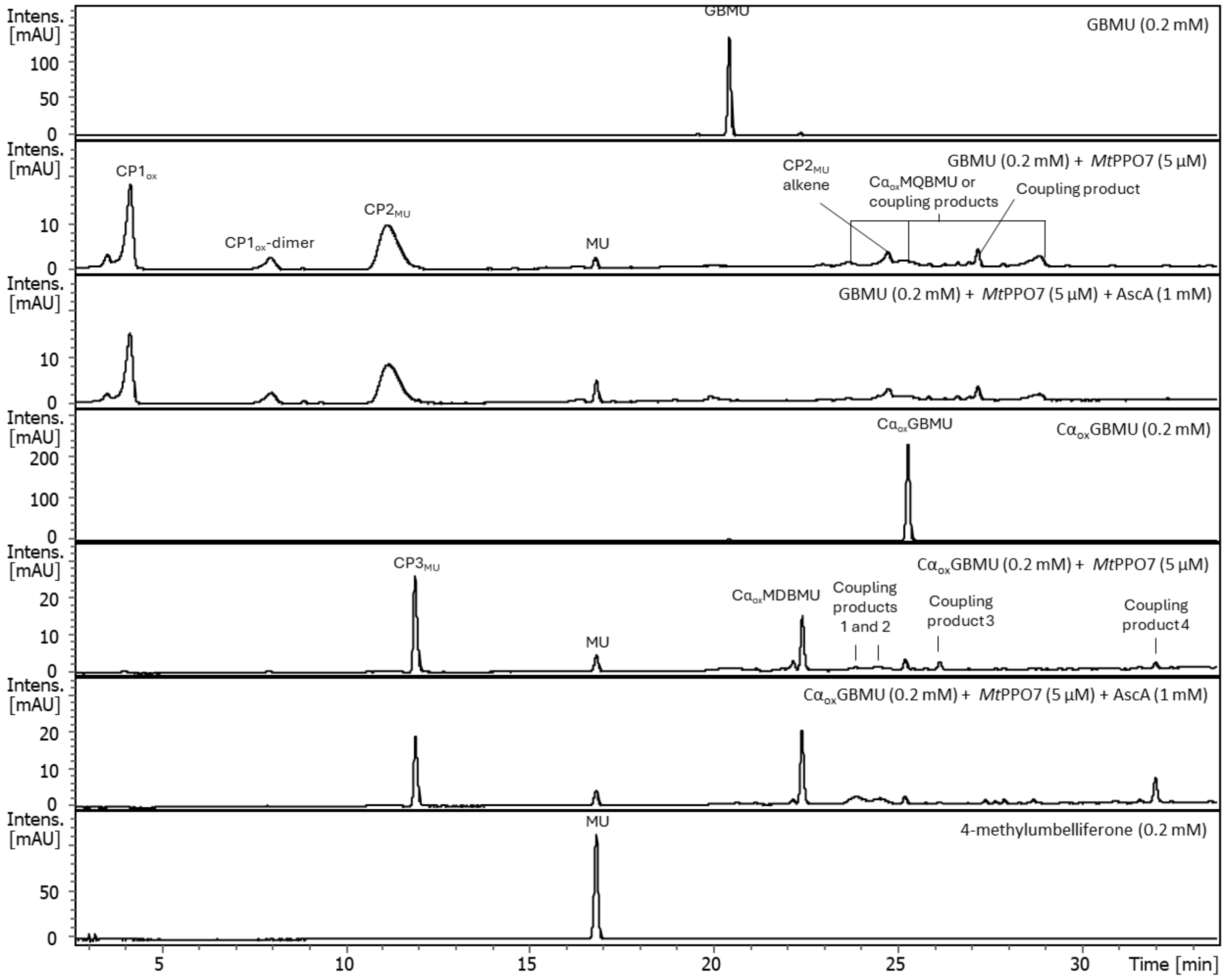

Figure S25. RP-UHPLC-PDA-ESI-MS chromatograms of the products of *Mt*PPO7 activity on guaiacylglycerol-β-4-methylumbelliferone (GBMU) and Cα-oxidized guaiacylglycerol-β-4-methylumbelliferone ($C\alpha_{ox}$GBMU) in the presence or absence of ascorbic acid (AscA, 1 mM) for 150 min. An authentic standard of 4-methylumbelliferone (MU) is also included. The traces correspond to absorbance at 280 nm. Chromatograms were obtained with system 2 described in Materials and Methods. Tentative structures for the main coupling products are proposed in Figures S26 and S27. Using a calibration curve for MU (LC-UV-vis), we quantified the released MU in the reactions and found concentrations of 4.9 µM and 8.2 µM of MU from GBMU (without and with ascorbic acid, respectively), and 6.9 µM and 9.8 µM of MU from $C\alpha_{ox}$-GBMU (without and with ascorbic acid, respectively). Given an initial substrate concentration of 0.2 mM, these values correspond to yields of approximately 2.5 – 5% for both substrates, indicating that β-O-4 bond cleavage is a minor reaction pathway compared to C1–Cα cleavage.

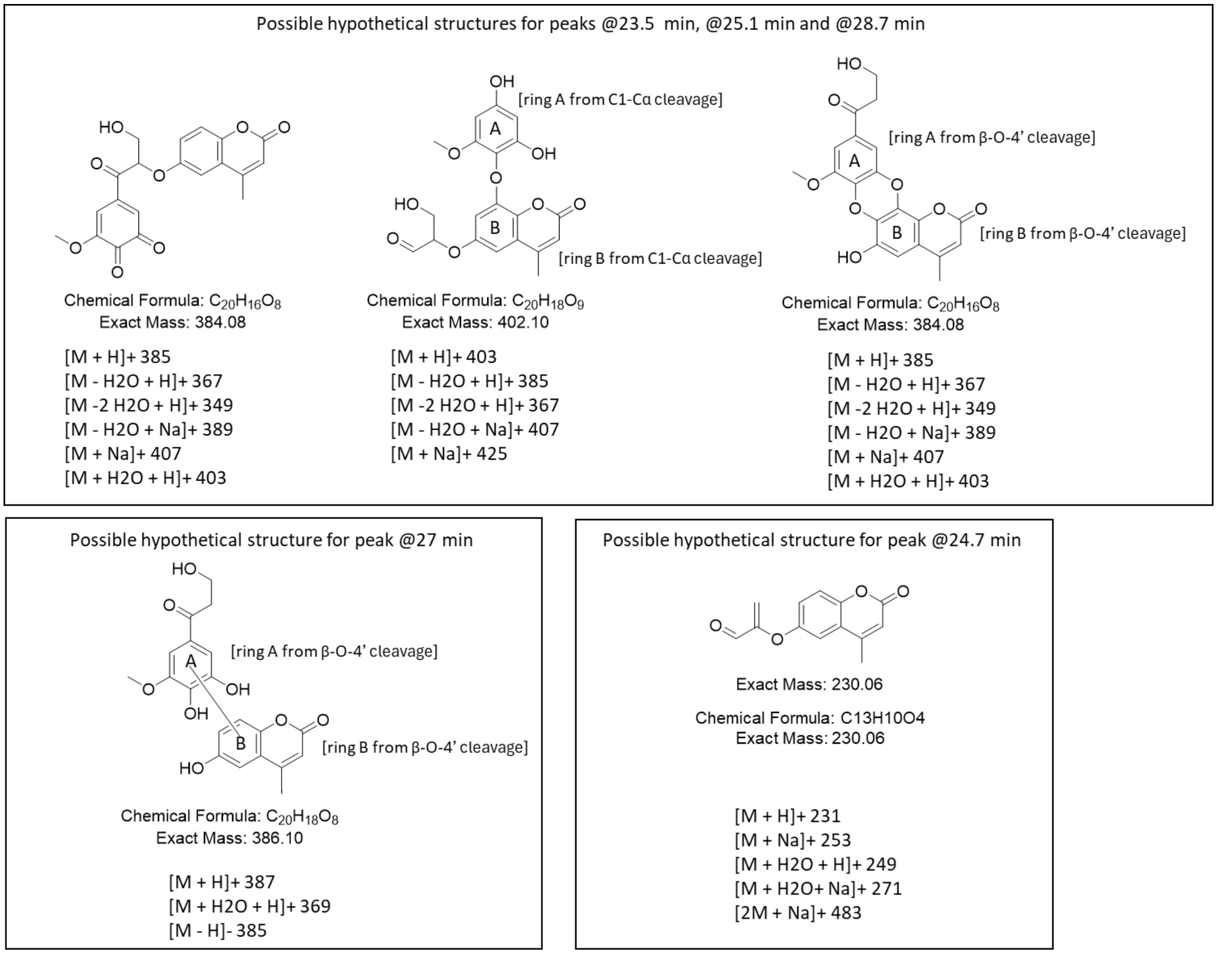

Figure S26. Hypothetical structures of some coupling products or cleavage products generated by *Mt*PPO7 activity towards GBMU. Peak retention times correspond to LC-PDA-MS chromatograms obtained with system 2, as shown in Figure S25.

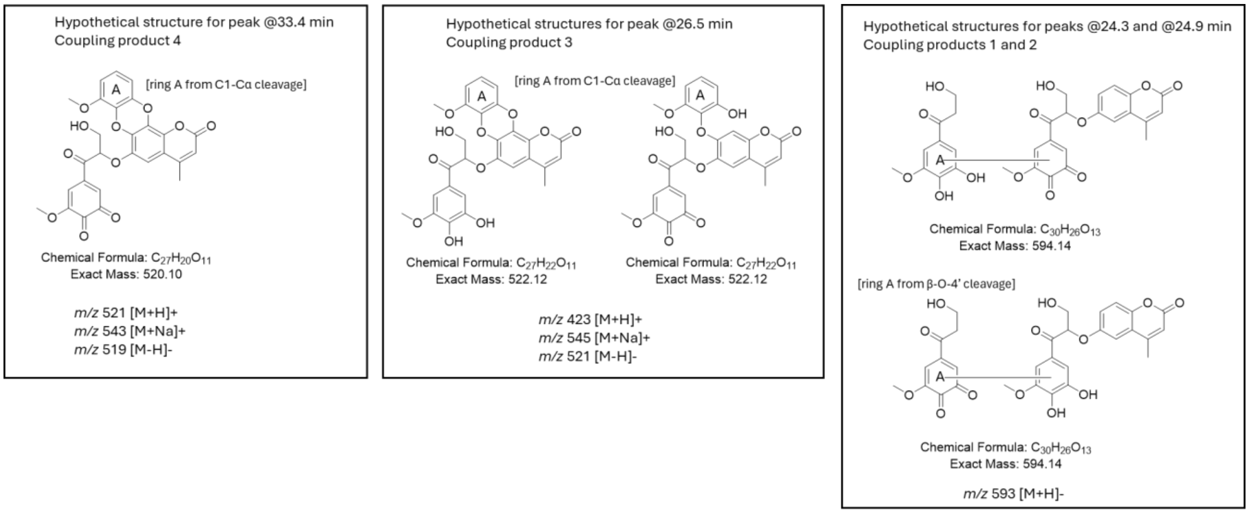

Figure S27. Hypothetical structures of some coupling products generated by *Mt*PPO7 activity towards $C\alpha_{ox}$GBMU. Peak retention times correspond to LC-PDA-MS chromatograms obtained with system 2, as shown in Figure S25.

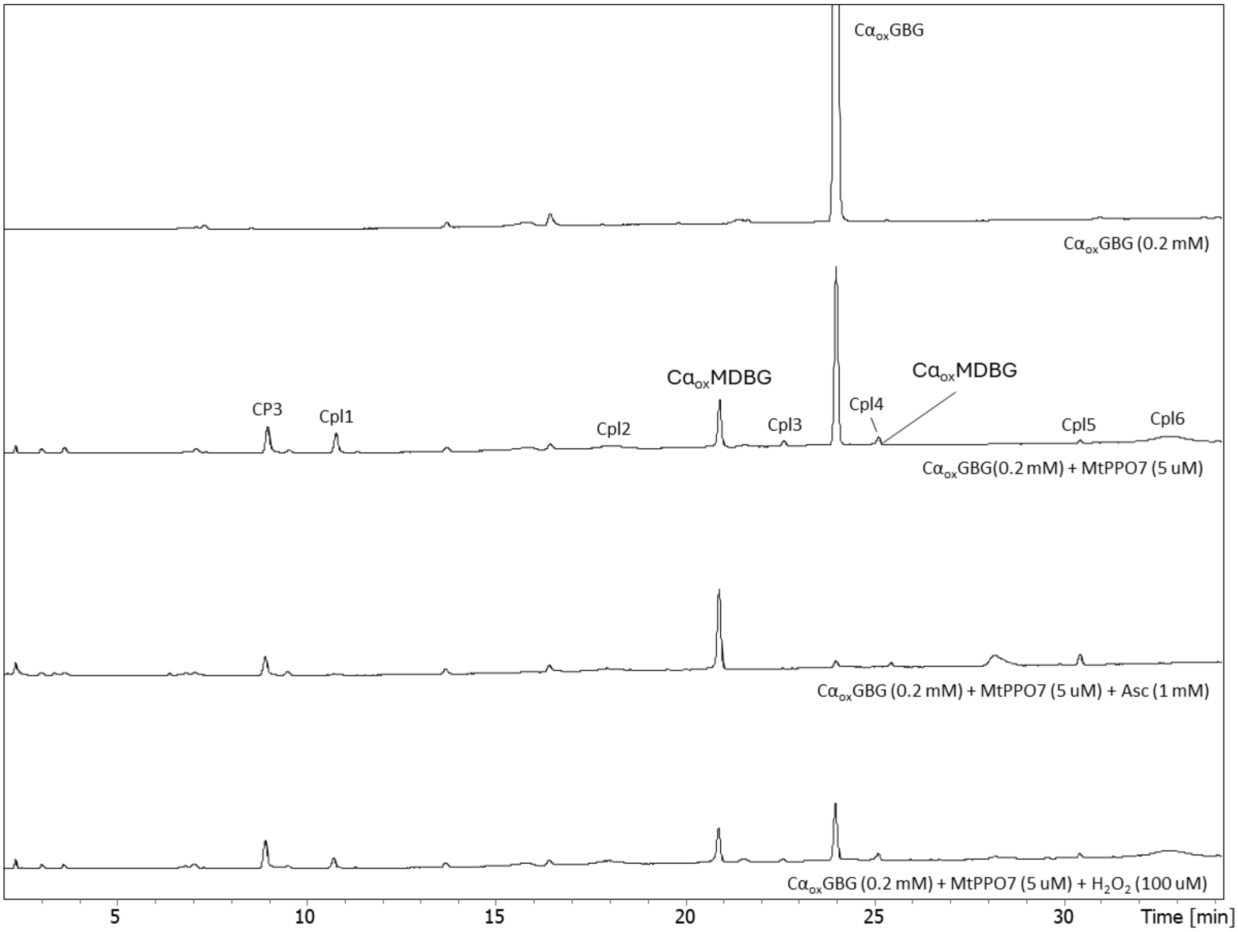

Figure S28. RP-UHPLC-PDA-ESI-MS chromatograms of the products of *Mt*PPO7 activity on $C\alpha_{ox}$GBG in the presence or absence of ascorbic acid (1 mM) or hydrogen peroxide (100 µM) for 150 min. The traces correspond to absorbance at 280 nm. The hypothetical structure of coupling products (Cpl1-6) can be found in Figure S29. Chromatograms were obtained with system 2 described in Materials and Methods.

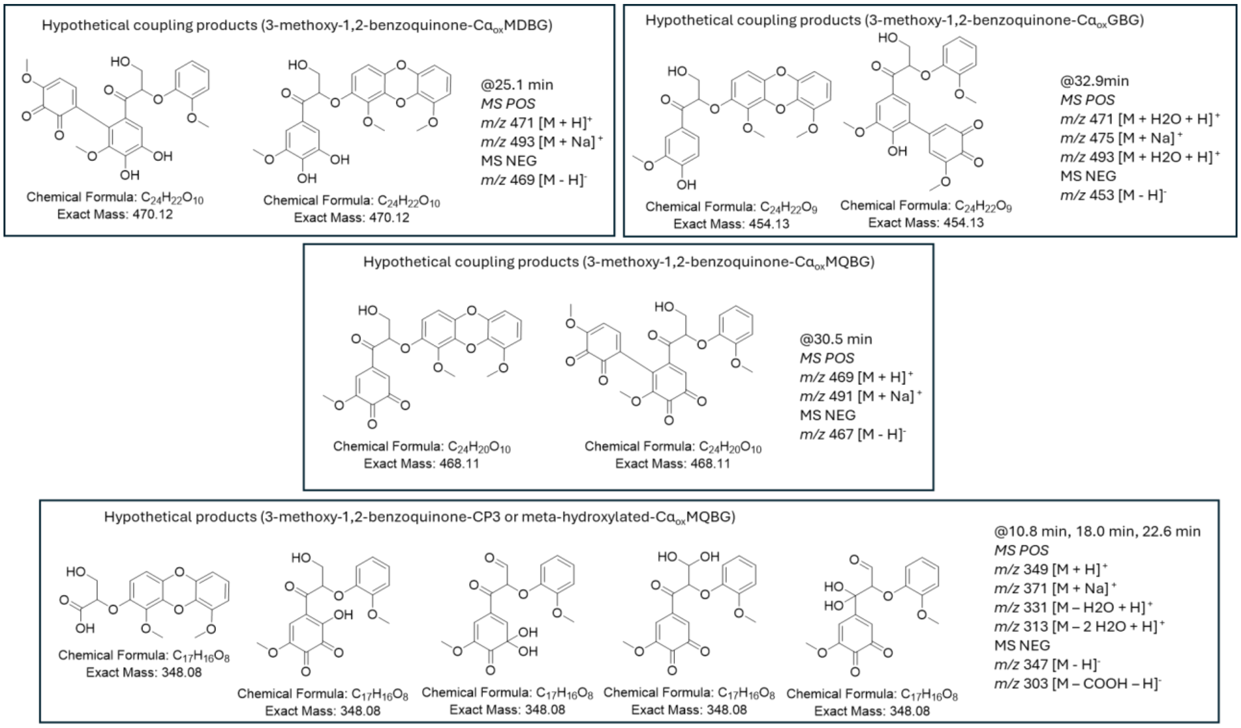

Figure S29. Hypothetical structures of coupling products generated during *Mt*PPO7 reaction towards $C\alpha_{ox}$GBG, based on RP-UHPLC-PDA-ESI-MS data generated with system 2 described in Materials and Methods, as shown in Figure S28.

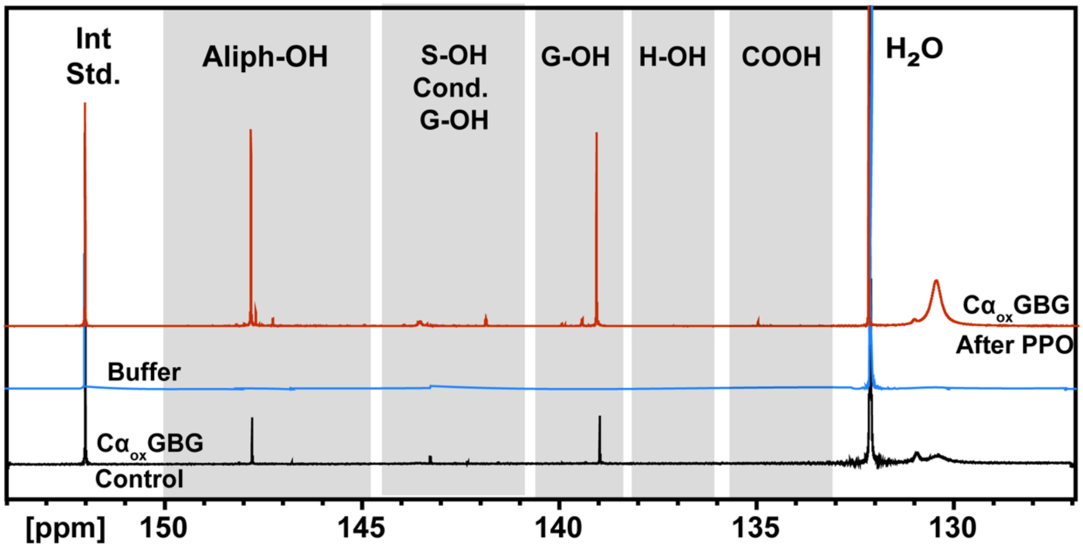

Figure S30. ^{31}P NMR spectra of the pool of products of *Mt*PPO7 activity on Cα$_{ox}$GBG (red) in comparison with Cα$_{ox}$GBG incubated with buffer (black) and a buffer-only spectra (blue).

A

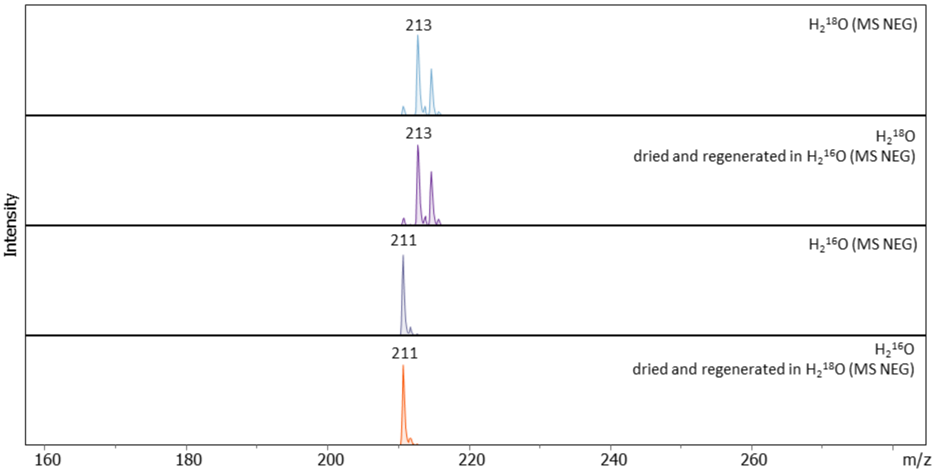

B

Solvent used in the reaction	$H_2{}^{16}O$	$H_2{}^{18}O$	$H_2{}^{18}O$	$H_2{}^{16}O$
Additional step	-	-	Drying and regeneration in $H_2{}^{16}O$	Drying and regeneration in $H_2{}^{18}O$
m/z 211 (unlabeled CP3)	100	12	10	100
m/z 213 (mono-^{18}O labeled CP3)	2	100	100	1
m/z 215 (di-^{18}O labeled CP3)	0	73	68	0

Figure S31. Study on the incorporation of ^{18}O from $H_2{}^{18}O$ in the cleavage product CP3 from $C\alpha_{ox}$GBG upon reaction with *Mt*PPO7. (A) MS-NEG spectra of CP3 ($[M-H]^-$) generated in the presence of $H_2{}^{16}O$ or $H_2{}^{18}O$. Reaction products were analyzed directly after enzyme inactivation or vacuum-dried and regenerated in $H_2{}^{18}O$ or $H_2{}^{16}O$ before MS analysis, as indicated in the figure. (B) Relative intensities (%) of the molecular ions $[M-H]^-$ corresponding to CP3.

The retention of ^{18}O in CP3 after vacuum-drying and resuspension in $H_2{}^{16}O$, along with the absence of ^{18}O incorporation when reacted in $H_2{}^{16}O$, dried, and resuspended in $H_2{}^{18}O$, corroborates that the ^{18}O incorporation is due to water attack to an enzymatic reaction intermediate product and not simply due to isotope exchange with water.

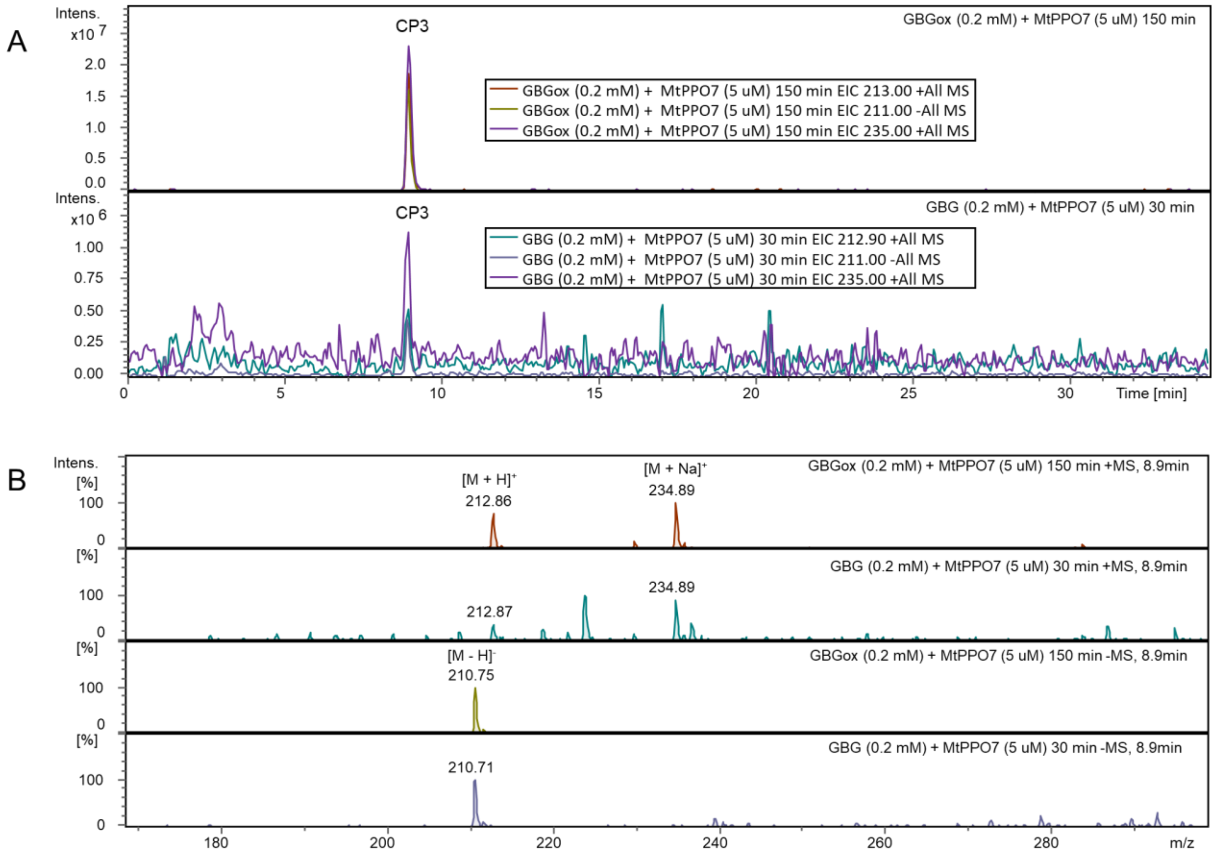

Figure S32. Detection of CP3 in *Mt*PPO7 reactions with Cα_{ox}GBG and GBG. (A) Extracted ion chromatograms of *m/z* 211 ([M-H]$^-$), *m/z* 213 ([M+H]$^+$), and *m/z* 235 ([M+Na]$^+$) ions corresponding to CP3. (B) MS1 spectra of peaks corresponding to CP3 in positive and negative ion modes. RP-UHPLC-PDA-ESI-MS data was generated with system 2 described in Materials and Methods.

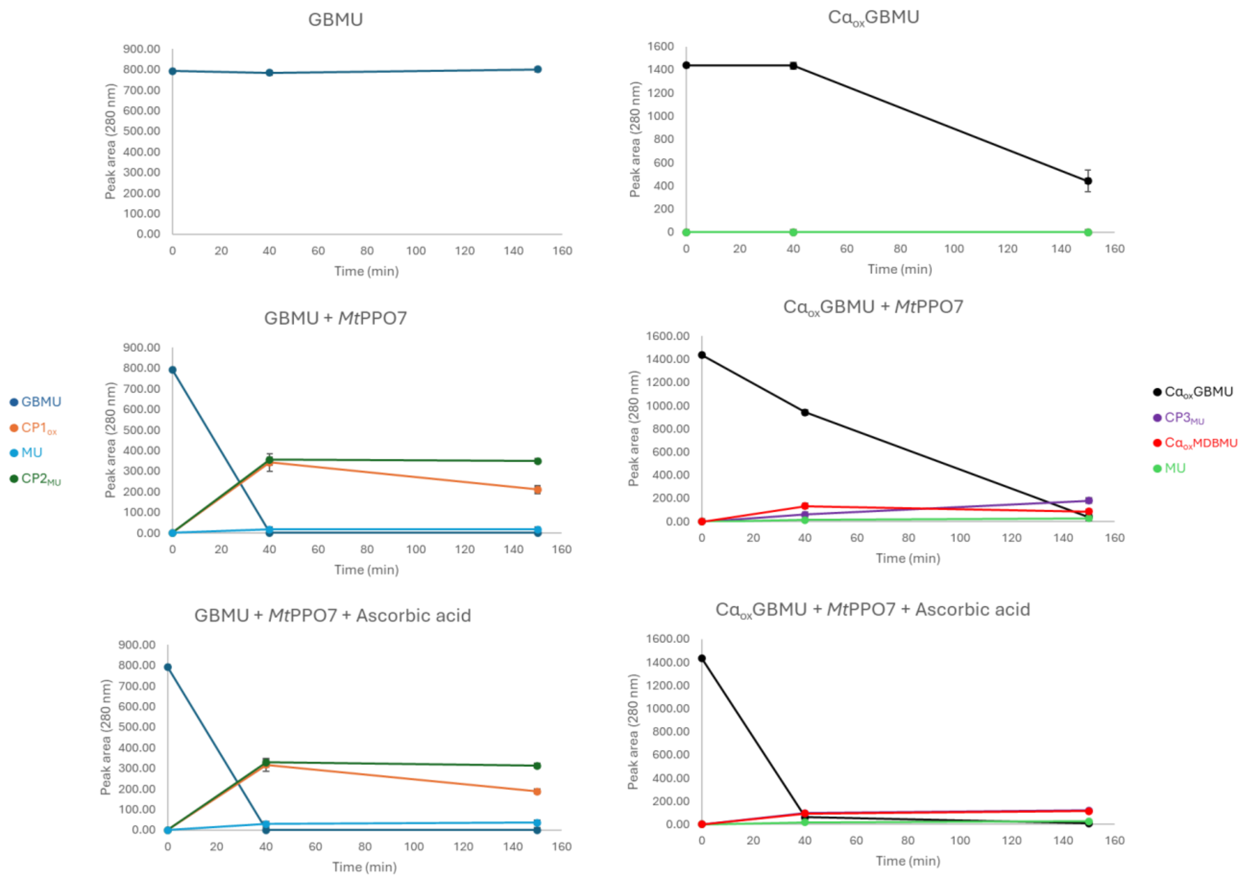

Figure S33. Time course of *Mt*PPO7 activity on GBMU and $C\alpha_{ox}$GBMU and the relative abundance of the main products. The relative quantification of the substrates and the main products was based on UHPLC-PDA-MS peak areas at 280 nm. Note: The drop in $C\alpha_{ox}$GBMU concentration in the absence of enzyme may be related to a drop in solubility over time.

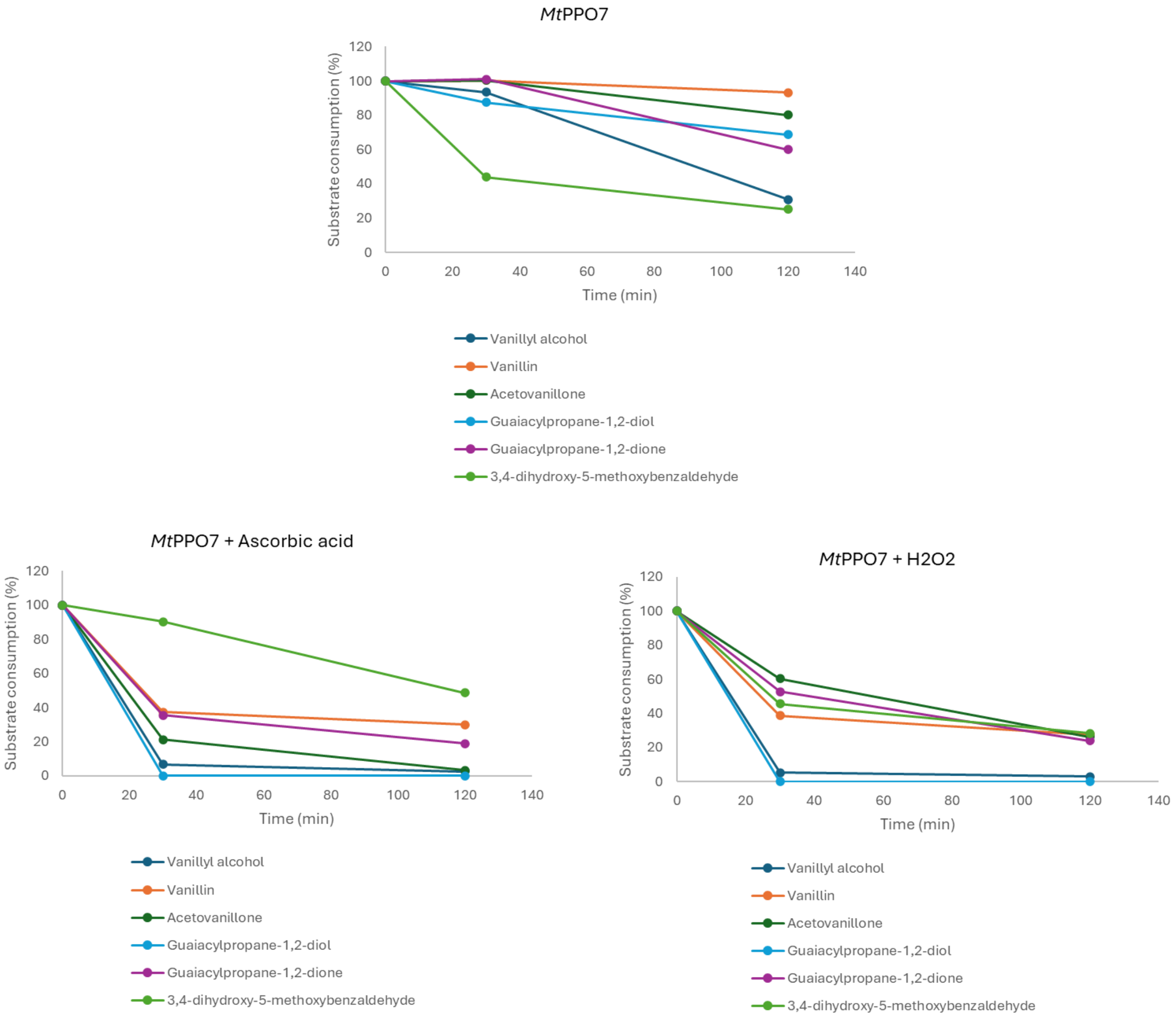

Figure S34. *Mt*PPO7 activity (2 µM) on monomeric guaiacyl-type compounds (0.2 mM) in the presence or absence of ascorbic acid (1 mM) or hydrogen peroxide (100 µM). Substrate consumption was measured by UHPLC-PDA-MS using peak areas at λ 280 nm (system 2, as described in Materials and Methods).

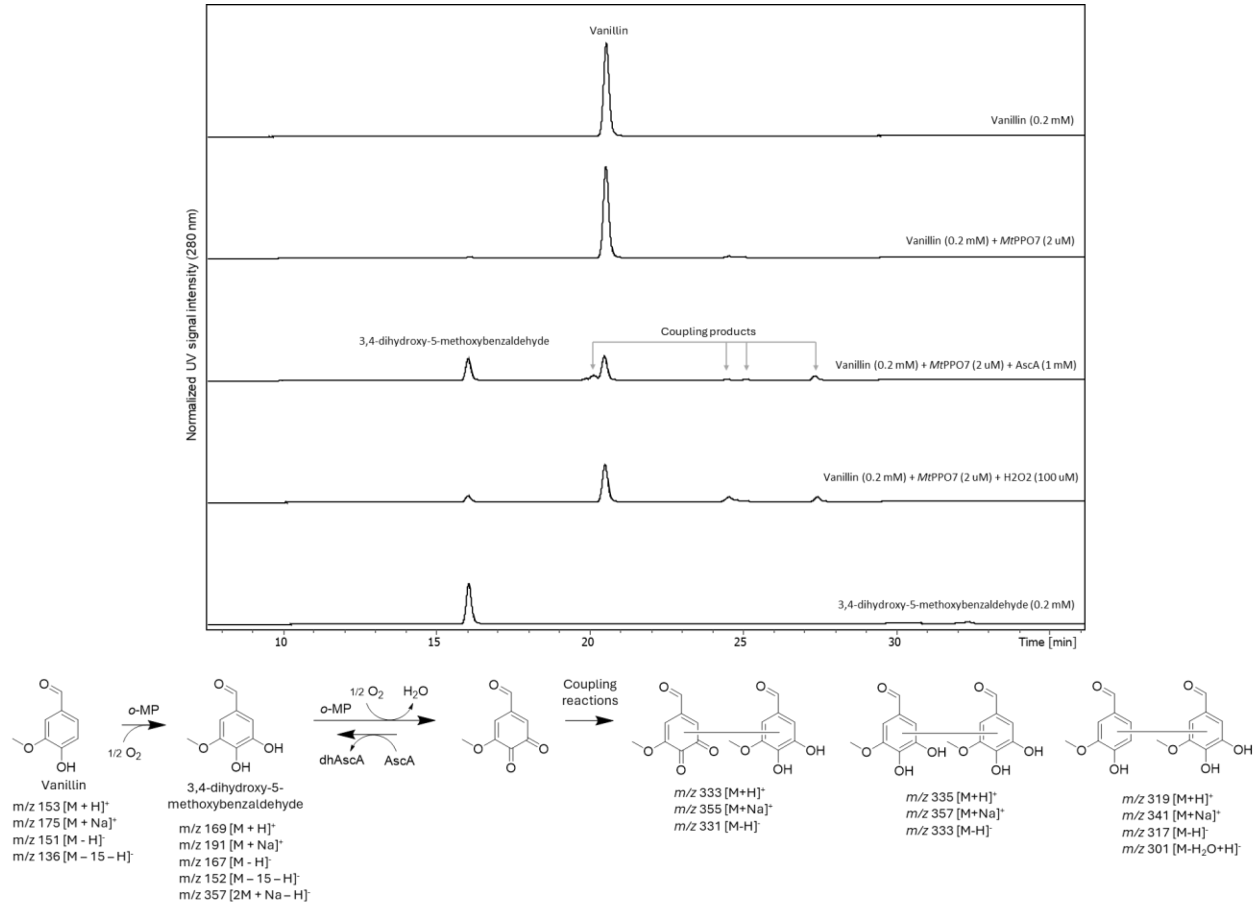

Figure S35. RP-UHPLC-PDA-ESI-MS chromatograms of *Mt*PPO7 (2 µM) reaction towards vanillin (0.2 mM) in the absence or presence of ascorbic acid (1 mM) or hydrogen peroxide (100 µM) for 120 min. The chromatogram of an authentic standard of 3,4-dihydroxy-5-methoxybenzaldehyde is included. Traces correspond to LC-UV absorbances at 280 nm (obtained with system 2, as described in Materials and Methods).

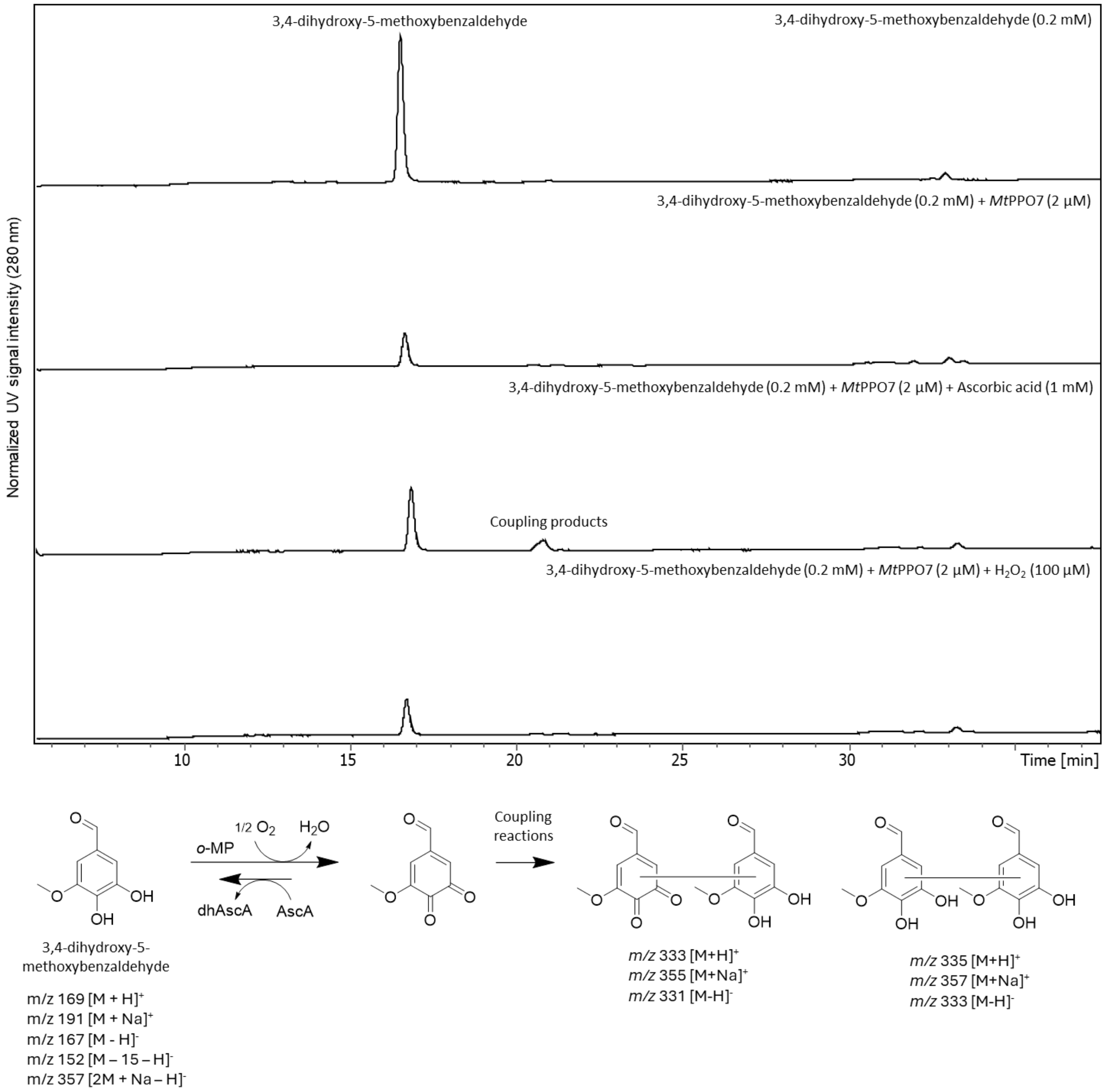

Figure S36. RP-UHPLC-PDA-ESI-MS chromatograms of *Mt*PPO7 (2 µM) reaction towards 3,4-dihydroxy-5-methoxybenzaldehyde (0.2 mM) in the absence or presence of ascorbic acid (1 mM) or hydrogen peroxide (100 µM) for 120 min. Traces correspond to LC-UV absorbances at 280 nm (obtained with system 2, as described in Materials and Methods).

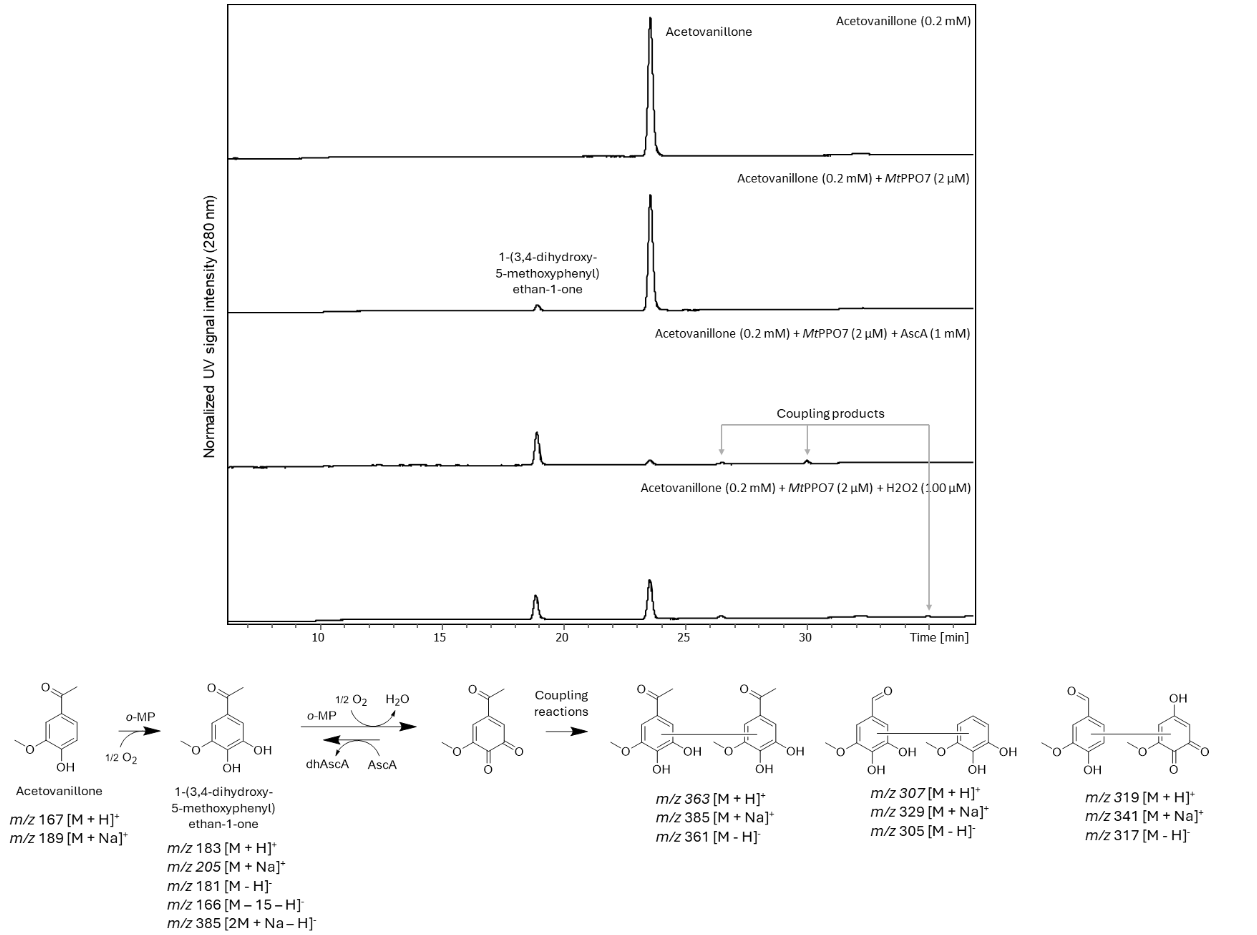

Figure S37. RP-UHPLC-PDA-ESI-MS chromatograms of *Mt*PPO7 (2 µM) reaction towards acetovanillone (0.2 mM) in the absence or presence of ascorbic acid (1 mM) or hydrogen peroxide (100 µM) for 120 min. Traces correspond to LC-UV absorbances at 280 nm (obtained with system 2, as described in Materials and Methods).

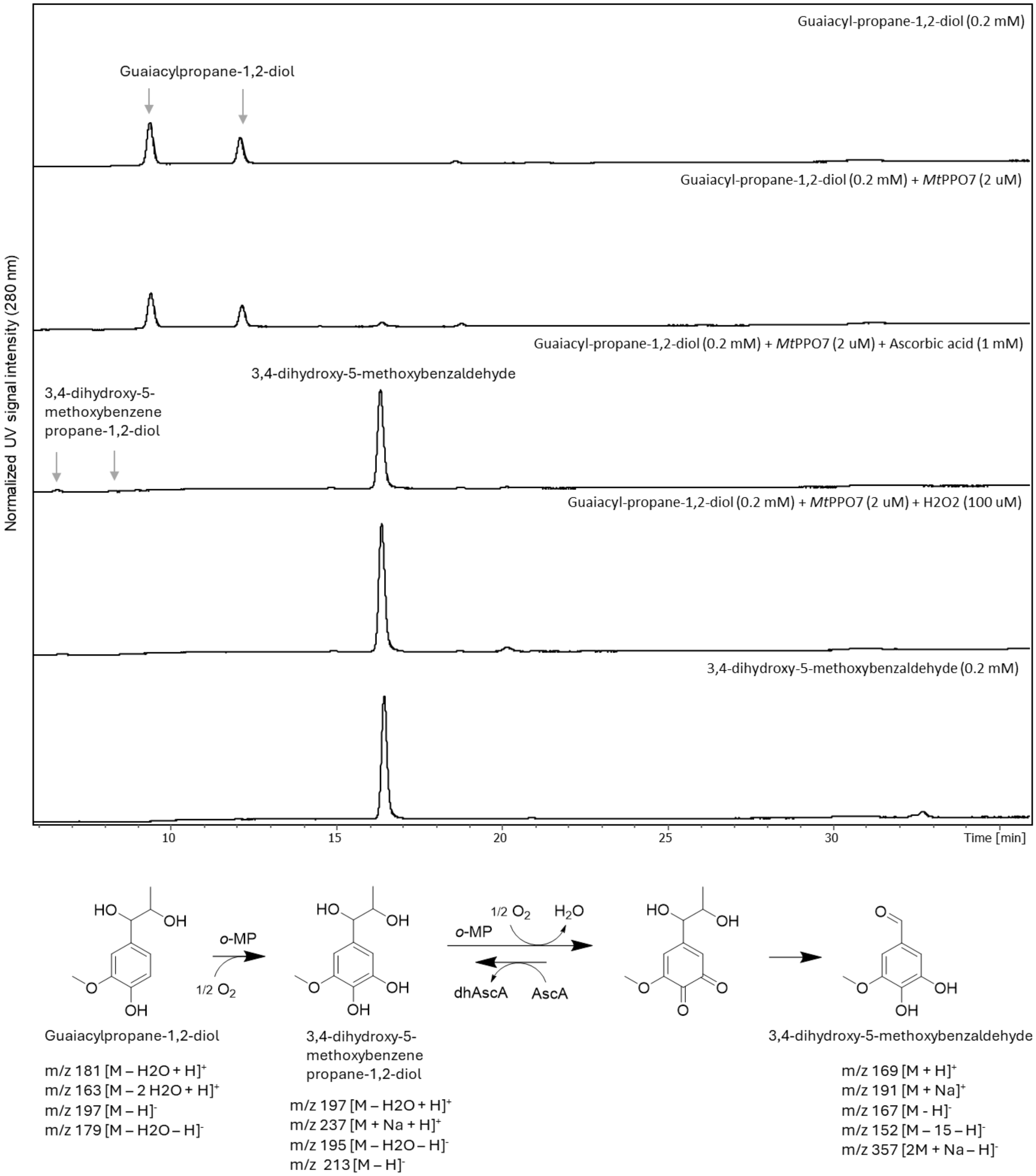

Figure S38. RP-UHPLC-PDA-ESI-MS chromatograms of *Mt*PPO7 (2 µM) reaction towards guaiacylpropane-1,2-diol (0.2 mM) in the absence or presence of ascorbic acid (1 mM) or hydrogen peroxide (100 µM) for 120 min. The chromatogram of an authentic standard of 3,4-dihydroxy-5-methoxybenzaldehyde is included. Traces correspond to LC-UV absorbances at 280 nm (obtained with system 2, as described in Materials and Methods).

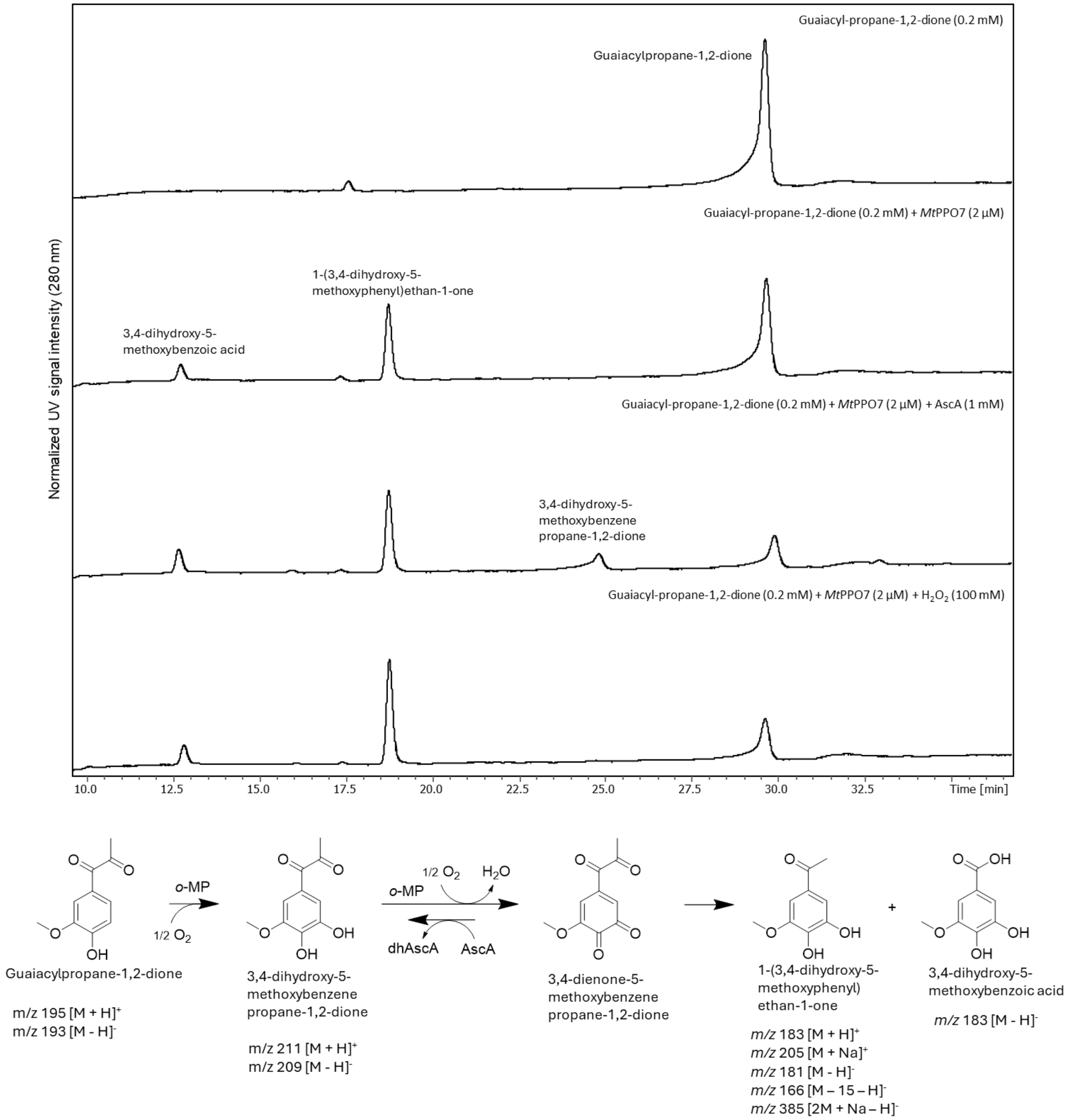

Figure S39. RP-UHPLC-PDA-ESI-MS chromatograms of *Mt*PPO7 (2 µM) reaction towards on guaiacylpropane-1,2-dione (0.2 mM) in the absence or presence of ascorbic acid (1 mM) or hydrogen peroxide (100 µM) for 120 min. Traces correspond to LC-UV absorbances at 280 nm (obtained with system 2, as described in Materials and Methods).

SI tables

Table S1. Monophenolase and diphenolase activities of *Mt*PPO7 and *Cg*PPO-473 on vanillic acid and 3,4-dihydroxy-5-methoxybenzoic acid, respectively. Specific activities were measured by substrate consumption over time using RP-UHPLC-PDA-ESI-MS (system 3, described in Materials and Methods).

		pH 4.0	pH 5.0	pH 6.0	pH 7.0	pH 8.0	pH 9.0
*Mt*PPO7	Monophenolase activity [U/µmol]	2.0 ± 2.8	10.0 ± 0.6	10.4 ± 1.6	15.6 ± 0.9	11.0 ± 0.9	7.2 ± 2.7
	Diphenolase activity [U/µmol]	20.1 ± 5.0	29.6 ± 1.2	39.3 ± 0.8	34.9 ± 1.4	21.5 ± 0.8	33.0 ± 1.4
	Monophenolase-to-diphenolase ratio	0.1	0.34	0.26	0.45	0.51	0.22
*Cg*PPO-473	Monophenolase activity [U/µmol]	0.4 ± 0.1	6.1 ± 0.6	11.7 ± 0.1	11.0 ± 0.1	6.5 ± 1.3	7.2 ± 2.7
	Diphenolase activity [U/µmol]	21.4 ± 0.6	19.3 ± 2.1	20.7 ± 1.6	25.8 ± 3.2	23.0 ± 0.6	35.6 ± 7.7
	Monophenolase-to-diphenolase ratio	0.02	0.31	0.56	0.43	0.28	0.2

Table S2. ^{31}P NMR quantification of aliphatic, phenolic and carboxylic hydroxyl groups of dimer model compounds. Aliph-OH refers to the aliphatic hydroxyl groups, G-OH refers to phenolic groups of guaiacol units, H-OH refers to phenolic groups of hydroxyphenylpropane units and COOH refers to carboxylic acid groups. Sample 1: $C\alpha_{ox}$GBG after enzymatic treatment in buffer, Sample 2: $C\alpha_{ox}$GBG before enzymatic treatment in buffer, Sample 3: buffer.

Samples	Aliph.-OH [mmol.g^{-1}]	G-OH [mmol.g^{-1}]	H-OH [mmol.g^{-1}]	COOH [mmol.g^{-1}]
1	3.082	2.666	0	0.072
2	3.067	2.939	0	0
3	0.026	0	0	0

SI References

(1) Fache, M.; Darroman, E.; Besse, V.; Auvergne, R.; Caillol, S.; Boutevin, B. Vanillin, a Promising Biobased Building-Block for Monomer Synthesis. *Green Chem.* **2014**, *16* (4), 1987–1998. https://doi.org/10.1039/C3GC42613K.

(2) Perez, J. M.; Kontur, W. S.; Alherech, M.; Coplien, J.; Karlen, S. D.; Stahl, S. S.; Donohue, T. J.; Noguera, D. R. Funneling Aromatic Products of Chemically Depolymerized Lignin into 2-Pyrone-4-6-Dicarboxylic Acid with Novosphingobium aromaticivorans. *Green Chem.* **2019**, *21* (6), 1340–1350. https://doi.org/10.1039/C8GC03504K.

(3) Weinstein, D. A.; Gold, M. H. Synthesis of Guaiacylglycol and Glycerol-β-O-(β-Methylumbelliferyl) Ethers: Lignin Model Substrates for the Possible Fluorometric Assay of β-Etherases. *Holzforschung* **1979**, *33*, 134–135.

(4) Oates, N. C.; Abood, A.; Schirmacher, A. M.; Alessi, A. M.; Bird, S. M.; Bennett, J. P.; Leadbeater, D. R.; Li, Y.; Dowle, A. A.; Liu, S.; Tymokhin, V. I.; Ralph, J.; McQueen-Mason, S. J.; Bruce, N. C. A Multi-Omics Approach to Lignocellulolytic Enzyme Discovery Reveals a New Ligninase Activity from Parascedosporium putredinis NO1. *Proc. Natl. Acad. Sci. U. S. A.* **2021**, *118* (18), e2008888118. https://doi.org/10.1073/pnas.2008888118.

(5) Silva, C. de O. G.; Sun, P.; Barrett, K.; Sanders, M. G.; Van Berkel, W. J. H.; Kabel, M. A.; Meyer, A. S.; Agger, J. W. Polyphenol Oxidase Activity on Guaiacyl and Syringyl Lignin Units. *Angew. Chemie Int. Ed.* **2024**, *63* (48), e202409324. https://doi.org/10.1002/ANIE.202409324.

(6) Kuijpers, T. F. M.; Gruppen, H.; Sforza, S.; Van Berkel, W. J. H.; Vincken, J. P. The Antibrowning Agent Sulfite Inactivates Agaricus bisporus Tyrosinase through Covalent Modification of the Copper-B Site. *FEBS J.* **2013**, *280* (23), 6184–6195. https://doi.org/10.1111/FEBS.12539.

(7) Meng, X.; Crestini, C.; Ben, H.; Hao, N.; Pu, Y.; Ragauskas, A. J.; Argyropoulos, D. S. Determination of Hydroxyl Groups in Biorefinery Resources via Quantitative 31P NMR Spectroscopy. *Nat. Protoc. 2019 149* **2019**, *14* (9), 2627–2647. https://doi.org/10.1038/s41596-019-0191-1.
